# 2-Thiopyrimidine/chalcone hybrids: design, synthesis, ADMET prediction, and anticancer evaluation as STAT3/STAT5a inhibitors

**DOI:** 10.1080/14756366.2020.1740922

**Published:** 2020-03-25

**Authors:** Phoebe F. Lamie, John N. Philoppes

**Affiliations:** Department of Pharmaceutical Organic Chemistry, Faculty of Pharmacy, Beni-Suef University, Beni-Suef, Egypt

**Keywords:** 2-Thiopyrimidine, chalcone, STAT, cytotoxicity, computational analysis

## Abstract

A novel 2-thiopyrimidine/chalcone hybrid was designed, synthesised, and evaluated for their cytotoxic activities against three different cell lines, K-562, MCF-7, and HT-29. The most active cytotoxic derivatives were **9d**, **9f**, **9n**, and **9p** (IC_50_=0.77–1.74 µM, against K-562 cell line), **9a** and **9r** (IC_50_=1.37–3.56 µM against MCF-7 cell line), and **9a**, **9l**, and **9n** (IC_50_=2.10 and 2.37 µM against HT-29 cell line). Compounds **9a**, **9d**, **9f**, **9n**, and **9r** were further evaluated for their cytotoxicity against normal fibroblast cell line WI38. Moreover, STAT3 and STAT5a inhibitory activities were determined for the most active derivatives **9a**, **9d**, **9f**, **9n**, and **9r**. Dual inhibitory activity was observed in compound **9n** (IC_50_=113.31 and 50.75 µM, against STAT3 and STAT5a, respectively). Prediction of physicochemical properties, drug likeness score, pharmacokinetic and toxic properties was detected.

## Graphical Abstract

**Figure:**
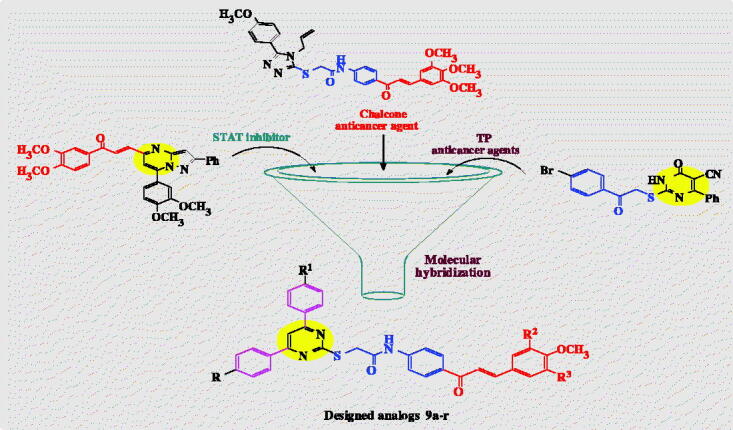

## Introduction

One of the main causes of mortality all over the world is cancer^1^^,^^2^. The highest prevalence for cancer death is being for stomach, breast, prostatic, lung, and colon^3^.

The most common female cancer around the world is breast cancer. It represents for 16% of all female cancers and 18.2% of all cancer death causes including both males and females^4^.

On the other hand, about two million new cases are diagnosed every year for colorectal cancer. Thus, making it as one of the most common causes of cancer-related death^5^^,^^6^.

Another common cause of cancer death is leukaemia, cancer in blood-forming cells of the bone marrow, which is chemoresistant^7–11^. Although, treatment of cancer using chemotherapeutic agents is still used for several cancer types including breast, colon and leukaemia cancers, high toxicity level of chemotherapeutic drugs limit their use^12^.

A critical signalling intermediate in cancer cells, specially leukaemia, breast and colon cancer cells is called signal transducer and activator of transcription (STAT) protein family^13–17^.

They are cytoplasmic transcription factors. STAT family consists of seven members, STAT1, STAT2, STAT3, STAT4, STAT5a, STAT5b, and STAT6. STAT2, STAT4, and STAT6 are responsible for regulation of immune response. While, STAT1, STAT3, and STAT5 can control cell cycle (cyclin D1, D2, c-Myc), cell survival (Bcl-xl, Bcl2, Mcl-1), and angiogenesis (HIF1α, VEGFR) through regulation of gene expression^18^^,^^19^.

STAT can be activated either by receptor tyrosin kinases like JAKs, PDGFR, EGFR, and FLT3, or through non-receptor tyrosin kinases, Src, Brk, and Bcr-Abl. Also, activation of STAT may be from activation of cytokines (IL-6), growth factors or negative feedback mechanisms^20–24^.

Phosphorylation of STATs transforms them to active form causing their homo- or heterodimerisation then migration to the nucleus to control gene expression. Over activation of STAT level can lead to tumorigenesis^20^.

Several studies have demonstrated that blocking STAT3 or STAT5 signalling pathway led to apoptosis in tumour cells. While, normal cells were able to survive even under a very low concentration of STAT3 or STAT5 and also capable of growing using other mechanisms^21^^,^^25^.

Therefore, development of new anti-cancer agents with less toxicity and overcoming chemotherapeutic drug resistance can be achieved by: (1) using drugs that target two or three activators of STAT3^24^ or (2) combined targeting of STAT3 and STAT5^8^.

**Figure 1.**
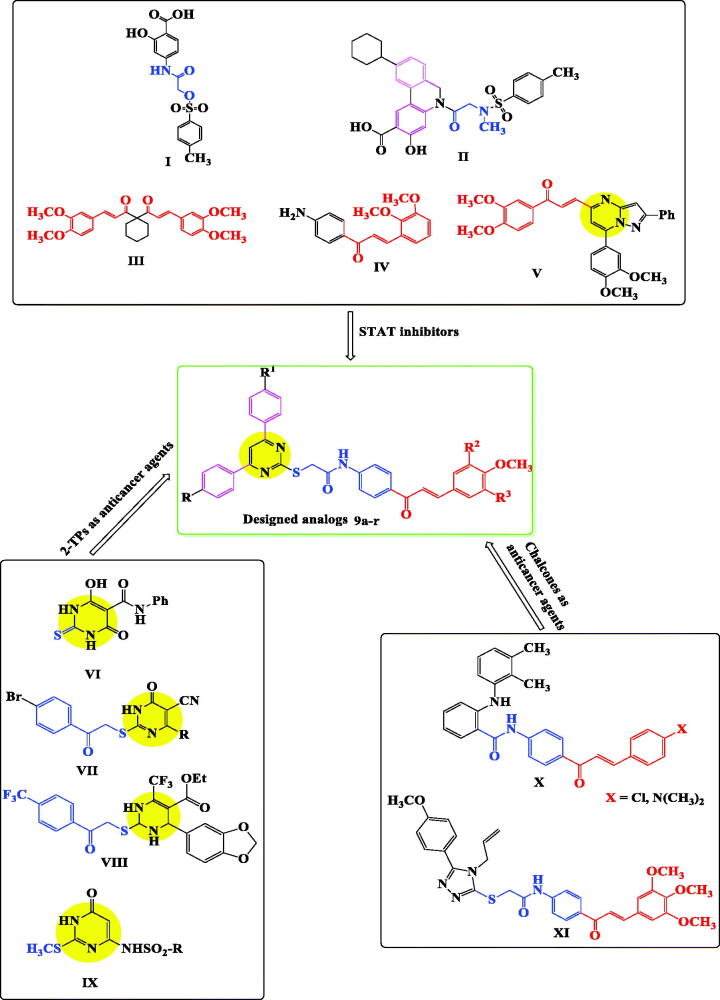
The designed strategy for 2-TP/chalcone hybrids as new anticancer STAT inhibitors.

It was found that a potent STAT3 inhibitor, S31-201 (**I**, Figure 1), could inhibit proliferation of hepatocellular and breast carcinoma in mice^16^.

Moreover, compound S3I-201.1066 (**II**, Figure 1), containing sulphonamide group could inhibit STAT3 function in both breast and myeloma cancer cells (EC_50_=10 and 16 µM, respectively)^26^. Another compound, curcumine analogue, FLLL32 (**III**, Figure 1), showed potent inhibitory activity in many human cancer cell lines such as breast, colorectal, melanoma, and myeloma by preventing STAT3 dimerisation and downstream functioning^20^^,^^27^.

Moreover, treatment with chalcone **IV** (Figure 1) caused significant decrease in STAT3 level in leukaemia HL-60 cell line^28^.

Pyrazolo[1,5-*a*]pyrimidine/chalcone hybrid **V** (Figure 1) showed promising anti-proliferative activity by down regulation of STAT3 in MDA-MB-231 cells^29^.

Additionally, NCI library identified two compounds (**BP-1108** and **BP-1075**) as the most potent STAT5 in K562 leukaemia cell lines through down regulation of STATs-defendant genes^30^.

In medicinal chemistry, a very well-known heterocycle is pyrimidine. It takes its importance from its presence in thymidine, cytosine and uracil bases, the building blocks of DNA and RNA nucleic acids^31^^,^^32^.

2-Thiopyrimidines (2-TPs), also named as 2-mercaptopyrimidines, are one of the most important class of pyrimidines.

They attract the biochemists attention due to their wide range of applications in preparation of cardiotonic drugs, antitubercular and anti-inflammatory agents^33^^,^^34^.

Moreover, 2-TPs were evaluated for their anticancer activity^33^. They were reported to have potent antitumor activity against leukaemia, colon and breast cell lines such as compounds **VI–IX** (Figure 1)^35–38^.

Synthesis of 2-TPs derivatives could be achieved from reaction of chalcone derivatives with thiourea^39^. As chalcones constitute an important group of natural products, their biological activities were arisen from their chemical structure, α,β-unsaturated carbonyl group^40^.

Many synthesised chalcones were reported to have potent *in vitro* anticancer activity against human colon carcinoma, non-small cell lung carcinoma, and breast cancer^41–44^.

Thus, both chloro- and dimethylamino-derivatives of compound **X** (Figure 1), showed cytotoxic activity against human leukaemia cells with CC_50_=2.17 and 2.06 μM, respectively^40^. While, compound **XI** (Figure 1) was apoptosis inducer in A549 cells^45^.

In light of the above facts, and as a part of our previously published anticancer research articles^46^^,^^47^, our scope in this research was to design and synthesised a new series of 2-TP/chalcone hybrids (Figure 1), through molecular hybridisation, by merging:

(i) 2-Thiopyrimidine scaffold, such as in compounds **(VI–IX)**, (ii) chalcone part from compounds (**III**–**V**, **X**, **XI**), (iii) choosing substituents on phenyl rings of pyrimidine C-4, pyrimidine C-6, and chalcone as in compounds (**I–V**), and (iv) amide linkage to mimic that in compounds (**I**, **II**, **VI**, **X**, **XI**). The cytotoxic activities of the synthesised derivatives were evaluated against leukaemia (K-562), breast (MCF-7), and colon (HT-29) cancer cell lines. Inhibitory activities of the most potent bioactive molecules against STAT3 and STAT5a were measured, aiming at finding more effective anticancer therapeutics.

## Experimental

### Chemistry

Melting points were measured on the Griffin apparatus and were uncorrected. Determination of IR spectra was achieved using Shimadzu IR-435 spectrophotometer with KBr discs and values were obtained in cm^−1^. ^1^H NMR and ^13^C NMR were recorded on Bruker instrument at 400 MHz for ^1^H NMR and 100 MHz for ^13^C NMR spectrophotometer (Faculty of Pharmacy, Mansoura University, Mansoura, Egypt), in DMSO-d_6_ (as a solvent), D_2_O using TMS as an internal standard and chemical shifts (*δ*) were expressed in parts per million (ppm) compared to internal standard, TMS (*δ* = 0 ppm). Coupling constant (*J*) values were expressed in Hertz (Hz). Signal splitting patterns were designated as follows: s, singlet; d, doublet, t, triplet; q, quartette; m, multiplet. The electron impact (EI) mass spectra were carried out using Hewlett Packard 5988 spectrometer (Palo Alto, CA) at Faculty of Science, Cairo University, Giza, Egypt. Microanalysis was calculated for C, H, N on Perkin-Elmer 2400 at the Microanalytical centre, Faculty of Science, Cairo University, Egypt and was within ±0.4% of theoretical values. The progress of the reaction and purity of products were monitored by thin layer chromatography (TLC), pre-coated plastic sheets, 0.2 mm silica gel with UV indicator (Macherey-Nagel, Düren, Germany). All used reagents and solvents were purchased from the Aldrich Chemical Company (Milwaukee, WI).

#### General method for preparation of compounds 4a–f

A mixture of the appropriate chalcone derivative **3a–f** (0.01 mol), thiourea (0.76 g, 0.01 mol), and KOH (0.11 g, 0.02 mol) in absolute ethanol (20 ml) was heated under reflux temperature for 12 h. The resulting solution was evaporated to dryness or (a precipitate in case of **3c** and **3f**). The obtained residue was solubilised in water, filtered and dried. The crude product was crystallised from ethanol/DMF (8:2) to get compounds **4a–f**.

##### *4-(4-Methoxyphenyl)-6-*p*-tolylpyrimidine-2-thiol (4a)*

Yield 82%; yellow powder; (ethanol 95%); mp 149–151 °C; IR (cm^−1^): 3413 (NH), 3192 (CH aromatic), 2958 (CH aliphatic); ^1^H NMR (400 MHz, DMSO-d_6_) *δ* 2.50 (s, 3H, CH_3_), 4.43 (s, 3H, OCH_3_), 7.24–7.59 (m, 4H, *p*-methoxyphenyl H-3, H-5, *p*-tolyl H-3, H-5), 7.79–7.91 (m, 4H, pyrimidine H-5, *p*-methoxyphenyl H-2, H-6, NH, D_2_O exchangeable), 8.21 (d, *J*= 8.4 Hz, 2H, *p*-tolyl H-2, H-6); ^13^C NMR (100 MHz, DMSO-d_6_) *δ* 21.2 (CH_3_), 55.6 (OCH_3_), 101.0 (pyrimidine C-5), 114.7 (*p*-methoxyphenyl C-3, C-5), 126.1 (*p*-tolyl C-2, C-6), 128.1 (*p*-tolyl C-3, C-5), 129.4 (*p*-methoxyphenyl C-2, C-6), 130.9 (*p*-methoxyphenyl C-1), 134.5 (*p*-tolyl C-1), 136.7 (*p*-tolyl C-4), 159.2 (*p*-methoxyphenyl C-4), 164.6 (pyrimidine C-4) 172.9 (pyrimidine C-6), 175.2 (pyrimidine C-2); EIMS (*m/z*): 309.00 (M + 1, 31.75%), 308.00 (M^+^, 74.33%), 307.00 (100%); Anal. Calcd. for C_18_H_16_N_2_OS (308.40): C, 70.10; H, 5.23; N, 9.08. Found: C, 70.31; H, 5.07; N, 8.88.

##### 4-(4-Methoxyphenyl)-6-(4-nitrophenyl)pyrimidine-2-thiol (4b)

Yield 75%; yellow crystals; mp 63–65 °C^39^.

##### 4-[4-(2-Chloroethoxy)phenyl]-6-(4-methoxyphenyl)pyrimidine-2-thiol (4c)

Yield 69%; yellow powder; (ethanol 95%); mp 282–284 °C; IR (cm^−1^): 3436 (NH), 3192 (CH aromatic), 2927 (CH aliphatic); ^1^H NMR (400 MHz, DMSO-d_6_) *δ* 3.83–4.37 (m, 7H, OCH_3_, OCH_2_, CH_2_Cl), 6.96–7.10 (m, 7H, *p*-chloroethoxyphenyl H-2, H-3, H-5, H-6, *p*-methoxyphenyl H-3, H-5, NH, D_2_O exchangeable), 8.22–8.30 (m, 3H, *p*-methoxyphenyl H-2, H-6, pyrimidine H-5); ^13^C NMR (100 MHz, DMSO-d_6_) *δ* 42.9 (CH_2_Cl), 55.8 (OCH_3_), 68.1 (OCH_2_), 105.0 (pyrimidine C-5), 114.8 (*p*-methoxyphenyl C-3, C-5), 115.4 (*p*-chloroethoxyphenyl C-3, C-5), 121.8 (*p*-chlorophenyl C-2, C-6), 127.9 (*p*-methoxyphenyl C-1), 128.5 (*p*-methoxyphenyl C-2, C-6), 129.4 (*p*-chloroethoxyphenyl C-2, C-6), 130.0 (*p*-chloroethoxyphenyl C-1), 160.6 (*p*-methoxyphenyl C-4), 161.7 (*p*-chloroethoxyphenyl C-4), 162.5 (pyrimidine C-4) 176.1 (pyrimidine C-6), 179.3 (pyrimidine C-2); Anal. Calcd. for C_19_H_17_ClN_2_O_2_S (372.87): C, 61.20; H, 4.60; N, 7.51. Found: C, 61.41; H, 4.57; N, 7.75.

##### *4-(4-Chlorophenyl)-6-*p*-tolylpyrimidine-2-thiol (4d)*

Yield 91%; yellow crystals; mp 180–182 °C^47^.

##### 4-(4-Chlorophenyl)-6-(4-nitrophenyl)pyrimidine-2-thiol (4e)

Yield 65%; yellow powder; (ethanol 95%); mp 263–265 °C; IR (cm^−1^): 3434 (NH), 3064 (CH aromatic); ^1^H NMR (400 MHz, DMSO-d_6_) *δ* 7.60 (d, *J*= 8.4 Hz, 2H, *p*-chlorophenyl H-3, H-5), 7.96 (s, 1H, NH, D_2_O exchangeable), 8.33–8.34 (m, 4H, *p*-chlorophenyl H-2, H-6, *p*-nitrophenyl H-2, H-6), 8.54 (d, *J*= 8.4 Hz, 2H, *p*-nitrophenyl H-3, H-5), 8.66 (s, 1H, pyrimidine H-5); ^13^C NMR (100 MHz, DMSO-d_6_) *δ* 101.3 (pyrimidine C-5), 123.6 (*p*-chlorophenyl C-2, C-6), 128.0 (*p*-nitrophenyl C-3, C-5), 128.9 (*p*-chlorophenyl C-1), 129.3 (*p*-chlorophenyl C-3, C-5), 129.5 (*p*-nitrophenyl C-2, C-6), 133.5 (*p*-chlorophenyl C-4), 139.2 (*p*-nitrophenyl C-1), 150.2 (*p*-nitrophenyl C-4), 164.6 (pyrimidine C-4) 176.3 (pyrimidine C-6), 180.4 (pyrimidine C-2); Anal. Calcd. for C_16_H_10_ClN_3_O_2_S (343.79): C, 55.90; H, 2.93; N, 12.22. Found: C, 60.12; H, 2.87; N, 12.46.

##### 4-[4-(2-Chloroethoxy)phenyl]-6-(4-chlorophenyl)pyrimidine-2-thiol (4f)

Yield 62%; yellow powder; (ethanol 95%); mp 242–244 °C; IR (cm^−1^): 3417 (NH), 3066 (CH aromatic), 2927 (CH aliphatic); ^1^H NMR (400 MHz, DMSO-d_6_) *δ* 3.98 (t, *J*= 8.4 Hz, 2H, CH_2_Cl), 4.36 (t, *J*= 8.4 Hz, 2H, OCH_2_), 7.07–7.12 (m, 4H, *p*-chloroethoxyphenyl H-3, H-5, *p*-chlorophenyl H-2, H-6), 7.31 (s, 1H, NH, D_2_O exchangeable), 7.60 (d, *J*= 8.4 Hz, 2H, *p*-chlorophenyl H-3, H-5), 7.96 (s, 1H, pyrimidine H-5), 8.31 (d, *J*= 8.4 Hz, 2H, *p*-chloroethoxyphenyl H-2, H-6); ^13^C NMR (100 MHz, DMSO-d_6_) *δ* 43.4 (CH_2_Cl), 68.5 (OCH_2_), 103.9 (pyrimidine C-5), 115.3 (*p*-chloroethoxyphenyl C-3, C-5), 120.2 (*p*-chlorophenyl C-2, C-6), 128.5 (*p*-chlorophenyl C-1), 129.4 (*p*-chloroethoxyphenyl C-2, C-6), 129.5 (*p*-chlorophenyl C-3, C-5), 130.7 (*p*-chloroethoxyphenyl C-1), 136.6 (*p*-chlorophenyl C-4), 161.7 (*p*-chloroethoxyphenyl C-4), 162.8 (pyrimidine C-4) 176.3 (pyrimidine C-6), 179.0 (pyrimidine C-2); EIMS (*m/z*): 376.95 (M + 1, 16.83%), 375.90 (M^+^, 19.31%), 55.10 (100%); Anal. Calcd. for C_18_H_14_ClN_2_OS (377.29): C, 57.30; H, 3.74; N, 7.42. Found: C, 57.41; H, 3.57; N, 7.68.

#### General method for preparation of compounds 9a–r

A mixture of pyrimidine derivatives **4a–f** (0.01 mol), acetyl chloride derivatives **8a–c** (0.01 mol), and catalytic amount of TEA is stirred in acetonitrile (20 ml) for 24 h. The solution was evaporated to dryness. The obtained residue was solubilised in ice cold water and neutralised with conc. HCl. The obtained solid was filtered, dried and crystallised from ethanol/DMF (8:2).

##### *(*ZE*)-2-[4-(4-Methoxyphenyl)-6-*p*-tolylpyrimidin-2-ylthio]-*N*-{4-[3-(4 methoxyphenyl)acryloyl]phenyl}acetamide (9a)*

Yield 82%; yellow powder; mp 226–228 °C; IR (cm^−1^): 3257 (NH), 3039 (CH aromatic), 2925 (CH aliphatic), 1795, 1663 (2C=O); ^1^H NMR (400 MHz, DMSO-d_6_) *δ* 1.86 (s, 3H, CH_3_), 4.41 (s, 3H, OCH_3_), 4.43 (s, 3H, OCH_3_), 4.84 (s, 2H, CH_2_), 6.67 (d, *J*= 8.4 Hz, 2H, *p*-methoxyphenylacroyl H-3, H-5), 7.10 (d, *J*= 7.6 Hz, 2H, *p*-methoxyphenyl H-3, H-5), 7.24 (d, *J*= 8.4 Hz, 2H, *p*-methoxyphenylacroyl H-2, H-6), 7.33 (d, *J*= 6.0 Hz, 2H, *p*-tolyl H-3, H-5), 7.44–7.74 (m, 7H, COC*H*=C*H*, pyrimidine H-5, *p*-methoxyphenyl H-2, H-6, *p*-tolyl H-2, H-6), 7.91–7.94 (m, 4H, phenyl H-2, H-3, H-5, H-6), 10.84 (s, 1H, NH, D_2_O exchangeable); ^13^C NMR (100 MHz, DMSO-d_6_) *δ* 22.3 (CH_3_), 36.2 (CH_2_), 56.4 (2OCH_3_), 113.7 (*p*-methoxyphenylacroyl C-3, C-5), 118.8 (*p*-methoxyphenyl C-3, C-5), 120.1 (CO*C*H=CH), 121.7 (phenyl C-2, C-6), 127.5 (*p*-methoxyphenylacroyl C-1), 127.6 (*p*-tolyl C-2, C-6), 129.0 (*p*-methoxyphenyl C-2, C-6), 129.1 (*p*-methoxyphenyl C-1), 130.2 (*p*-tolyl C-3, C-5), 130.8 (*p*-methoxyphenylacroyl C-2, C-6), 131.4 (phenyl C-3, C-5), 132.0 (*p*-tolyl C-4), 133.5 (phenyl C-4), 136.9 (*p*-tolyl C-1), 144.3 (phenyl C-1), 154.4 (COCH=*C*H), 155.1 (*p*-methoxyphenylacroyl C-4), 155.4 (*p*-methoxyphenyl C-4), 164.1 (pyrimidine C-6), 167.8 (pyrimidine C-4), 172.3 (pyrimidine C-2), 173.0 (CONH), 190.0 (CO); Anal. Calcd. for C_36_H_31_N_3_O_4_S (601.20): C, 71.86; H, 5.19; N, 6.98. Found: C, 71.67; H, 5.07; N, 6.93.

##### *(*ZE*)-*N*-{4-[3-(3,4-Dimethoxyphenyl)acryloyl]phenyl}-2-[4-(4-methoxyphenyl)-6-*p*-tolylpyrimidin-2-ylthio]acetamide (9b)*

Yield 65%; yellow powder; mp 165–167 °C; IR (cm^−1^): 3324 (NH), 3061 (CH aromatic), 2925 (CH aliphatic), 1663 (broad, 2C=O); ^1^H NMR (400 MHz, DMSO-d_6_) *δ* 2.36 (s, 3H, CH_3_), 3.81 (s, 3H, OCH_3_), 3.82 (s, 3H, OCH_3_), 3.86 (s, 3H, OCH_3_), 4.23 (s, 2H, CH_2_), 6.97 (d, *J*= 8.4 Hz, 2H, *p*-methoxyphenyl H-3, H-5), 7.03 (d, *J*= 8.0 Hz, 1H, dimethoxyphenyl H-5), 7.28 (d, *J*= 8.0 Hz, 1H, dimethoxyphenyl H-6), 7.37 (d, *J*= 12.0 Hz, 1H, COC*H*=CH), 7.54 (s, 1H, dimethoxyphenyl H-2), 7.69 (d, *J*= 12.0 Hz, 1H, COCH=C*H*), 7.83–7.87 (m, 3H, *p*-tolyl H-3, H-5, pyridine H-5), 8.18–8.23 (m, 6H, phenyl H-2, H-6, *p*-methoxyphenyl H-2, H-6, *p*-tolyl H-2, H-6), 8.31 (d, *J*= 8.4 Hz, 2H, phenyl H-3, H-5), 10.84 (s, 1H, NH, D_2_O exchangeable); ^13^C NMR (100 MHz, DMSO-d_6_) *δ* 21.4 (CH_3_), 36.4 (CH_2_), 55.8 (OCH_3_), 56.0 (OCH_3_), 56.1 (OCH_3_), 107.4 (pyrimidine C-5), 111.0 (dimethoxyphenyl C-2), 111.9 (dimethoxyphenyl C-5), 114.6 (dimethoxyphenyl C-6), 118.8 (*p*-methoxyphenyl C-3, C-5), 119.8 (CO*C*H=CH), 124.4 (phenyl C-2, C-6), 127.7 (*p*-tolyl C-2, C-6), 128.0 (*p*-methoxyphenyl C-2, C-6), 129.6 (*p*-methoxyphenyl C-1), 130.3 (*p*-tolyl C-3, C-5), 130.4 (*p*-tolyl C-1), 133.0 (dimethoxyphenyl C-1), 133.6 (phenyl C-3, C-5), 141.7 (*p*-tolyl C-4), 143.9 (phenyl C-4), 144.4 (phenyl C-1), 149.4 (dimethoxyphenyl C-3), 150.0 (dimethoxyphenyl C-4), 151.6 (COCH=*C*H), 162.3 (*p*-methoxyphenyl C-4), 164.2 (pyrimidine C-6), 164.3 (pyrimidine C-4), 167.7 (pyrimidine C-2), 170.8 (CONH), 187.8 (CO); Anal. Calcd. for C_37_H_33_N_3_O_5_S (631.21): C, 70.34; H, 5.27; N, 6.65. Found: C, 70.54; H, 5.07; N, 6.71.

##### *(*ZE*)-2-[4-(4-Methoxyphenyl)-6-*p*-tolylpyrimidin-2-ylthio]-*N*-{4-[3-(3,4,5-trimethoxyphenyl)acryloyl]phenyl}acetamide (9c)*

Yield 56%; yellow powder; mp 195–197 °C; IR (cm^−1^): 3290 (NH), 2998 (CH aromatic), 2927 (CH aliphatic), 1665 (broad, 2C=O); ^1^H NMR (400 MHz, DMSO-d_6_) *δ* 2.36 (s, 3H, CH_3_), 3.72 (s, 3H, OCH_3_), 3.81 (s, 3H, OCH_3_), 3.87 (s, 6H, 2OCH_3_), 4.24 (s, 2H, CH_2_), 6.95 (s, 2H, trimethoxyphenyl H-2, H-6), 6.98–7.28 (m, 4H, *p*-methoxyphenyl H-3, H-5, *p*-tolyl H-3, H-5), 7.69 (d, *J*= 12.0 Hz, 1H, COC*H*=CH), 7.87–8.19 (m, 3H, COCH=C*H*, *p*-tolyl H-2, H-6), 8.22–8.31 (m, 7H, *p*-methoxyphenyl H-2, H-6, pyrimidine H-5, phenyl H-2, H-3, H-5, H-6), 10.86 (s, 1H, NH, D_2_O exchangeable); ^13^C NMR (100 MHz, DMSO-d_6_) *δ* 21.4 (CH_3_), 36.4 (CH_2_), 55.8 (OCH_3_), 56.5 (2OCH_3_), 60.6 (OCH_3_), 106.8 (trimethoxyphenyl C-2, C-6), 107.4 (pyrimidine C-5), 114.5 (*p*-methoxyphenyl C-3, C-5), 118.8 (phenyl C-2, C-6), 121.5 (CO*C*H=CH), 126.9 (*p*-tolyl C-2, C-6), 127.7 (trimethoxyphenyl C-1), 128.5 (*p*-methoxyphenyl C-1), 129.6 (*p*-methoxyphenyl C-2, C-6), 130.4 (*p*-tolyl C-3, C-5), 131.0 (*p*-tolyl C-4), 132.8 (phenyl C-3, C-5), 133.5 (*p*-tolyl C-1), 140.0 (phenyl C-4), 141.7 (trimethoxyphenyl C-4), 144.0 (phenyl C-1), 144.4 (COCH=*C*H), 153.5 (trimethoxyphenyl C-3, C-5), 162.3 (*p*-methoxyphenyl C-4), 164.2 (pyrimidine C-6), 164.3 (pyrimidine C-4), 167.7 (pyrimidine C-2), 170.7 (CONH), 187.9 (CO); EIMS (*m/z*): 662.05 (M + 1, 5.50%), 661.05 (M^+^, 12.94%), 322.05 (100%); Anal. Calcd. for C_38_H_35_N_3_O_6_S (661.22): C, 68.97; H, 5.33; N, 6.35. Found: C, 68.78; H, 5.17; N, 6.24.

##### *(*ZE*)-2-[4-(4-Methoxyphenyl)-6-(4-nitrophenyl)pyrimidin-2-ylthio]-*N*-{4-[3-(4-methoxyphenyl)acryloyl]phenyl}acetamide (9d)*

Yield 57%; yellow powder; mp 183–185 °C; IR (cm^−1^): 3407 (NH), 3063 (CH aromatic), 2930 (CH aliphatic), 1664 (broad, 2C=O); ^1^H NMR (400 MHz, DMSO-d_6_) *δ* 3.83 (s, 3H, OCH_3_), 3.84 (s, 3H, OCH_3_), 4.24 (s, 2H, CH_2_), 6.98 (d, *J*= 8.8 Hz, 2H, *p*-methoxyphenylacryloyl H-3, H-5), 7.03 (d, *J*= 8.8 Hz, 2H, *p*-methoxyphenyl H-3, H-5), 7.69 (d, *J*= 15.6 Hz, 1H, COC*H*=CH), 7.80–7.86 (m, 5H, COCH=C*H*, *p*-methoxyphenylacryloyl H-2, H-6, *p*-methoxyphenyl H-2, H-6), 8.18 (d, *J*= 8.8 Hz, 2H, phenyl H-2, H-6), 8.27 (d, *J*= 8.8 Hz, 2H, phenyl H-3, H-5), 8.33 (d, *J*= 8.8 Hz, 2H, *p*-nitrophenyl H-2, H-6), 8.40 (s, 1H, pyrimidine H-5), 8.56 (d, *J*= 8.8 Hz, 2H, *p*-nitrophenyl H-3, H-5), 10.86 (s, 1H, NH, D_2_O exchangeable); ^13^C NMR (100 MHz, DMSO-d_6_) *δ* 36.4 (CH_2_), 55.8 (OCH_3_), 55.9 (OCH_3_), 109.1 (pyrimidine C-5), 114.7 (*p*-methoxyphenylacryloyl C-3, C-5), 114.8 (*p*-methoxyphenyl C-3, C-5), 118.8 (phenyl C-2, C-6), 119.8 (*p*-nitrophenyl C-3, C-5), 124.2 (CO*C*H=CH), 127.8 (*p*-nitrophenyl C-2, C-6), 128.2 (*p*-methoxyphenyl C-2, C-6), 129.1 (*p*-methoxyphenylacryloyl C-1), 129.8 (*p*-methoxyphenyl C-1), 130.0 (*p*-methoxyphenylacryloyl C-2, C-6), 130.3 (phenyl C-3, C-5), 131.1 (*p*-nitrophenyl C-1), 133.1 (phenyl C-4), 142.4 (phenyl C-1), 143.8 (COCH=*C*H), 149.3 (*p*-nitrophenyl C-4), 161.7 (*p*-methoxyphenylacryloyl C-4), 162.2 (*p*-methoxyphenyl C-4), 162.6 (pyrimidine C-6), 164.9 (pyrimidine C-4), 167.6 (pyrimidine C-2), 171.3 (CONH), 187.8 (CO); Anal. Calcd. for C_35_H_28_N_4_O_6_S (632.17): C, 66.44; H, 4.46; N, 8.86. Found: C, 66.42; H, 4.39; N, 8.74.

##### *(*ZE*)-*N*-{4-[3-(3,4-Dimethoxyphenyl)acryloyl]phenyl}-2-[4-(4-methoxyphenyl)-6-(4-nitrophenyl)pyrimidin-2-ylthio]-acetamide (9e)*

Yield 54%; yellow powder; mp 225–227 °C; IR (cm^−1^): 3431 (NH), 3039 (CH aromatic), 2924 (CH aliphatic), 1656 (broad, 2C=O); ^1^H NMR (400 MHz, DMSO-d_6_) *δ* 3.83 (s, 3H, OCH_3_), 3.87 (s, 6H, 2OCH_3_), 4.27 (s, 2H, CH_2_), 6.99 (d, *J*= 8.4 Hz, 2H, *p*-methoxyphenyl H-3, H-5), 7.03 (d, *J*= 8.8 Hz, 1H, dimethoxyphenyl H-5), 7.39 (d, *J*= 8.8 Hz, 1H, dimethoxyphenyl H-6), 7.55 (s, 1H, dimethoxyphenyl H-2), 7.68 (d, *J*= 15.2 Hz, 1H, COC*H*=CH), 7.82–7.88 (m, 3H, COCH=C*H*, *p*-methoxyphenyl H-2, H-6), 8.20 (d, *J*= 8.8 Hz, 2H, phenyl H-2, H-6), 8.28 (d, *J*= 8.8 Hz, 2H, phenyl H-3, H-5), 8.34 (d, *J*= 8.8 Hz, 2H, *p*-nitrophenyl H-2, H-6), 8.41 (s, 1H, pyrimidine H-5), 8.57 (d, *J*= 8.8 Hz, 2H, *p*-nitrophenyl H-3, H-5), 10.87 (s, 1H, NH, D_2_O exchangeable); ^13^C NMR (100 MHz, DMSO-d_6_) *δ* 36.4 (CH_2_), 55.9 (OCH_3_), 56.0 (OCH_3_), 56.2 (OCH_3_), 109.1 (pyrimidine C-5), 111.0 (dimethoxyphenyl C-2), 112.0 (dimethoxyphenyl C-5), 114.7 (*p*-methoxyphenyl C-3, C-5), 118.8 (dimethoxyphenyl C-6), 119.8 (phenyl C-2, C-6), 124.2 (*p*-nitrophenyl C-3, C-5), 124.4 (CO*C*H=CH), 128.0 (*p*-nitrophenyl C-2, C-6), 128.2 (*p*-methoxyphenyl C-2, C-6), 129.1 (dimethoxyphenyl C-1), 129.8 (*p*-methoxyphenyl C-1), 130.3 (phenyl C-3, C-5), 132.0 (phenyl C-4), 142.4 (phenyl C-1), 143.8 (*p*-nitrophenyl C-1), 144.4 (COCH=*C*H), 149.4 (dimethoxyphenyl C-4), 149.4 (dimethoxyphenyl C-3), 153.5 (*p*-nitrophenyl C-4), 162.2 (*p*-methoxyphenyl C-4), 162.6 (pyrimidine C-6), 165.0 (pyrimidine C-4), 167.6 (pyrimidine C-2), 171.3 (CONH), 187.8 (CO); Anal. Calcd. for C_36_H_30_N_4_O_7_S (662.18): C, 65.24; H, 4.56; N, 8.45. Found: C, 65.35; H, 4.71; N, 8.24.

##### *(*ZE*)-2-[4-(4-Methoxyphenyl)-6-(4-nitrophenyl)pyrimidin-2-ylthio]-*N*-{4-[3-(3,4,5-trimethoxyphenyl)acryloyl]phenyl}acetamide (9f)*

Yield 69%; yellow powder; mp 254–256 °C; IR (cm^−1^): 3265 (NH), 3103 (CH aromatic), 2933 (CH aliphatic), 1663 (broad, 2C=O); ^1^H NMR (400 MHz, DMSO-d_6_) *δ* 3.71 (s, 3H, OCH_3_), 3.82 (s, 3H, OCH_3_), 3.87 (s, 6H, 2OCH_3_), 4.27 (s, 2H, CH_2_), 6.98 (d, *J*= 8.0 Hz, 2H, *p*-methoxyphenyl H-3, H-5), 7.23 (s, 2H, trimethoxyphenyl H-2, H-6), 7.69 (d, *J*= 12.0 Hz, 1H, COC*H*=CH), 7.86–7.92 (m, 3H, COCH=C*H*, *p*-methoxyphenyl H-2, H-6), 8.21 (d, *J*= 8.0 Hz, 2H, phenyl H-2, H-6), 8.27 (d, *J*= 8.0 Hz, 2H, phenyl H-3, H-5), 8.33 (d, *J*= 8.0 Hz, 2H, *p-*nitrophenyl H-2, H-6), 8.40 (s, 1H, pyrimidin H-5), 8.56 (d, *J*= 8.0 Hz, 2H, *p-*nitrophenyl H-3, H-5), 10.89 (s, 1H, NH, D_2_O exchangeable); ^13^C NMR (100 MHz, DMSO-d_6_) *δ* 36.4 (CH_2_), 55.8 (OCH_3_), 56.5 (2OCH_3_), 60.6 (OCH_3_), 106.8 (trimethoxyphenyl C-2, C-6), 109.0 (pyrimidine C-5), 114.6 (*p*-methoxyphenyl C-3, C-5), 118.8 (phenyl C-2, C-6), 121.4 (CO*C*H=CH), 124.2 (*p*-nitrophenyl C-3, C-5), 128.1 (trimethoxyphenyl C-1), 129.1 (*p*-nitrophenyl C-2, C-6), 129.8 (*p*-methoxyphenyl C-2, C-6), 130.4 (phenyl C-3, C-5), 130.7 (*p*-methoxyphenyl C-1), 132.9 (phenyl C-4), 140.0 (trimethoxyphenyl C-4), 142.3 (*p*-nitrophenyl C-1), 144.3 (phenyl C-1), 144.4 (COCH=*C*H), 149.3 (*p*-nitrophenyl C-4), 153.5 (trimethoxyphenyl C-3, C-5), 162.2 (*p*-methoxyphenyl C-4), 162.6 (pyrimidine C-6), 164.9 (pyrimidine C-4), 167.6 (pyrimidine C-2), 171.3 (CONH), 187.9 (CO); EIMS (*m/z*): 693.00 (M + 1, 0.93%), 692.00 (M^+^, 1.33%), 55.10 (100%); Anal. Calcd. for C_37_H_32_N_4_O_8_S (692.19): C, 64.15; H, 4.66; N, 8.09. Found: C, 63.98; H, 4.57; N, 7.89.

##### *(*ZE*)-2-{4-[4-(2-Chloroethoxy)phenyl]-6-(4-methoxyphenyl)pyrimidin-2-ylthio}-*N*-{4-[3-(4-methoxyphenyl)acryloyl]phenyl}acetamide (9g)*

Yield 52%; yellow powder; mp 280–282 °C; IR (cm^−1^): 3431 (NH), 3039 (CH aromatic), 2935 (CH aliphatic), 1598 (broad, 2C=O); ^1^H NMR (400 MHz, DMSO-d_6_) *δ* 3.70 (s, 3H, OCH_3_), 3.83–3.85 (m, 5H, OCH_3_ and CH_2_Cl), 4.21–4.23 (m, 4H, OCH_2_ and CH_2_), 7.02–7.16 (m, 6H, *p*-methoxyphenyl H-3, H-5, *p*-methoxyphenylacryloyl H-3, H-5 and *p*-chloroethoxyphenyl H-3, H-5), 7.71–7.96 (m, 9H, *p*-methoxyphenyl H-2, H-6, *p*-methoxyphenylacryloyl H-2, H-6, *p*-chloroethoxyphenyl H-2, H-6, phenyl H-2, H-6 and COC*H*=CH), 8.16 (d, *J*= 8.4 Hz, 2H, phenyl H-3, H-5), 8.18–8.20 (m, 2H, pyrimidin H-5 and COCH=C*H*), 10.89 (s, 1H, NH, D_2_O exchangeable); ^13^C NMR (100 MHz, DMSO-d_6_) *δ* 36.2 (CH_2_), 40.6 (CH_2_Cl), 55.4 (OCH_3_), 55.8 (OCH_3_), 68.9 (OCH_2_), 107.8 (pyrimidine C-5), 114.1 (*p*-methoxyphenylacroyl C-3, C-5), 114.7 (*p*-methoxyphenyl C-3, C-5), 114.8 (*p*-chloroethoxyphenyl C-3, C-5), 121.3 (CO*C*H=CH), 122.1 (phenyl C-2, C-6), 127.4 (*p*-chloroethoxyphenyl C-1), 127.5 (*p*-methoxyphenylacroyl C-1), 128.3 (*p*-chloroethoxyphenyl C-2, C-6), 129.5 (*p*-methoxyphenyl C-2, C-6), 130.3 (*p*-methoxyphenylacroyl C-2, C-6), 131.1 (phenyl C-3, C-5), 133.5 (phenyl C-4), 144.0 (phenyl C-1), 144.3 (*p*-methoxyphenyl C-1), 145.4 (COCH=*C*H), 159.3 (*p*-chloroethoxyphenyl C-4), 159.8 (*p*-methoxyphenylacroyl C-4), 160.6 (*p*-methoxyphenyl C-4), 162.8 (pyrimidine C-6), 164.9 (pyrimidine C-4), 168.6 (pyrimidine C-2), 172.3 (CONH), 189.9 (CO); Anal. Calcd. for C_37_H_32_ClN_3_O_5_S (665.18): C, 66.71; H, 4.84; N, 6.31. Found: C, 66.98; H, 4.57; N, 6.28.

##### *(*ZE*)-2-{4-[4-(2-Chloroethoxy)phenyl]-6-(4-methoxyphenyl)pyrimidin-2-ylthio}-*N*-{4-[3-(3,4-dimethoxyphenyl)acryloyl]phenyl}acetamide (9h)*

Yield 52%; yellow powder; mp 135–137 °C; IR (cm^−1^): 3426 (NH), 3067 (CH aromatic), 2928 (CH aliphatic), 1657 (broad, 2C=O); ^1^H NMR (400 MHz, DMSO-d_6_) *δ* 3.70 (s, 3H, OCH_3_), 3.82 (s, 3H, OCH_3_), 3.86 (s, 3H, OCH_3_), 3.96 (t, *J*= 7.2 Hz, 2H, CH_2_Cl), 4.23 (s, 2H, CH_2_), 4.34 (t, *J*= 7.2 Hz, 2H, OCH_2_), 6.82–7.12 (m, 7H, *p*-methoxyphenyl H-3, H-5, *p*-chloroethoxyphenyl H-3, H-5, dimethoxyphenyl H-2, H-5, H-6), 7.38 (d, *J*= 15.2 Hz, 1H, COC*H*=CH), 7.67–7.86 (m, 5H, COCH=C*H*, *p*-methoxyphenyl H-2, H-6, *p*-chloroethoxyphenyl H-2, H-6), 8.18–8.29 (m, 5H, phenyl H-2, H-6, phenyl H-3, H-5, pyrimidin H-5), 10.89 (s, 1H, NH, D_2_O exchangeable); ^13^C NMR (100 MHz, DMSO-d_6_) *δ* 42.2 (CH_2_), 43.4 (CH_2_Cl), 55.8 (OCH_3_), 56.1 (OCH_3_), 56.2 (OCH_3_), 75.1 (OCH_2_), 109.0 (pyrimidine C-5), 111.5 (dimethoxyphenyl C-2), 111.9 (dimethoxyphenyl C-5), 114.5 (*p*-methoxyphenyl C-3, C-5), 115.1 (*p*-chloroethoxyphenyl C-3, C-5), 118.8 (phenyl C-2, C-6), 119.8 (CO*C*H=CH), 124.4 (dimethoxyphenyl C-6), 127.3 (dimethoxyphenyl C-1), 127.4 (*p*-chloroethoxyphenyl C-1), 128.0 (*p*-chloroethoxyphenyl C-2, C-6), 128.7 (*p*-methoxyphenyl C-1), 129.6 (*p*-methoxyphenyl C-2, C-6), 130.3 (phenyl C-3, C-5), 131.3 (phenyl C-4), 143.9 (phenyl C-1), 144.4 (COCH=*C*H), 149.4 (dimethoxyphenyl C-4), 151.6 (dimethoxyphenyl C-3), 157.2 (*p*-chloroethoxyphenyl C-4), 161.2 (*p*-methoxyphenyl C-4), 162.2 (pyrimidine C-6), 164.3 (pyrimidine C-4), 167.6 (pyrimidine C-2), 170.7 (CONH), 187.6 (CO); Anal. Calcd. for C_38_H_34_ClN_3_O_6_S (695.19): C, 65.56; H, 4.92; N, 6.04. Found: C, 65.74; H, 5.07; N, 5.99.

##### *(*ZE*)-2-{4-[4-(2-Chloroethoxy)phenyl]-6-(4-methoxyphenyl)pyrimidin-2-ylthio}-*N*-{4-[3-(3,4,5-trimethoxyphenyl)acryloyl]phenyl}acetamide (9i)*

Yield 54%; yellow powder; mp 116–118 °C; IR (cm^−1^): 3417 (NH), 3039 (CH aromatic), 2934 (CH aliphatic), 1658 (broad, 2C=O); ^1^H NMR (400 MHz, DMSO-d_6_) *δ* 3.71 (s, 3H, OCH_3_), 3.72 (s, 3H, OCH_3_), 3.82 (t, *J*= 3.6 Hz, 2H, CH_2_Cl), 3.88 (s, 6H, 2OCH_3_), 4.23 (s, 2H, CH_2_), 4.34 (t, *J*= 3.6 Hz, 2H, OCH_2_,), 7.25 (s, 2H, trimethoxyphenyl H-2, H-6), 7.68–7.72 (m, 3H, *p*-chloroethoxyphenyl H-3, H-5 and COC*H*=CH), 7.81 (d, *J*= 8.8 Hz, 2H, *p*-methoxyphenyl H-3, H-5), 7.90 (d, *J*= 8.8 Hz, 2H, phenyl H-3, H-5), 7.95 (d, *J*= 8.2 Hz, *p*-chloroethoxyphenyl H-2, H-6) , 8.20–8.22 (m, 4H, phenyl H-2, H-6 and *p*-methoxyphenyl H-2, H-6), 8.29–8.31 (m, 2H, COCH=C*H* and pyrimidin H-5), 10.71 (s, 1H, NH, D_2_O exchangeable); ^13^C NMR (100 MHz, DMSO-d_6_) *δ* 43.4 (CH_2_), 55.4 (CH_2_Cl), 55.8 (OCH_3_), 56.5 (2OCH_3_), 60.6 (OCH_3_), 68.6 (OCH_2_), 106.5 (trimethoxyphenyl C-2, C-6), 106.9 (pyrimidine C-5), 114.1 (*p*-methoxyphenyl C-3, C-5), 114.9 (*p*-chloroethoxyphenyl C-3, C-5), 118.9 (phenyl C-2, C-6), 119.8 (CO*C*H=CH), 121.5 (trimethoxyphenyl C-1), 129.6 (*p*-chloroethoxyphenyl C-1), 129.7 (*p*-chloroethoxyphenyl C-2, C-6), 130.4 (phenyl C-3, C-5), 130.7 (*p*-methoxyphenyl C-1), 131.0 (*p*-methoxyphenyl C-2, C-6), 131.6 (phenyl C-4), 140.0 (trimethoxyphenyl C-4), 143.7 (phenyl C-1), 144.4 (COCH=*C*H), 153.5 (trimethoxyphenyl C-3, C-5), 160.6 (*p*-methoxyphenyl C-4), 161.7 (*p*-chloroethoxyphenyl C-4), 164.1 (pyrimidine C-6), 164.0 (pyrimidine C-4), 168.6 (pyrimidine C-2), 172.3 (CONH), 187.9 (CO); Anal. Calcd. for C_39_H_36_ClN_3_O_7_S (725.20): C, 64.50; H, 5.00; N, 5.79. Found: C, 64.38; H, 4.98; N, 5.53.

##### *(*ZE*)-2-[4-(4-Chlorophenyl)-6-*p*-tolylpyrimidin-2-ylthio]-*N*-{4-[3-(4-methoxyphenyl)acryloyl]phenyl}acetamide (9j)*

Yield 59%; yellow powder; mp 242–244 °C; IR (cm^−1^): 3256 (NH), 3038 (CH aromatic), 2917 (CH aliphatic), 1663 (broad, 2C=O); ^1^H NMR (400 MHz, DMSO-d_6_) *δ* 2.36 (s, 3H, CH_3_), 3.82 (s, 3H, OCH_3_), 4.24 (s, 2H, CH_2_), 7.02 (d, *J*= 8.8 Hz, 2H, methoxyphenyl H-3, H-5), 7.27 (d, *J*= 8.0 Hz, 2H, *p*-tolyl H-3, H-5), 7.52 (d, *J*= 8.8 Hz, 2H, *p*-chlorophenyl H-3, H-5), 7.69 (d, *J*= 11.6 Hz, 1H, COC*H*=CH), 7.82 (d, *J*= 11.6 Hz, 1H, COCH=C*H*), 7.83–7.85 (m, 4H, *p*-tolyl H-2, H-6, *p*-methoxyphenyl H-2, H-6), 8.16 (d, *J*= 8.8 Hz, 2H, phenyl H-2, H-6), 8.22 (d, *J*= 8.8 Hz, 2H, phenyl H-3, H-5), 8.30 (s, 1H, pyrimidine H-5), 8.34 (d, *J*= 8.8 Hz, 2H, *p*-chlorophenyl H-2, H-6), 10.84 (s, 1H, NH, D_2_O exchangeable); ^13^C NMR (100 MHz, DMSO-d_6_) *δ* 21.4 (CH_3_), 36.4 (CH_2_), 55.8 (OCH_3_), 108.3 (pyrimidine C-5), 114.8 (*p*-methoxyphenyl C-3, C-5), 118.8 (phenyl C-2, C-6), 119.8 (CO*C*H=CH), 127.8 (*p*-tolyl C-2, C-6), 129.3 (*p*-chlorophenyl C-2, C-6), 129.6 (*p*-chlorophenyl C-3, C-5), 129.9 (*p*-tolyl C-3, C-5), 130.3 (*p*-methoxyphenyl C-2, C-6), 131.1 (phenyl C-3, C-5), 133.0 (*p*-methoxyphenyl C-1), 133.3 (*p*-tolyl C-4), 135.1 (*p*-tolyl C-1), 136.6 (phenyl C-4), 142.0 (*p*-chlorophenyl C-1), 143.8 (phenyl C-1), 143.8 (*p*-chlorophenyl C-4), 148.0 (COCH=*C*H), 161.7 (*p*-methoxyphenyl C-4), 163.4 (pyrimidine C-6), 164.9 (pyrimidine C-4), 167.6 (pyrimidine C-2), 171.1 (CONH), 187.8 (CO); EIMS (*m/z*): 607.20 (M + 2, 1.54%), 606.15 (M + 1, 1.28%), 605.15 (M^+^, 2.65%), 57.10 (100%); Anal. Calcd. for C_35_H_28_ClN_3_O_3_S (605.15): C, 69.35; H, 4.66; N, 6.93. Found: C, 69.41; H, 4.87; N, 7.13.

##### (ZE*)-2-[4-(4-Chlorophenyl)-6-*p*-tolylpyrimidin-2-ylthio]-*N*-{4-[3-(3,4-dimethoxyphenyl)acryloyl]phenyl}acetamide (9k)*

Yield 57%; yellow powder; mp 144–146 °C; IR (cm^−1^): 3273 (NH), 3039 (CH aromatic), 2924 (CH aliphatic), 1665 (broad, 2C=O); ^1^H NMR (400 MHz, DMSO-d_6_) *δ* 2.35 (s, 3H, CH_3_), 3.85 (s, 3H, OCH_3_), 3.86 (s, 3H, OCH_3_), 4.24 (s, 2H, CH_2_), 7.01 (d, *J*= 8.4 Hz, 1H, dimethoxyphenyl H-5), 7.27 (d, *J*= 8.0 Hz, 2H, *p*-tolyl H-3, H-5), 7.37 (d, *J*= 8.4 Hz, 1H, dimethoxyphenyl H-6), 7.50 (d, *J*= 8.8 Hz, 2H, *p*-chlorophenyl H-3, H-5), 7.53 (s, 1H, dimethoxyphenyl H-2), 7.69 (d, *J*= 11.6 Hz, 1H, COC*H*=CH), 7.81 (d, *J*= 11.6 Hz, 1H, COCH=C*H*), 7.85 (d, *J*= 8.0 Hz, 2H, *p*-tolyl H-2, H-6), 8.18 (d, *J*= 8.0 Hz, 2H, phenyl H-2, H-6), 8.22 (d, *J*= 8.0 Hz, 2H, phenyl H-3, H-5), 8.28 (s, 1H, pyrimidine H-5), 8.34 (d, *J*= 8.8 Hz, 2H, *p*-chlorophenyl H-2, H-6), 10.91 (s, 1H, NH, D_2_O exchangeable); ^13^C NMR (100 MHz, DMSO-d_6_) *δ* 21.4 (CH_3_), 36.2 (CH_2_), 56.1 (OCH_3_), 56.8 (OCH_3_), 108.3 (pyrimidine C-5), 111.0 (dimethoxyphenyl C-2), 111.9 (dimethoxyphenyl C-5), 118.8 (phenyl C-2, C-6), 119.6 (dimethoxyphenyl C-6), 119.8 (*p*-tolyl C-2, C-6), 124.3 (CO*C*H=CH), 127.8 (*p*-chlorophenyl C-2, C-6), 128.0 (dimethoxyphenyl C-1), 129.3 (*p*-chlorophenyl C-3, C-5), 129.6 (*p*-tolyl C-3, C-5), 129.9 (phenyl C-3, C-5), 130.3 (*p*-tolyl C-4), 133.0 (*p*-tolyl C-1), 133.3 (*p*-chlorophenyl C-1), 134.0 (*p*-chlorophenyl C-4), 135.1 (phenyl C-4), 136.6 (phenyl C-1), 144.7 (COCH=*C*H), 149.4 (dimethoxyphenyl C-4), 151.6 (dimethoxyphenyl C-3), 163.4 (pyrimidine C-6), 164.9 (pyrimidine C-4), 167.6 (pyrimidine C-2), 171.1 (CONH), 187.9 (CO); Anal. Calcd. for C_36_H_30_ClN_3_O_4_S (635.16): C, 67.97; H, 4.75; N, 6.61. Found: C, 67.85; H, 4.58; N, 6.42.

##### (ZE*)-2-[4-(4-Chlorophenyl)-6-*p*-tolylpyrimidin-2-ylthio]-*N*-{4-[3-(3,4,5-trimethoxyphenyl)acryloyl]phenyl}acetamide (9l)*

Yield 62%; yellow powder; mp 234–236 °C; IR (cm^−1^): 3280 (NH), 3088 (CH aromatic), 2927 (CH aliphatic), 1663 (broad, 2C=O); ^1^H NMR (400 MHz, DMSO-d_6_) *δ* 2.36 (s, 3H, CH_3_), 3.71 (s, 3H, OCH_3_), 3.86 (s, 6H, 2OCH_3_), 4.25 (s, 2H, CH_2_), 7.23 (s, 2H, trimethoxyphenyl H-2, H-6), 7.28 (d, *J*= 8.0 Hz, 2H, *p*-tolyl H-3, H-5), 7.52 (d, *J*= 8.4 Hz, 2H, *p*-chlorophenyl H-3, H-5), 7.69 (d, *J*= 12.8 Hz, 1H, COC*H*=CH), 7.87 (d, *J*= 8.0 Hz, 2H, *p*-tolyl H-2, H-6), 7.93 (d, *J*= 12.8 Hz, 1H, COCH=C*H*), 8.19–8.24 (m, 4H, phenyl H-2, H-3, H-5, H-6), 8.31 (s, 1H, pyrimidine H-5), 8.35 (d, *J*= 8.4 Hz, 2H, *p*-chlorophenyl H-2, H-6), 10.86 (s, 1H, NH, D_2_O exchangeable); ^13^C NMR (100 MHz, DMSO-d_6_) *δ* 21.4 (CH_3_), 36.4 (CH_2_), 56.0 (2OCH_3_), 60.6 (OCH_3_), 106.8 (trimethoxyphenyl C-2, C-6), 108.3 (pyrimidine C-5), 118.8 (phenyl C-2, C-6), 121.5 (CO*C*H=CH), 127.9 (*p*-tolyl C-2, C-6), 129.3 (trimethoxyphenyl C-1), 129.9 (*p*-chlorophenyl C-2, C-6), 130.4 (*p*-chlorophenyl C-3, C-5), 130.4 (*p*-tolyl C-3, C-5), 130.7 (phenyl C-3, C-5), 132.8 (*p*-tolyl C-4), 133.3 (*p*-tolyl C-1), 135.1 (phenyl C-4), 136.6 (*p*-chlorophenyl C-1), 140.0 (*p*-chlorophenyl C-4), 142.0 (trimethoxyphenyl C-4), 144.0 (phenyl C-1), 144.4 (COCH=*C*H), 153.5 (trimethoxyphenyl C-3, C-5), 163.4 (pyrimidine C-6), 164.9 (pyrimidine C-4), 167.6 (pyrimidine C-2), 171.1 (CONH), 187.8 (CO); Anal. Calcd. for C_37_H_32_ClN_3_O_5_S (665.18): C, 66.71; H, 4.84; N, 6.31. Found: C, 66.57; H, 4.76; N, 6.37.

##### *(*ZE*)-2-[4-(4-Chlorophenyl)-6-(4-nitrophenyl)pyrimidin-2-ylthio]-*N*-{4-[3-(4-methoxyphenyl)acryloyl]phenyl}acetamide (9m)*

Yield 55%; yellow powder; mp 190–192 °C; IR (cm^−1^): 3403 (NH), 3066 (CH aromatic), 2927 (CH aliphatic), 1658 (broad, 2C=O); ^1^H NMR (400 MHz, DMSO-d_6_) *δ* 3.83 (s, 3H, OCH_3_), 4.29 (s, 2H, CH_2_), 7.03 (d, *J*= 8.8 Hz, 2H, *p*-methoxyphenyl H-3, H-5), 7.55 (d, *J*= 8.8 Hz, 2H, *p*-chlorophenyl H-3, H-5), 7.72 (d, *J*= 11.6 Hz, 1H, COC*H*=CH), 7.77–7.93 (m, 5H, COCH=C*H*, *p*-methoxyphenyl H-2, H-6, phenyl H-2, H-6), 8.17 (d, *J*= 8.8 Hz, 2H, phenyl H-3, H-5), 8.28 (d, *J*= 8.8 Hz, 2H, *p*-nitrophenyl H-2, H-6), 8.40 (d, *J*= 8.8 Hz, 2H, *p*-chlorophenyl H-2, H-6), 8.50 (s, 1H, pyrimidine H-5), 8.58 (d, *J*= 8.8 Hz, 2H, *p*-nitrophenyl H-3, H-5), 10.89 (s, 1H, NH, D_2_O exchangeable); ^13^C NMR (100 MHz, DMSO-d_6_) *δ* 38.8 (CH_2_), 55.8 (OCH_3_), 109.7 (pyrimidine C-5), 114.8 (*p*-methoxyphenyl C-3, C-5), 118.8 (phenyl C-2, C-6), 120.1 (CO*C*H=CH), 123.2 (*p*-nitrophenyl C-3, C-5), 124.3 (*p*-nitrophenyl C-2, C-6), 125.8 (*p*-chlorophenyl C-2, C-6), 127.9 (*p*-methoxyphenyl C-1), 129.4 (*p*-chlorophenyl C-3, C-5), 129.4 (*p*-methoxyphenyl C-2, C-6), 133.5 (phenyl C-3, C-5), 133.8 (*p*-chlorophenyl C-1), 134.4 (*p*-chlorophenyl C-4), 134.5 (phenyl C-4), 141.9 (*p*-nitrophenyl C-1), 144.3 (phenyl C-1), 145.4 (COCH=*C*H), 149.2 (*p*-nitrophenyl C-4), 161.8 (*p*-methoxyphenyl C-4), 162.4 (pyrimidine C-6), 164.6 (pyrimidine C-4), 167.8 (pyrimidine C-2), 171.5 (CONH), 187.5 (CO); Anal. Calcd. for C_34_H_25_ClN_4_O_5_S (636.12): C, 64.10; H, 3.96; N, 8.79. Found: C, 64.24; H, 4.06; N, 8.47.

##### *(*ZE*)-2-[4-(4-Chlorophenyl)-6-(4-nitrophenyl)pyrimidin-2-ylthio]-*N*-{4-[3-(3,4-dimethoxyphenyl)acryloyl]phenyl}acetamide (9n)*

Yield 53%; yellow powder; mp 147–149 °C; IR (cm^−1^): 3256 (NH), 3079 (CH aromatic), 2922 (CH aliphatic), 1660 (broad, 2C=O); ^1^H NMR (400 MHz, DMSO-d_6_) *δ* 3.82 (s, 3H, OCH_3_), 3.87 (s, 3H, OCH_3_), 4.29 (s, 2H, CH_2_), 7.03 (d, *J*= 8.4 Hz, 1H, dimethoxyphenyl H-5), 7.39 (d, *J*= 8.4 Hz, 1H, dimethoxyphenyl H-6), 7.53–7.55 (m, 3H, *p*-chlorophenyl H-3, H-5, dimethoxyphenyl H-2), 7.70 (d, *J*= 15.6 Hz, 1H, COC*H*=CH), 7.82–7.87 (m, 3H, *p*-chlorophenyl H-2, H-6, COCH=C*H*), 8.19 (d, *J*= 8.8 Hz, 2H phenyl H-2, H-6), 8.27 (d, *J*= 8.8 Hz, 2H phenyl H-3, H-5), 8.38 (d, *J*= 8.4 Hz, 2H *p*-nitrophenyl H-2, H-6), 8.49 (s, 1H, pyrimidine H-5), 8.57 (d, *J*= 8.4 Hz, 2H, *p*-nitrophenyl H-3, H-5), 10.88 (s, 1H, NH, D_2_O exchangeable); ^13^C NMR (100 MHz, DMSO-d_6_) *δ* 36.5 (CH_2_), 56.0 (OCH_3_), 56.1 (OCH_3_), 109.9 (pyrimidine C-5), 111.0 (dimethoxyphenyl C-2), 111.9 (dimethoxyphenyl C-5), 118.8 (phenyl C-2, C-6), 119.8 (dimethoxyphenyl C-6), 124.2 (*p*-nitrophenyl C-3, C-5), 124.3 (CO*C*H=CH), 128.0 (*p*-nitrophenyl C-2, C-6), 129.2 (dimethoxyphenyl C-1), 129.4 (*p*-chlorophenyl C-2, C-6), 129.8 (*p*-chlorophenyl C-3, C-5), 130.3 (phenyl C-3, C-5), 133.1 (*p*-chlorophenyl C-1), 134.7 (*p*-chlorophenyl C-4), 137.0 (phenyl C-4), 142.1 (*p*-nitrophenyl C-1), 143.8 (phenyl C-1), 144.4 (COCH=*C*H), 149.4 (*p*-nitrophenyl C-4), 151.6 (dimethoxyphenyl C-4), 152.3 (dimethoxyphenyl C-3), 162.7 (pyrimidine C-6), 164.1 (pyrimidine C-4), 167.5 (pyrimidine C-2), 171.6 (CONH), 187.8 (CO); Anal. Calcd. for C_35_H_27_ClN_4_O_6_S (666.13): C, 63.01; H, 4.08; N, 8.40. Found: C, 62.89; H, 4.15; N, 8.37.

##### *(*ZE*)-2-[4-(4-Chlorophenyl)-6-(4-nitrophenyl)pyrimidin-2-ylthio]-*N*-{4-[3-(3,4,5-trimethoxyphenyl)acryloyl]phenyl}acetamide (9o)*

Yield 47%; yellow powder; mp 268–270 °C; IR (cm^−1^): 3371 (NH), 3059 (CH aromatic), 2935 (CH aliphatic), 1656 (broad, 2C=O); ^1^H NMR (400 MHz, DMSO-d_6_) *δ* 3.72 (s, 3H, OCH_3_), 3.87 (s, 6H, 2OCH_3_), 4.29 (s, 2H, CH_2_), 7.24 (s, 2H, trimethoxyphenyl H-2, H-6), 7.55 (d, *J*= 8.8 Hz, 2H, *p*-chlorophenyl H-3, H-5), 7.70 (d, *J*= 15.6 Hz, 1H, COC*H*=CH), 7.87 (d, *J*= 8.8 Hz, 2H, *p*-chlorophenyl H-2, H-6), 7.92 (d, *J*= 15.6 Hz, 1H, COCH=C*H*), 8.21 (d, *J*= 8.8 Hz, 2H, phenyl H-2, H-6), 8.28 (d, *J*= 8.8 Hz, 2H, phenyl H-3, H-5), 8.39 (d, *J*= 8.8 Hz, 2H, *p*-nitrophenyl H-2, H-6), 8.51 (s, 1H, pyrimidine H-5), 8.58 (d, *J*= 8.8 Hz, 2H, *p*-nitrophenyl H-3, H-5), 10.89 (s, 1H, NH, D_2_O exchangeable); ^13^C NMR (100 MHz, DMSO-d_6_) *δ* 36.5 (CH_2_), 56.6 (2OCH_3_), 60.6 (OCH_3_), 106.9 (trimethoxyphenyl C-2, C-6), 110.0 (pyrimidine C-5), 118.8 (phenyl C-2, C-6), 121.5 (CO*C*H=CH), 124.3 (*p*-nitrophenyl C-3, C-5), 129.3 (*p*-nitrophenyl C-2, C-6), 129.4 (*p*-chlorophenyl C-2, C-6), 129.8 (*p*-chlorophenyl C-3, C-5), 130.5 (trimethoxyphenyl C-1), 130.8 (phenyl C-3, C-5), 132.9 (*p*-chlorophenyl C-1), 134.8 (*p*-chlorophenyl C-4), 137.0 (phenyl C-4), 140.0 (trimethoxyphenyl C-4), 142.1 (phenyl C-1), 143.9 (*p*-nitrophenyl C-1), 144.4 (COCH=*C*H), 149.5 (*p*-nitrophenyl C-4), 153.5 (trimethoxyphenyl C-3, C-5), 162.8 (pyrimidine C-6), 164.2 (pyrimidine C-4), 167.5 (pyrimidine C-2), 171.6 (CONH), 187.8 (CO); EIMS (*m/z*): 696.20 (M^+^, 0.11%), 86.15 (100%); Anal. Calcd. for C_36_H_29_ClN_4_O_7_S (696.14): C, 62.02; H, 4.19; N, 8.04. Found: C, 61.98; H, 4.35; N, 7.98.

##### (ZE*)-2-{4-[4-(2-Chloroethoxyphenyl)phenyl]-6-(4-chlorophenyl)pyrimidin-2-ylthio}-*N*-{4-[3-(4-methoxyphenyl)acryloyl]phenyl}acetamide (9p)*

Yield 52%; yellow powder; mp 136–138 °C; IR (cm^−1^): 3181 (NH), 3043 (CH aromatic), 2927 (CH aliphatic), 1663 (broad, 2C=O); ^1^H NMR (400 MHz, DMSO-d_6_) *δ* 3.83 (s, 3H, OCH_3_), 3.97 (t, *J*= 6.9 Hz, 2H, CH*_2_*Cl), 4.25 (s, 2H, CH_2_), 4.32 (t, *J*= 6.9 Hz, 2H, O*C*H*_2_*), 6.90–7.14 (m, 4H, methoxyphenyl H-3, H-5, *p*-chloroethoxyphenyl H-3, H-5), 7.43–7.69 (m, 10H, COC*H*=CH, COCH=C*H*, phenyl H-2, H-6, *p*-methoxyphenyl H-2, H-6, *p*-chloroethoxyphenyl H-2, H-6, *p*-chlorophenyl H-3, H-5), 8.12–8.35 (m, 5H, phenyl H-3, H-5, pyrimidine H-5, *p*-chlorophenyl H-2, H-6), 10.88 (s, 1H, NH, D_2_O exchangeable); ^13^C NMR (100 MHz, DMSO-d_6_) *δ* 36.2 (CH_2_), 43.4 (CH_2_Cl), 54.6 (OCH_3_), 55.8 (OCH_2_), 107.8 (pyrimidine C-5), 114.8 (*p*-methoxyphenyl C-3, C-5), 115.3 (*p*-chloroethoxyphenyl C-3, C-5), 118.8 (phenyl C-2, C-6), 121.8 (CO*C*H=CH), 127.4 (*p*-chloroethoxyphenyl C-1), 128.5 (*p*-methoxyphenyl C-1), 128.6 (*p*-chlorophenyl C-2, C-6), 129.4 (*p*-chlorophenyl C-3, C-5), 129.6 (*p*-methoxyphenyl C-2, C-6), 129.6 (*p*-chloroethoxyphenyl C-2, C-6), 130.7 (*p*-chlorophenyl C-1), 132.7 (phenyl C-3, C-5), 133.9 (*p*-chlorophenyl C-4), 140.1 (phenyl C-4), 143.0 (phenyl C-1), 144.4 (COCH=*C*H), 159.4 (*p*-chloroethoxyphenyl C-4), 159.9 (*p*-methoxyphenyl C-4), 162.8 (pyrimidine C-6), 164.3 (pyrimidine C-4), 167.3 (pyrimidine C-2), 171.4 (CONH), 187.8 (CO); Anal. Calcd. for C_36_H_29_Cl_2_N_3_O_4_S (669.13): C, 64.48; H, 4.36; N, 6.27. Found: C, 64.36; H, 4.19; N, 6.35.

##### *(*ZE*)-2-{4-[4-(2-Chloroethoxyphenyl)phenyl]-6-(4-chlorophenyl)pyrimidin-2-ylthio}-*N*-{4-[3-(3,4-dimethoxyphenyl)acryloyl]phenyl}acetamide (9q)*

Yield 49%; yellow powder; mp 210–212 °C; IR (cm^−1^): 3429 (NH), 3061 (CH aromatic), 2927 (CH aliphatic), 1663 (broad, 2C=O); ^1^H NMR (400 MHz, DMSO-d_6_) *δ* 3.72–3.82 (m, 5H, OCH_3_ and CH_2_Cl), 3.83–3.88 (m, 5H, OCH_3_ and OCH_2_), 4.34 (s, 2H, CH_2_), 6.98–7.15 (m, 4H, dimethoxyphenyl H-5, H-6, *p*-chloroethoxyphenyl H-3, H-5), 7.31–7.40 (m, 2H, dimethoxy H-2 and COC*H*=CH), 7.52–7.70 (m, 4H, *p*-chlorophenyl H-3, H-5 and *p*-chloroethoxyphenyl H-2, H-6), 7.75–7.88 (m, 4H, phenyl H-2, H-6 and *p*-chlorophenyl H-2, H-6), 7.95 (d, *J*= 8.8 Hz, 2H, phenyl H-3, H-5), 8.28 (d, *J*= 12.0 Hz, 1H, COCH=C*H*), 8.46 (s, 1H, pyrimidine H-5), 10.76 (s, 1H, NH, D_2_O exchangeable); ^13^C NMR (100 MHz, DMSO-d_6_) *δ* 38.4 (CH_2_), 42.9 (CH_2_Cl), 56.1 (OCH_3_), 56.6 (OCH_3_), 68.6 (OCH_2_), 107.3 (pyrimidine C-5), 111.5 (dimethoxyphenyl C-2), 112.7 (dimethoxyphenyl C-5), 114.9 (*p*-chloroethoxyphenyl C-3, C-5), 121.1 (phenyl C-2, C-6), 121.3 (CO*C*H=CH), 122.5 (dimethoxyphenyl C-6), 127.3 (dimethoxyphenyl C-1), 127.4 (*p*-chloroethoxyphenyl C-1), 128.1 (*p*-chloroethoxyphenyl C-2, C-6), 128.9 (*p*-chlorophenyl C-2, C-6), 129.3 (*p*-chlorophenyl C-3, C-5), 131.4 (phenyl C-3, C-5), 133.9 (*p*-chlorophenyl C-1), 134.3 (*p*-chlorophenyl C-4), 135.5 (phenyl C-4), 144.3 (phenyl C-1), 145.1 (COCH=*C*H), 149.3 (dimethoxyphenyl C-4), 149.7 (dimethoxyphenyl C-3), 151.1 (*p*-chloroethoxyphenyl C-4), 162.5 (pyrimidine C-6), 164.4 (pyrimidine C-4), 168.7 (pyrimidine C-2), 172.7 (CONH), 189.7 (CO); Anal. Calcd. for C_37_H_31_Cl_2_N_3_O_5_S (699.14): C, 63.43; H, 4.46; N, 6.00. Found: C, 63.33; H, 4.27; N, 6.11.

##### *(*ZE*)-2-{4-[4-(2-Chloroethoxyphenyl)phenyl]-6-(4-chlorophenyl)pyrimidin-2-ylthio}-*N*-{4-[3-(3,4,5-trimethoxyphenyl)acryloyl]phenyl}acetamide (9r)*

Yield 53%; yellow powder; mp 221–223 °C; IR (cm^−1^): 3421 (NH), 3061 (CH aromatic), 2931 (CH aliphatic), 1665 (broad, 2C=O); ^1^H NMR (400 MHz, DMSO-d_6_) *δ* 3.70 (s, 3H, OCH_3_), 3.85 (s, 6H, 2OCH_3_), 3.96 (t, *J*= 7.2 Hz, 2H, CH_2_Cl), 4.24 (s, 2H, CH_2_), 4.45 (t, *J*= 7.2 Hz, 2H, OCH_2_), 7.03–7.20 (m, 4H, trimethoxyphenyl H-2, H-6, chloroethoxyphenyl H-3, H-5), 7.29 (d, *J*= 12.0 Hz, 1H, COC*H*=CH), 7.51–7.60 (m, 4H, *p*-chlorophenyl H-3, H-5, chloroethoxyphenyl H-2, H-6), 7.87–7.96 (m, 3H, phenyl H-2, H-6, COCH=C*H*), 8.18–8.32 (m, 5H, phenyl H-3, H-5, *p*-chlorophenyl H-2, H-6, pyrimidine H-5), 10.91 (s, 1H, NH, D_2_O exchangeable); ^13^C NMR (100 MHz, DMSO-d_6_) *δ* 36.8 (CH_2_), 43.9 (CH_2_Cl), 55.5 (2OCH_3_), 57.8 (OCH_2_), 60.8 (OCH_3_), 106.7 (trimethoxyphenyl C-2, C-6), 109.4 (pyrimidine C-5), 114.9 (*p*-chloroethoxyphenyl C-3, C-5), 118.8 (phenyl C-2, C-6), 121.5 (CO*C*H=CH), 126.4 (trimethoxyphenyl C-1), 127.4 (*p*-chloroethoxyphenyl C-1), 128.1 (*p*-chloroethoxyphenyl C-2, C-6), 129.5 (*p*-chlorophenyl C-2, C-6), 129.8 (*p*-chlorophenyl C-3, C-5), 132.7 (phenyl C-3, C-5), 135.4 (*p*-chlorophenyl C-1), 136.8 (*p*-chlorophenyl C-4), 140.3 (phenyl C-4), 141.7 (trimethoxyphenyl C-4), 143.0 (phenyl C-1), 144.4 (COCH=*C*H), 153.5 (trimethoxyphenyl C-3, C-5), 159.4 (*p*-chloroethoxyphenyl C-4), 162.2 (pyrimidine C-6), 164.1 (pyrimidine C-4), 167.7 (pyrimidine C-2), 171.7 (CONH), 187.7 (CO); EIMS (*m/z*): 728.10 (M-1, 0.28%), 58.10 (100%); Anal. Calcd. for C_38_H_33_Cl_2_N_3_O_6_S (729.15): C, 62.47; H, 4.55; N, 5.75. Found: C, 62.53; H, 4.76; N, 5.58.

### Biological evaluations

#### Cytotoxic assay

To investigate cytotoxic activity of the final target compounds **9a–r**, MTT assay was performed. Three different cell lines were used, leukaemia (K-562), breast (MCF-7) and colon (HT-29) cell lines. Cisplatin and erlotinib were the reference drugs used in this study. Half maximal concentration at which 50% of cells were viable was calculated as IC_50_ in μM, according to cytotoxic assay reported protocol^47^.

#### STAT3/STAT5a assays

Both K-562 and MCF-7 cell lines were seeded overnight in plates, then 10 µM of test compounds (**9a** and **9r** for MCF-7 cells; **9d**, **9f**, and **9n** for K-562 cells) or reference drug pacritinib was added for 24 h. A nuclear extract kit was used to extract nuclear fractions from treated cells using the manufacture’s procedure. STAT3 and STAT5a activations were analysed using the collected nuclear extracts (20 µg) through TransAM STAT3 and STAT5a activation assay guided by the manufacture’s protocol. The obtained results were expressed in the form of mean ± SD. Each experiment was done in triplicate.

### Biological properties

The target compounds **9a–r** were drawn using ChemDraw Ultra 10.0. Biological properties and drug likeness were predicted using online computational tool Molinspiration^48^.

#### Predicted pharmacokinetic and toxicity properties

Pharmacokinetic properties (absorption, distribution, metabolism, and excretion) through determination of human intestinal absorption (HIA), *in vitro* caco-2 cell permeability, *in vitro* Madin-Darby Canine Kidney (MDCK) cell permeability, plasma protein binding (PPB), blood–brain binding (BBB), skin permeability, p-glycoprotein (Pgp), and cytochrome p450 isoforms inhibition data, in addition to toxicity (Ames test, rodent carcinogenicity assay and hERG-inhibition) were evaluated through preADMET online server^49^.

### Statistical analysis

Data obtained were expressed as means ± standard deviations (SDs). The results were considered significant when *p ˂ 0.05 or **p ˂ 0.005 using Student’s *t*-test was compared to reference drugs. The obtained values were representative of triplicate independent experiments.

## Results and discussion

### Chemistry

**Scheme 1.**
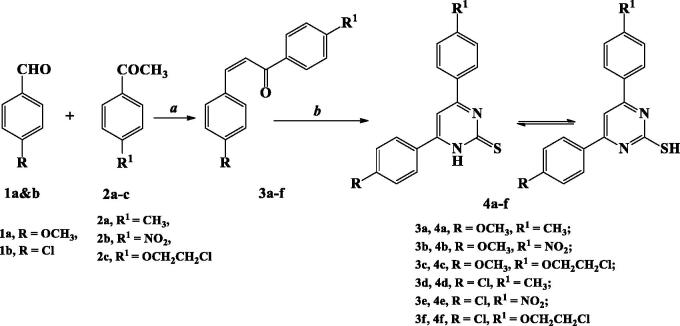
Synthesis of 2-thiopyrimidine derivatives **4a–f**.

**Scheme 2.**
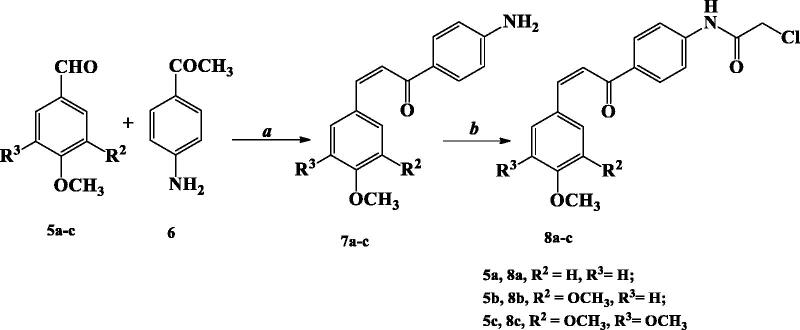
Synthesis of chloroacetyl aminochalcone derivatives **8a–c**.

**Scheme 3.**
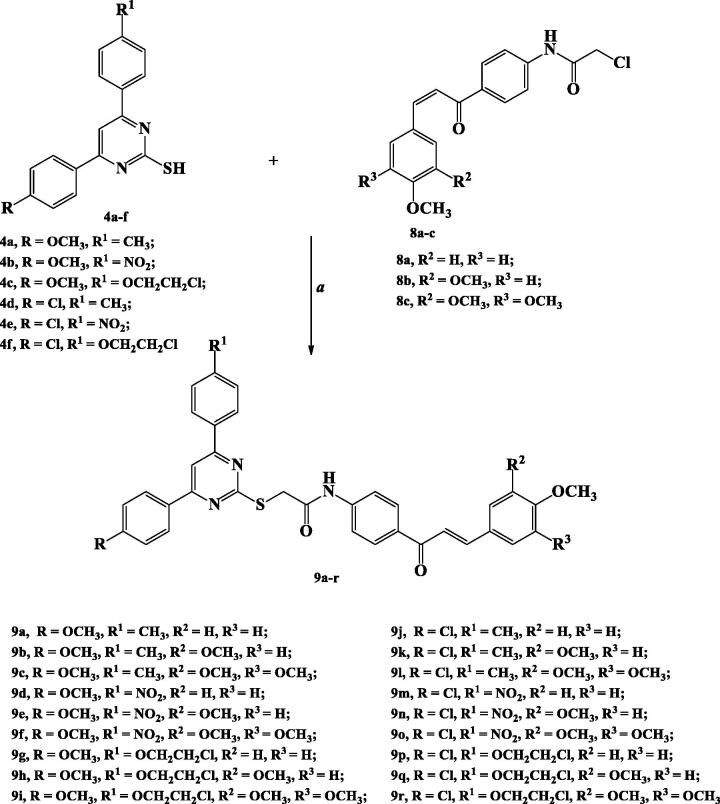
Synthesis of the target compounds **9a–r**.

The target 2-TP/chalcone hybrids **9a–r** were prepared from two synthesised starting materials **4a–f** and **8a–c**, as depicted in Schemes 1–3.

Heating under reflux condition chalcone derivatives **3a–f** (synthesised from condensation of *p*-methoxy/chlorobenzaldehyde **1a&b** with *p*-methyl/nitro/or ethoxychloroacetophenone **2a–c**) and thiourea in presence of KOH afforded 2-TP derivatives **4a–f**. The method was reported for compounds **4b** and **4d**^39^^,^^50^ (Scheme 1).

The other starting materials, chloroacetyl chalcone derivatives **8a–c** were obtained by stirring *p*-aminochalcone derivatives **7a–c** with chloroacetyl chloride, K_2_CO_3_ in chloroform at room temperature^24^ (Scheme 2).

*S*-Alkylation of 2-TPs **4a–f** with acetylated chalcones **8a–c** was achieved in acetonitrile using TEA as a base catalysis to obtain the target compounds **9a–r** in 47–82% yield.

^1^H NMR and ^13^C NMR spectroscopic tools were used to confirm formation of the target derivatives **9a–r**. Thus, ^1^H NMR spectra of compounds **9a–r** displayed a singlet signal at *δ* 3.82–4.84 ppm attributed to (SCH_2_CO) protons. Additionally, protons of chalcone fragment appeared as two doublet signals at *δ* 7.29–7.72 ppm and 7.69–8.31 ppm with coupling constant *J*= 11.6–15.6 Hz. Furthermore, amide NH proton appeared as a singlet signal at *δ* 10.71–10.91 ppm.

^13^C NMR spectra of compounds **9a–r** showed appearance of a peak at *δ* 36.21–43.44 ppm characterised to SCH_2_ carbon. Moreover, two carbonyl carbons at *δ* 170.71–173.07 ppm and 187.58–190.01 ppm related to (NHCO) and (C=O), respectively, were also appeared (Scheme 3).

### Biological activity

#### Cytotoxic activity

All target compounds **9a–r** were screened against three different cancer cell lines, leukaemia (K-562), breast (MCF-7), and colon (HT-29). MTT assay was used. Both cisplatin and erlotinib were used as the reference drugs. Cytotoxicity results are recorded in Table 1.

**Table 1.** Cytotoxicity results of pyrimidine/chalcone hybrids **9a–r** against three different cancer cell lines.


Compound	R	R^1^	R^2^	R^3^	(IC_50_ μM)±SD		
					K-562	MCF-7	HT-29
**9a**	OCH_3_	CH_3_	H	H	18.40 ± 0.76	3.56 ± 0.14	2.20 ± 0.04
**9b**	OCH_3_	CH_3_	CH_3_	H	3.62 ± 0.08	22.45 ± 1.57	16.36 ± 0.76
**9c**	OCH_3_	CH_3_	OCH_3_	OCH_3_	11.42 ± 0.04	4.25 ± 0.13	8.70 ± 0.28
**9d**	OCH_3_	NO_2_	H	H	0.77 ± 0.03	14.16 ± 0.74	25.92 ± 1.67
**9e**	OCH_3_	NO_2_	OCH_3_	H	7.05 ± 0.28	18.74 ± 0.92	7.31 ± 0.36
**9f**	OCH_3_	NO_2_	OCH_3_	OCH_3_	1.37 ± 0.03	5.77 ± 0.22	9.41 ± 0.43
**9g**	OCH_3_	OCH_2_CH_2_Cl	H	H	3.17 ± 0.11	10.40 ± 0.64	10.55 ± 0.47
**9h**	OCH_3_	OCH_2_CH_2_Cl	OCH_3_	H	7.07 ± 0.31	7.70 ± 0.32	18.77 ± 0.88
**9i**	OCH_3_	OCH_2_CH_2_Cl	OCH_3_	OCH_3_	9.71 ± 0.41	11.47 ± 0.81	11.47 ± 0.23
**9j**	Cl	CH_3_	H	H	4.26 ± 0.06	6.26 ± 0.24	6.26 ± 0.34
**9k**	Cl	CH_3_	OCH_3_	H	9.95 ± 0.29	28.65 ± 1.39	5.61 ± 0.18
**9l**	Cl	CH_3_	OCH_3_	OCH_3_	42.60 ± 1.99	13.46 ± 0.62	2.37 ± 0.07
**9m**	Cl	NO_2_	H	H	3.86 ± 0.14	3.90 ± 0.09	7.90 ± 0.22
**9n**	Cl	NO_2_	OCH_3_	H	1.05 ± 0.02	17.36 ± 0.75	2.10 ± 0.06
**9o**	Cl	NO_2_	OCH_3_	OCH_3_	12.35 ± 0.72	3.62 ± 0.084	3.62 ± 0.08
**9p**	Cl	OCH_2_CH_2_Cl	H	H	1.74 ± 0.04	11.64 ± 0.49	11.64 ± 0.63
**9q**	Cl	OCH_2_CH_2_Cl	OCH_3_	H	5.77 ± 0.16	6.81 ± 0.25	6.06 ± 0.29
**9r**	Cl	OCH_2_CH_2_Cl	OCH_3_	OCH_3_	10.67 ± 0.77	1.37 ± 0.07	4.74 ± 0.27
**Cisplatin**					2.31 ± 0.09	6.62 ± 0.29	1.12 ± 0.06
**Erlotinib**					9.85 ± 0.51	10.64 ± 0.58	9.20 ± 0.41

Regarding cytotoxic activity of the test compounds against leukaemia (K-562) cell line, compounds **9d**, **9f**, **9n**, and **9p** were the most potent compounds with IC_50_ ranged from 0.77 to 1.74 μM if compared to cisplatin, the reference drug (IC_50_=2.31 μM). Their common feature was presence of one or more *para* substituted phenyl ring(s) with electron withdrawing group (NO_2_, Cl) at pyrimidine core.

Compounds **9b**, **9e**, **9g–j**, **9m**, and **9q** exhibited potent inhibitory activity with IC_50_ values ranged from 3.17 to 9.71 μM, if compared to the second reference erlotinib (IC_50_: 9.85 μM). *P*-Methoxyacyloyl derivative **9k**, with IC_50_ value = 9.95 μM, was nearly equal in potency to erlotinib. Compounds **9a**, **9c**, **9o**, and **9r** showed moderate inhibitory activity (IC_50_=10.67–18.40 μM). The lowest inhibitory activity was observed in compound **9l** (IC_50_=42.60 μM), bearing *p*-chlorophenyl and *p*-tolyl rings at pyrimidine scaffold, beside, trimethoxyphenyl chalcone hybrid.

Concerning MCF-7 cell line, the most active derivative was **9r** (IC_50_=1.37 μM) compared to the reference drug cisplatin (IC_50_=6.62 μM). It is characterised by presence of *p*-chlorophenyl ring and *p*-chloroethoxyphenyl ring at pyrimidine core together with trimethoxy chalcone part.

Other compounds exerted excellent activity were **9a**, **9c**, **9f**, **9j**, **9m**, and **9o** (IC_50_=3.56–6.26 μM). Additionally, compound **9q** was nearly equipotent to the reference drug cisplatin (IC_50_=6.81 μM). Derivatives **9g** and **9h** with IC_50_=10.40 and 7.70 μM, respectively, were more potent than erlotinib (IC_50_=10.64 μM).

Moderate activity was observed in compounds **9d**, **9e**, **9i**, **9l**, **9n**, and **9p** (IC_50_=11.47–18.74 μM).

Compounds **9b** and **9k** showed weak inhibitory activity with IC_50_ values equal to 22.45 and 28.65 μM, respectively. Both of them have dimethoxyphenyl ring on chalcone part, and *p*-tolyl ring at pyrimidine core.

By inspecting cytotoxicity results of HT-29 cell line, compounds **9a**, **9l**, and **9n** showed IC_50_ (2.10–2.37 μM) near in potency to the reference drug cisplatin (IC_50_=1.12 μM). Compounds **9c**, **9e**, **9j**, **9k**, **9m**, **9o**, **9q**, and **9r** showed significant activity with IC_50_ values between 3.62 and 8.70 μM, compared to erlotinib (IC_50_=9.20 μM). While, rest of the compounds exhibited weak inhibitory activity (IC_50_=9.41–25.92 μM). Results showed that no effect was observed regarding substituents on the two hybrid structures pyrimidine and chalcone.

Finally, dual cytotoxic activity was observed for compound **9f** (against K-562 and MCF-7 cell lines), and for compound **9a** (on MCF-7 and HT-29 cell lines) and **9n** (against K-562 and HT-29 cell lines).

#### Cytotoxicity against normal cell line (WI38)

**Figure 2.**
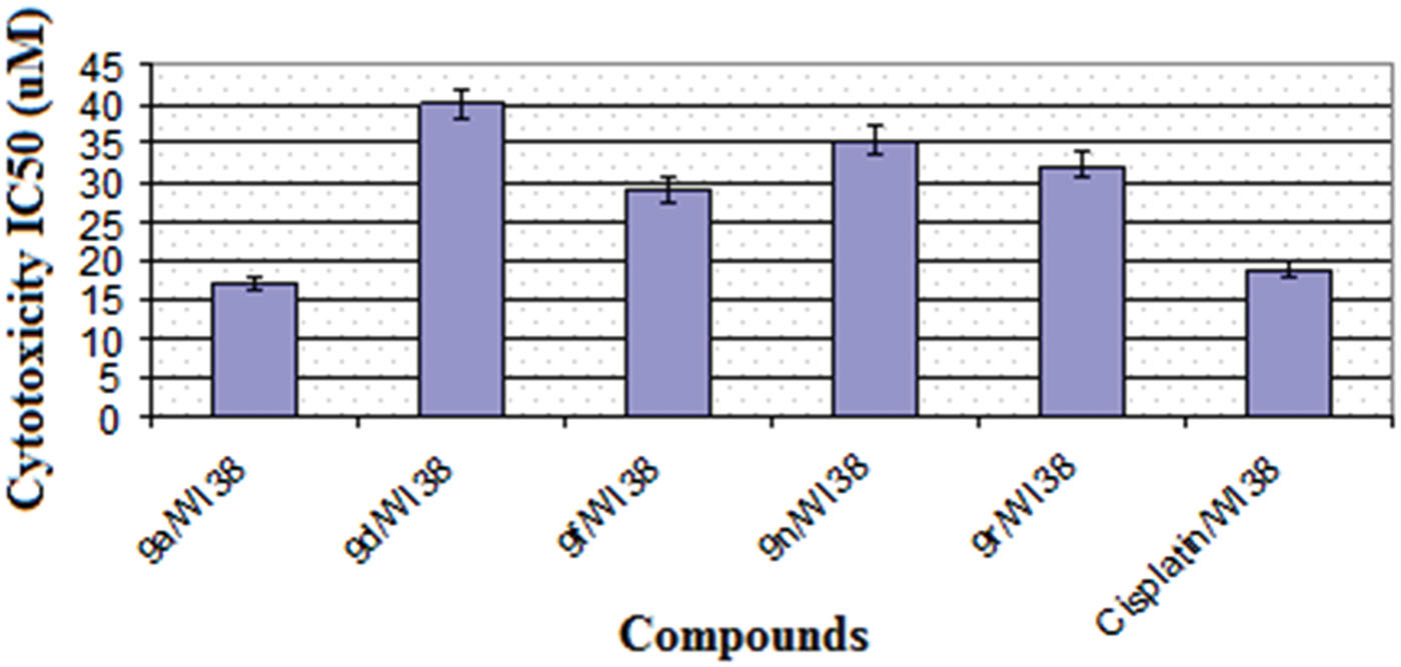
Cytotoxicity (IC_50_) of the most active derivatives and cisplatin against WI38 cell line.

To know cytotoxicity of the most active compounds, they were tested against normal human fibroblast cell line (WI38) and IC_50_ values are represented as in Figure 2. Cisplatin was used as a reference drug. All the test compounds showed higher IC_50_ values (29.19–40.13 µM) than the reference drug (18.86 µM) except compound **9a** which exerted cytotoxic activity (IC_50_=17.09 µM) slightly less than cisplatin.

#### STAT3 and STAT5a inhibitory activity determination

The most active compounds in cytotoxic assay against leukaemia cell line K-562 and human breast adenocarcinoma cells MCF-7 were further tested as inhibitors for STAT3 and STAT5a enzymes. Pacritinib, an inhibitor for both STAT3 and STAT5a^49^ was used in this study as a reference drug.

The results are listed in Table 2. They indicated that the test compounds showed inhibitory activity against both STAT3 and STAT5a. Compounds **9d**, **9n**, and **9r** were the most active against STAT3. Additionally, compound **9n** was the most effective as STAT5a inhibitor. Compounds **9a**, **9f**, and **9r** had also strong inhibitory activity against STAT5a. Dual inhibitory activity against STAT3 and STAT5a was observed mainly in compound **9n**. For this compound, both phenyl rings on pyrimidine core were *para*-substituted with electron withdrawing groups (NO_2_ and Cl), beside presence of disubstituted methoxyphenyl ring at chalcone hybrid.

**Table 2.** STAT3 and STAT5a inhibitory activity of compounds **9a**, **9d**, **9f**, **9n**, **9r** and reference drug pacritinib.

Compound/no.	Inhibition IC_50_ (µM)	
	STAT3	STAT5a
**9a**/MCF7	242.53 ± 9.24	83.78 ± 3.28
**9d**/K562	160.01 ± 4.59	116.31 ± 4.13
**9f**/K562	244.74 ± 11.07	77.65 ± 2.91
**9n**/K562	113.31 ± 3.22	50.75 ± 1.26
**9r**/MCF7	148.69 ± 3.81	63.24 ± 1.57
**Pacritinib**/MCF7	79.47 ± 2.17	54.35 ± 1.09
**Pacritinib**/K562	65.49 ± 2.55	69.81 ± 1.82

### Biological properties

Molinspiration was used to predict bioactivity scores for all the target compounds **9a–r**. The obtained results are recorded in Table 3. It was found that most of the prepared compounds had bioactivity values in the range −0.5 to 0.00. This revealed that the designed pyrimidine/chalcone derivatives might be involved in moderate interactions with G-protein-coupled receptors (GPCRs) and protease inhibitors. However, the bioactivity prediction was not in the standard range against other receptors such as ion channel modulator, kinases and nuclear receptor ligand.

**Table 3.** Biological properties, prediction, and drug likeness of the target compounds.

Compound	GPCR ligand	Ion channel modulator	Kinase inhibitor	Nuclear receptor ligand	Protease inhibitor	Drug likeness score
**9a**	−0.43	−1.03	−0.58	−0.69	−0.43	0.49
**9b**	−0.56	−1.27	−0.78	−0.90	−0.51	0.54
**9c**	−0.73	−1.55	−1.01	−1.17	−0.62	0.96
**9d**	−0.61	−1.25	−0.83	−0.92	−0.53	0.07
**9e**	−0.78	−1.53	−1.07	−1.17	−0.65	0.11
**9f**	−0.99	−1.86	−1.35	−1.50	−0.80	0.49
**9g**	−0.69	−1.44	−0.81	−1.01	−0.56	0.83
**9h**	−0.88	−1.75	−1.08	−1.29	−0.70	0.79
**9i**	−1.11	−2.09	−1.39	−1.64	−0.87	1.18
**9j**	−0.39	−0.94	−0.52	−0.63	−0.44	0.82
**9k**	−0.50	−1.16	−0.68	−0.81	−0.50	0.80
**9l**	−0.64	−1.41	−0.89	−1.06	−0.58	1.07
**9m**	−0.55	−1.13	−0.75	−0.83	−0.52	0.43
**9n**	−0.69	−1.39	−0.95	−1.06	−0.62	0.40
**9o**	−0.88	−1.69	−1.21	−1.36	−0.74	0.63
**9p**	−0.61	−1.31	−0.71	−0.91	−0.54	1.14
**9q**	−0.79	−1.60	−0.96	−1.17	−0.66	1.05
**9r**	−1.00	−1.92	−1.24	−1.49	−0.81	1.40

**Figure 3.**
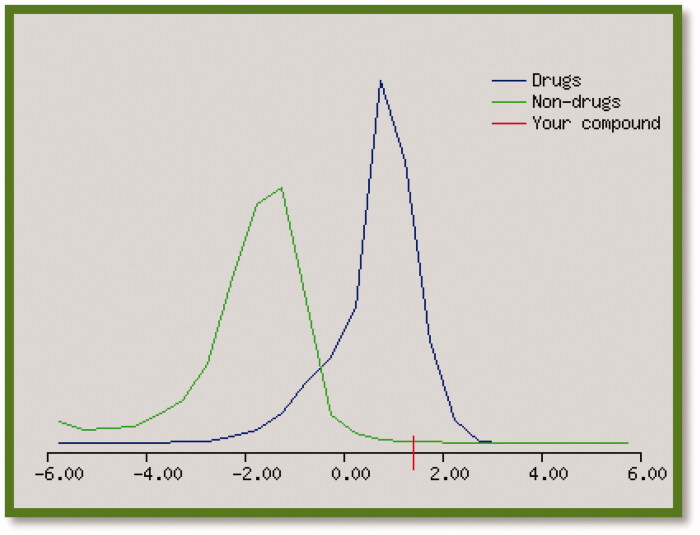
Drug likeness score value (1.40) for compound **9r**.

Drug likeness is a complex balance between various molecular properties like, molecule size, hydrogen bonding characters, electronic distribution and hydrophobicity^51^. The results in Table 3 showed that all the final target compounds had positive predictable score values which stranded for good drug likeness behaviour, especially compound **9r** as represented in Figure 3.

#### Predicted pharmacokinetic and toxicity properties

Prediction of the major pharmacokinetic parameters such as absorption, distribution, metabolism, and excretion, in addition to toxicological properties, such as mutagenicity, carcinogenicity, and cardiac toxicity was estimated using Pharmacokinetics/PreADMET Toxicity Predictor^52^ (Table 4).

**Table 4.** Pharmacokinetic properties assessment of the target synthesised compounds **9a–r**.

Compound	Absorption				Distribution		Metabolism (CYP) and excretion				
	HIA (%)	SP LogP (cm/h)	Caco2 (nm/sc)	MDCK (nm/sc)	BBB (c.brain/c.blood)	PPB %	2C19	2C9	2D6	3A4	Pgp Inh.
**9a**	97.50	−1.72	54.34	0.05	0.03	98.31	No	Yes	No	Yes	Inh.
**9b**	97.36	−1.73	54.31	0.06	0.03	99.63	No	Yes	No	Yes	Inh.
**9c**	97.25	−1.74	54.26	0.06	0.03	97.83	No	Yes	No	Yes	Inh.
**9d**	98.78	−2.26	28.65	0.04	0.49	92.53	No	Yes	No	Yes	Inh.
**9e**	99.22	−2.25	29.60	0.04	0.45	96.23	No	Yes	No	Yes	Inh.
**9f**	99.39	−2.22	30.34	0.04	0.38	96.95	No	Yes	No	Yes	Inh.
**9g**	97.69	−2.09	37.98	0.05	0.02	92.10	No	Yes	No	Yes	Inh.
**9h**	97.54	−2.03	38.68	0.05	0.02	95.16	No	Yes	No	Yes	Inh.
**9i**	97.42	−1.96	39.36	0.05	0.02	95.17	No	Yes	No	Yes	Inh.
**9j**	97.95	−1.74	51.82	0.06	0.08	90.94	No	Yes	No	Yes	Inh.
**9k**	97.83	−1.74	52.19	0.07	0.05	94.17	No	Yes	No	Yes	Inh.
**9l**	97.69	−1.73	52.52	0.07	0.04	94.05	No	Yes	No	Yes	Inh.
**9m**	97.49	−2.28	33.88	0.04	0.37	93.03	No	Yes	No	Yes	Inh.
**9n**	97.96	−2.28	35.90	0.04	0.50	91.43	No	Yes	No	Yes	Inh.
**9o**	98.56	−2.27	37.81	0.04	0.57	92.17	No	Yes	No	Yes	Inh.
**9p**	98.11	−2.14	42.99	0.05	0.05	92.90	No	Yes	No	Yes	Inh.
**9q**	97.99	−2.10	43.47	0.05	0.03	91.65	No	Yes	No	Yes	Inh.
**9r**	97.86	−2.05	43.93	0.05	0.03	91.71	No	Yes	No	Yes	Inh.

Absorption refers to the process by which the drug can go to the systemic circulation through the organs of the body. Several routes for absorption such as oral absorption (human intestinal absorption, HIA), skin permeability (SP, logKp), and permeability through certain cells such as Caco2 (derived from human colon adenocarcinoma cells) and MDCK cells were measured.

By inspecting results recorded in Table 4, it was found that compounds **9a–r** showed good intestinal absorption all above 97.25% (permissible limit: 70–100%abs). Skin permeability was found to be slightly less than acceptable range (−2.5 logKp). Moreover, moderate permeability through *in vitro* Caco2 cells ranged from 54.34 to 28.65 nm/sc were observed. While, low values were detected for *in vitro* MDCK cells.

The second property is the distribution, through which the transformation of the molecules from one tissue or organ to another can be predicted. Blood–brain barrier (BBB) and PPB were two distribution parameters used in this study. BBB permits the diffusion of hydrophobic and small molecules to the brain. It is an important predictor for central nervous system (CNS) drug discovery. Moreover, the measured of percentage of a molecule bound to plasma protein (%PPB) was also helpful in prediction of distribution for the novel target compounds.

Results showed that all the test compounds displayed strong PPB value (90.94–99.63%) indicating prolonged half-lives and limited brain penetration. Consequently, BBB (unbound brain-to-plasma ratio) was low in most compounds except in nitrophenyl containing derivatives **9d–9f** and **9m–9o** (0.37–0.57) which was medium and around the acceptable range to be CNS active compounds (>0.4).

Metabolism, the biotransformation or chemical modification of exogenous compounds to increase their water solubility by increasing their hydrophobicity facilitating their excretion can be predicted either in phase I or phase II. Cytochrome P450 isoforms, calculate the ability of the test compounds to be inhibitor to drug metabolising enzymes such as CYP2C19, CYP2C9, CYP2D6, CYP3A4, and CYP1A2. Moreover, glycoprotein (P-gp) inhibition measured to predict excretion property of the target compounds.

The test compounds showed good inhibitory behaviour for CYP2C9 and CYP3A4 and did not show inhibition behaviour for CYP2C19 and CYP3A4. All compounds had inhibitory effect on P-gp.

Prediction of toxicological behaviour of the test compounds was obtained by measuring AMES test (to predict mutagenicity of the compounds), carcino-Mouse/Rate (to test carcinogenicity of the compounds), and hERG-inhibition (to check cardiac toxicity of the target synthesised molecules) (Table 5). Half of test compounds showed non-mutagenic behaviour in AMES test. All compounds had negative carcinogenic effect in mouse and rats, in addition to medium risk as cardiotoxic agents. From the predicted ADMET properties of the novel synthesised compounds, it was justified that they may have good characters as lead compounds.

**Table 5.** Toxicity assessment of the target synthesised compounds **9a–r**.

Compound	AMES	Carcino-Mouse	Carcino-Rat	hERG-inhibition
**9a**	Mutagen	Negative	Negative	Medium
**9b**	Non-mutagen	Negative	Negative	Medium
**9c**	Non-mutagen	Negative	Negative	Medium
**9d**	Mutagen	Negative	Negative	Medium
**9e**	Mutagen	Negative	Negative	Medium
**9f**	Mutagen	Negative	Negative	Medium
**9g**	Non-mutagen	Negative	Negative	Medium
**9h**	Non-mutagen	Negative	Negative	Medium
**9i**	Non-mutagen	Negative	Negative	Medium
**9j**	Mutagen	Negative	Negative	Medium
**9k**	Mutagen	Negative	Negative	Medium
**9l**	Non-mutagen	Negative	Negative	Medium
**9m**	Mutagen	Negative	Negative	Medium
**9n**	Mutagen	Negative	Negative	Medium
**9o**	Mutagen	Negative	Negative	Medium
**9p**	Non-mutagen	Negative	Negative	Medium
**9q**	Non-mutagen	Negative	Negative	Medium
**9r**	Non-mutagen	Negative	Negative	Medium

### Structure–activity relationship of target compounds

**Figure 4.**
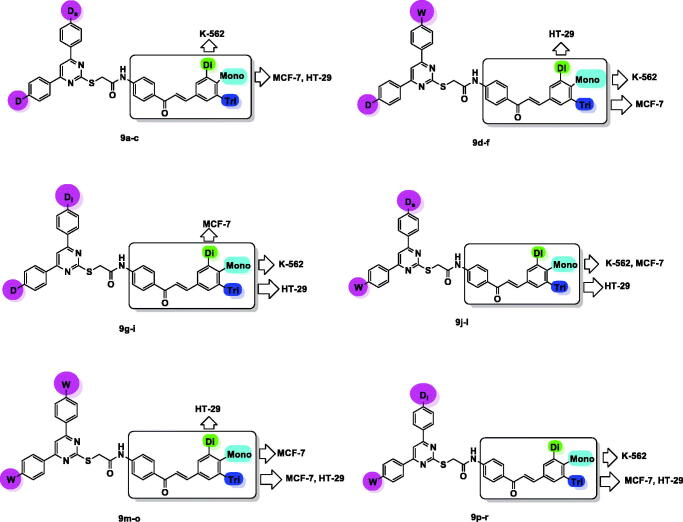
SAR study of the target compounds **9a–r**.

Structure–activity relationship (SAR) study for the target compounds **9a–r** focussed on two important scaffolds, pyrimidine and chalcone. There was a relationship between presence of small electron donating group such as –CH_3_, (D_s_), large electron donating group such as –OCH_2_CH_2_Cl, (D_l_) or electron withdrawing group, –NO_2_, (W) in *para* position of phenyl ring at pyrimidine C-4 and electron donating group, –OCH_3_, (D) or electron withdrawing group, –Cl, (W) in *para* position of phenyl ring at pyrimidine C-6 with that of (mono-, di-, or tri-)methoxyphenyl ring of chalcone part, and between cytotoxic activities on the three different tested cell lines K-562, MCF-7, and HT-29, as represented in Figure 4.

In compounds **9a–c**, pyrimidine core carried *p*-tolyl group and *p*-methoxyphenyl group at C-4 and C-6, respectively. Cytotoxic activity against K-562 cell line was maximised in compound **9b** bearing dimethoxyphenyl chalcone moiety then decreased in **9c** and **9a** (trimethoxyphenyl and methoxyphenyl chalcones, respectively).

The order of reactivity was altered when evaluated against MCF-7 or HT-29 cell lines, where **9a**>**9c**>**9b**.

For compounds **9d–f**, replacement of *p*-tolyl group at pyrimidine C-4 with *p*-nitrophenyl group and keeping *p*-methoxyphenyl group at pyrimidine C-6 constant, led to variation in cytotoxic activity. Thus, compound **9d** (with *p*-methoxyphenyl chalcone part) was the most potent against K-562 cell line, than **9f** (trimethoxyphenyl chalcone analogue) and finally **9e** (dimethoxyphenyl chalcone analogue). While against MCF-7, the order was **9f**>**9d**>**9e**. For HT-29, **9e** was the most potent than **9f** and at last **9d**.

Regarding compounds **9g–i**, they characterised by bearing electron donating groups at *para* position of two phenyl rings at pyrimidine C-4 and C-6; however, presence of large sized electron donating group such as –OCH_2_CH_2_Cl at phenyl ring of pyrimidine C-4 led to increase its lipophilic character.

The order of cytotoxic activity against K-562 was found to be **9g** (methoxyphenyl chalcone)>**9h** (dimethoxyphenyl chalcone)>**9i** (trimethoxyphenyl chalcone), and for MCF-7 cell line was, **9h**>**9g**>**9i**. While converted to be **9i**>**9g**>**9h** in case of HT-29 cell line.

In **9j–l** derivatives, electron withdrawing group (–Cl) was introduced to *para* position of phenyl ring at pyrimidine C-6, while pyrimidine C-4 carried small sized electron donating group (–CH_3_) at *para* position of its phenyl ring.

The most active compound was **9j** (methoxyphenyl chalcone) in both K-562 and MCF-7 cell lines, while **9l** (trimethoxyphenyl chalcone) was the most potent against HT-29 cell line.

Compounds **9m–o**, *p*-tolyl ring were replaced with *p*-nitrophenyl ring at pyrimidine C-4, while *p*-chlorophenyl ring at pyrimidine C-6 was kept constant.

The most potent derivatives against K-562 were dimethoxyphenyl chalcone **9n**, than methoxyphenyl chalcone derivative **9m**. While, trimethoxyphenyl chalcone derivative **9o** and methoxyphenyl chalcone analogue **9m** showed nearly equal potency against MCF-7. For HT-29, **9n** was the most potent than **9o** and finally **9m**.

In compounds **9p–r**, incorporation of *p*-chloroethoxyphenyl group at pyrimidine C-4, while keeping *p*-chlorophenyl group at pyrimidine C-6 constant, resulted in changing order of cytotoxic activity against K-562 to be **9p** (methoxyphenyl chalcone)>**9q** (dimethoxyphenyl chalcone), and still the least potent compound was **9r** (trimethoxyphenyl chalcone). However, for MCF-7 and HT-29, it was observed that **9r** was the most potent than **9q** and finally **9p**.

## Conclusions

A novel series of 2-TP/chalcone hybrids **9a–r** was designed to be as anticancer agents. They were synthesised and identified using different spectroscopic techniques. Their cytotoxic activities against three different cancerous cell lines, K-562, MCF-7, and HT-29 were evaluated. The synthesised compounds showed strong to moderate cytotoxic activities especially against K-562 and MCF-7 cell lines. The highest cytotoxic activity against K-562 cell line was observed in compounds **9d**, **9f**, **9n**, and **9p** with IC_50_ values in the range of 0.77–1.74 µM, compared to the reference drug, cisplatin (IC_50_=2.31 µM). For cytotoxic activity against MCF-7 cell line, compounds **9a**, **9c**, **9f**, **9j**, **9m**, **9o**, and **9r** exhibited the highest activities with IC_50_ values of 1.37–6.26 µM (cisplatin IC_50_=6.62 µM). While, moderate cytotoxic activity was noticed for test compounds against colon HT-29 cell line. The most potent derivatives between them were **9a**, **9l**, and **9n** (IC_50_=2.10–2.37 µM), if compared with cisplatin (IC_50_=1.12 µM).

The most active derivatives **9a**, **9d**, **9f**, **9n**, and **9r** (either against K-562 and/or MCF-7 cell lines) were selected for further evaluation against human normal fibroblast cells (WI38). All of them had IC_50_ values (29.19–40.13 µM) higher than that of the reference cisplatin (IC_50_=18.86 µM), except **9a** analogue (IC_50_=17.09 µM) which was slightly less than cisplatin.

Moreover, STAT3 and STAT5a inhibitory activities were determined for the five later compounds. Compounds **9d** and **9n** showed remarkable inhibitory activity against STAT3, while, compounds **9a**, **9f**, **9n**, and **9r** were the most effective at inhibiting STAT5a. Dual inhibitory activity at STAT3 and STAT5a was observed in compound **9n** which beared *p*-nitrophenyl and *p*-chlorophenyl rings at pyrimidine core in addition to dimethoxyphenyl at chalcone part. On the other hand, physicochemical properties, drug likeness scores, pharmacokinetics and toxicity properties were predicted for all the synthesised compounds **9a–r**.

## Supplementary Material

Supplemental Material
