# Supplementary material for: 2-Thiopyrimidine/chalcone hybrids: design, synthesis, ADMET prediction, and anticancer evaluation as STAT3/STAT5a inhibitors
Source: J Enzyme Inhib Med Chem. 2020 Mar 25;35(1):864–79. doi: 10.1080/14756366.2020.1740922 (PMC7144330; doi:10.1080/14756366.2020.1740922)
Supplement: Supplemental Material [file IENZ_A_1740922_SM0859.pdf]

^{1}H NMR 9a

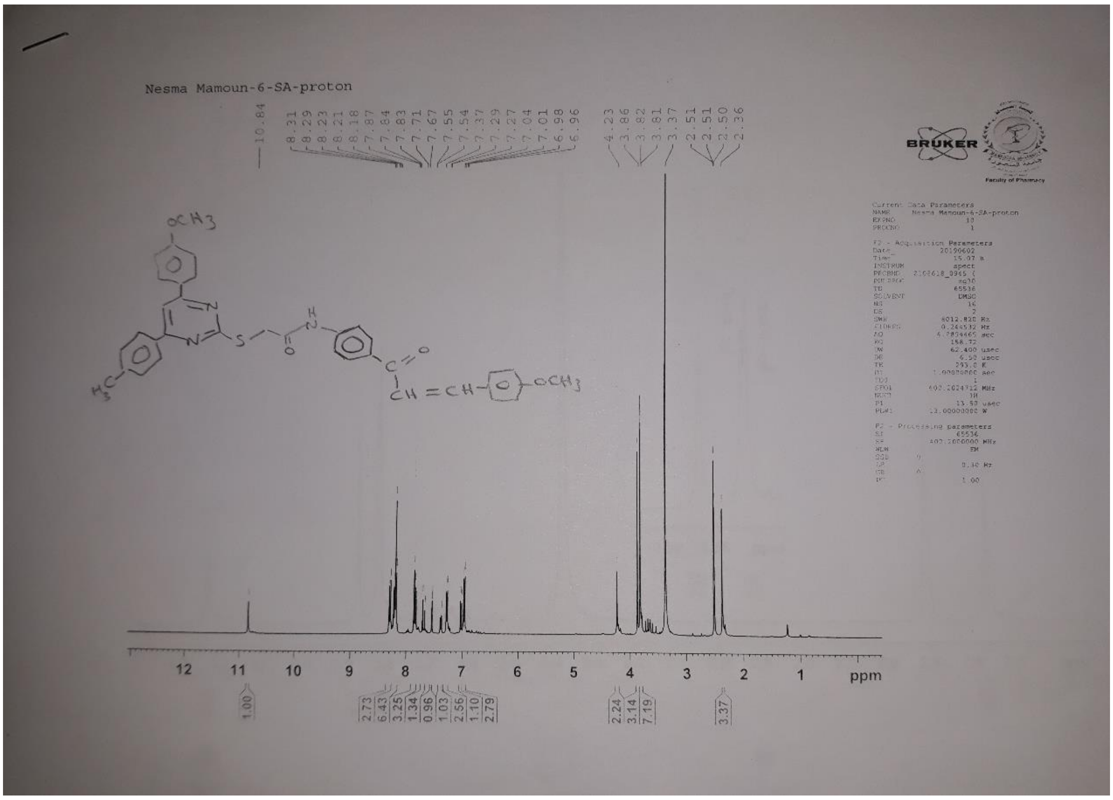

Nesma Mamoun-6-SA-proton
10.84
8.31 8.29 8.23 8.21 8.18 7.87 7.84 7.83 7.71 7.67 7.55 7.54 7.37 7.29 7.27 7.04 7.01 6.98 6.96
4.23 3.86 3.82 3.81 3.37 2.51 2.51 2.50 2.36
BRUKER
Faculty of Pharmacy
12 11 10 9 8 7 6 5 4 3 2 1 ppm
1.00
2.73 6.43 3.25 1.34 0.96 1.03 2.56 1.10 2.79
2.24 3.14 7.19
3.37

^{13}C NMR 9a

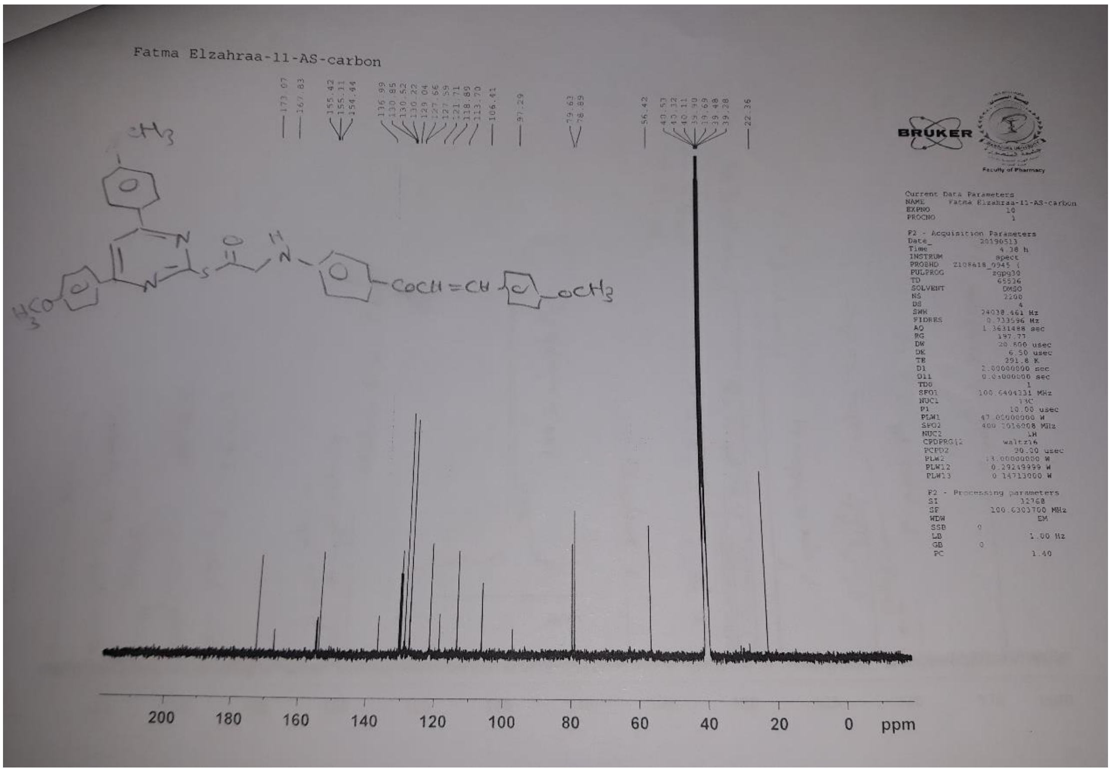

Fatma Elzahraa-11-AS-carbon
BRUKER
200
180
160
140
120
100
80
60
40
20
0
ppm

^{1}H NMR 9b

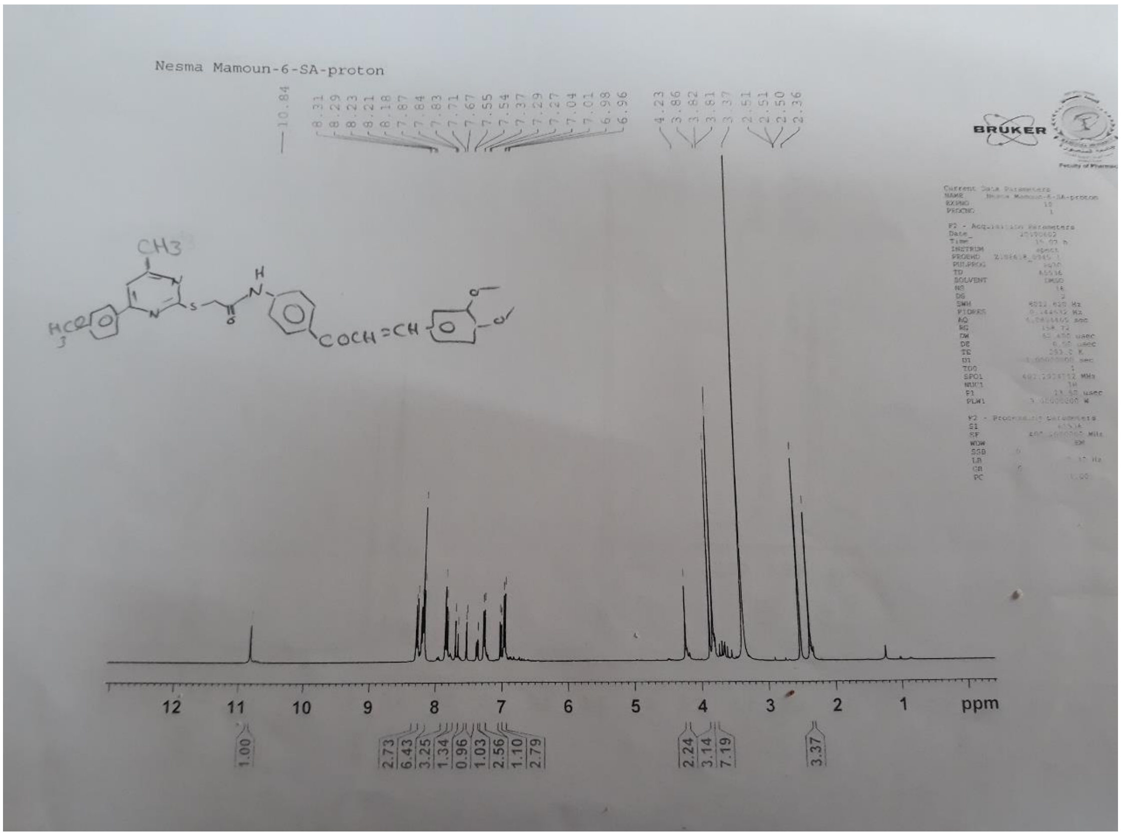
Nesma Mamoun-6-SA-proton
10.84
8.31
8.29
8.23
8.21
8.18
7.87
7.84
7.83
7.71
7.67
7.55
7.54
7.37
7.29
7.27
7.04
7.01
6.98
6.96
4.23
3.86
3.82
3.81
3.37
2.51
2.50
2.36
BRUKER
12
11
10
9
8
7
6
5
4
3
2
1
ppm
1.00
2.73
6.43
3.25
1.34
0.96
1.03
2.56
1.10
2.79
2.24
3.14
7.19
3.37

^{13}C NMR 9b

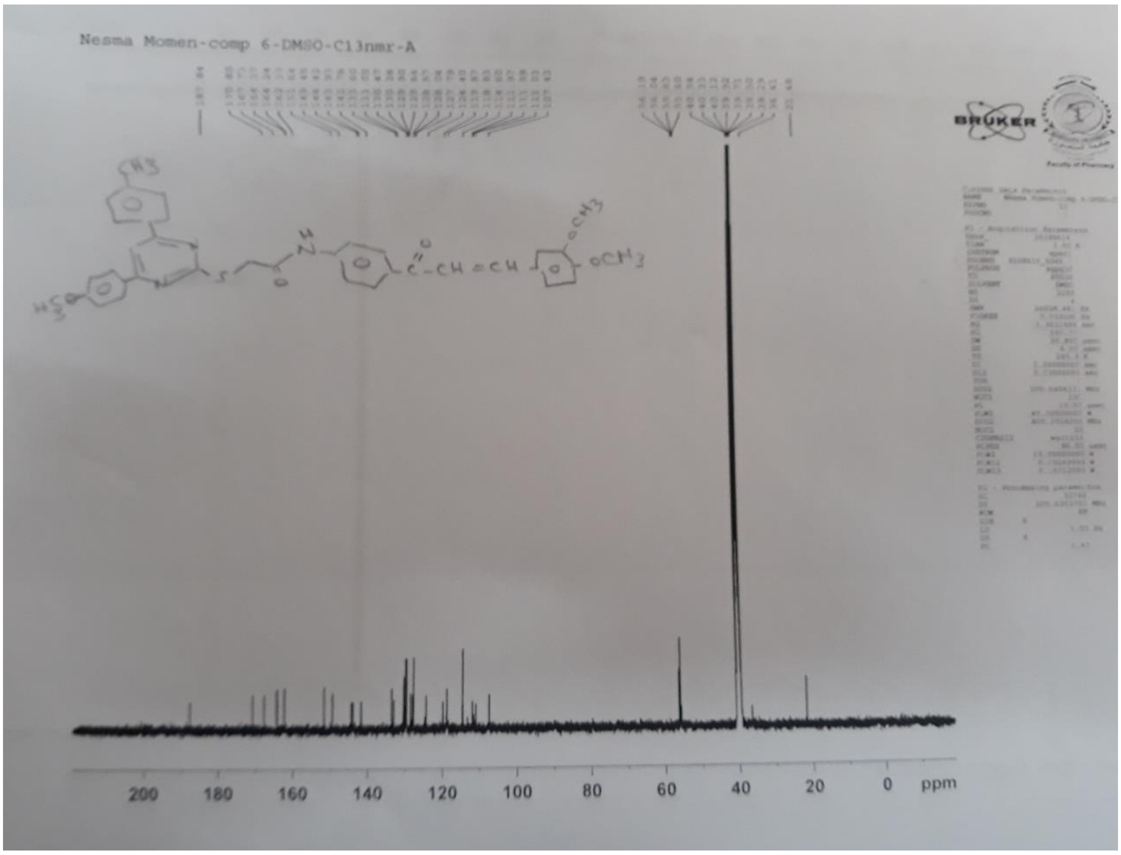

Nesma Momen-comp 6-DMSO-C13nmr-A
BRUKER
200
180
160
140
120
100
80
60
40
20
0
ppm

^{1}H NMR 9c

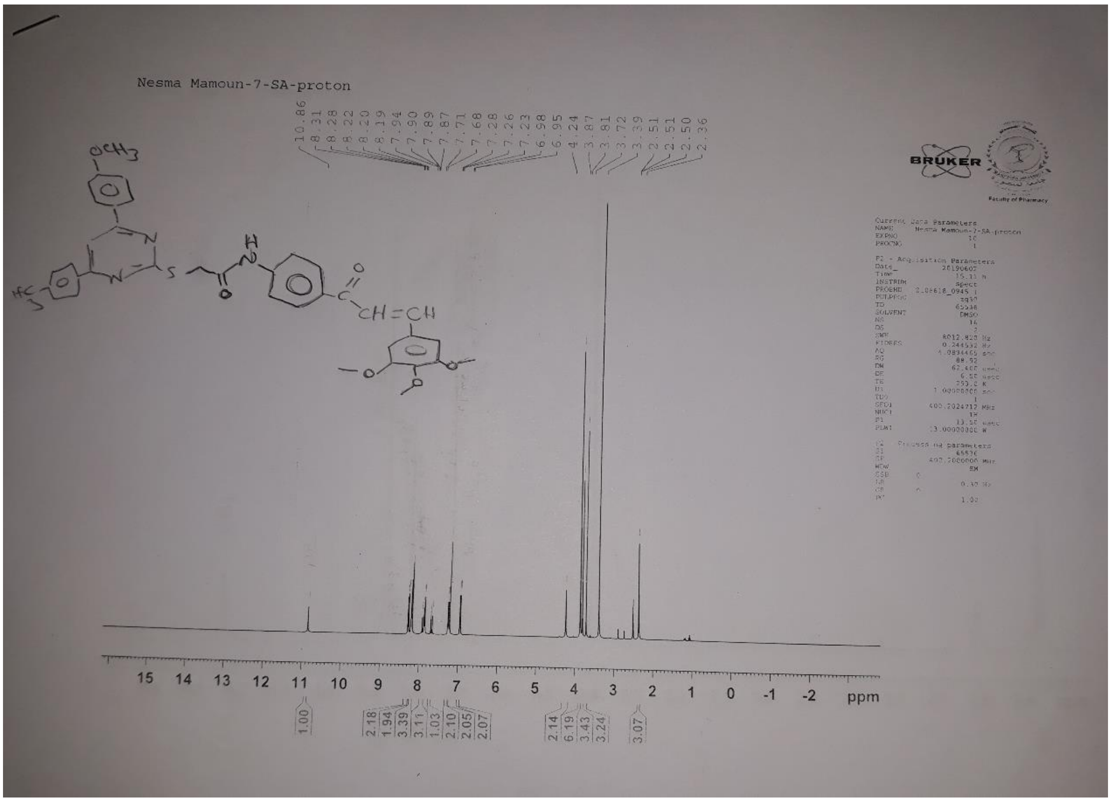

Nesma Mamoun-7-SA-proton
10.86
8.31
8.28
8.22
8.20
8.19
7.94
7.90
7.89
7.87
7.71
7.68
7.28
7.26
7.23
6.98
6.95
4.24
3.87
3.81
3.72
3.39
2.51
2.51
2.50
2.36
BRUKER
15 14 13 12 11 10 9 8 7 6 5 4 3 2 1 0 -1 -2 ppm
1.00
2.18
1.94
3.39
3.11
1.03
2.10
2.05
2.07
2.14
6.19
3.43
3.24
3.07

<u>^{13}C NMR 9c</u>

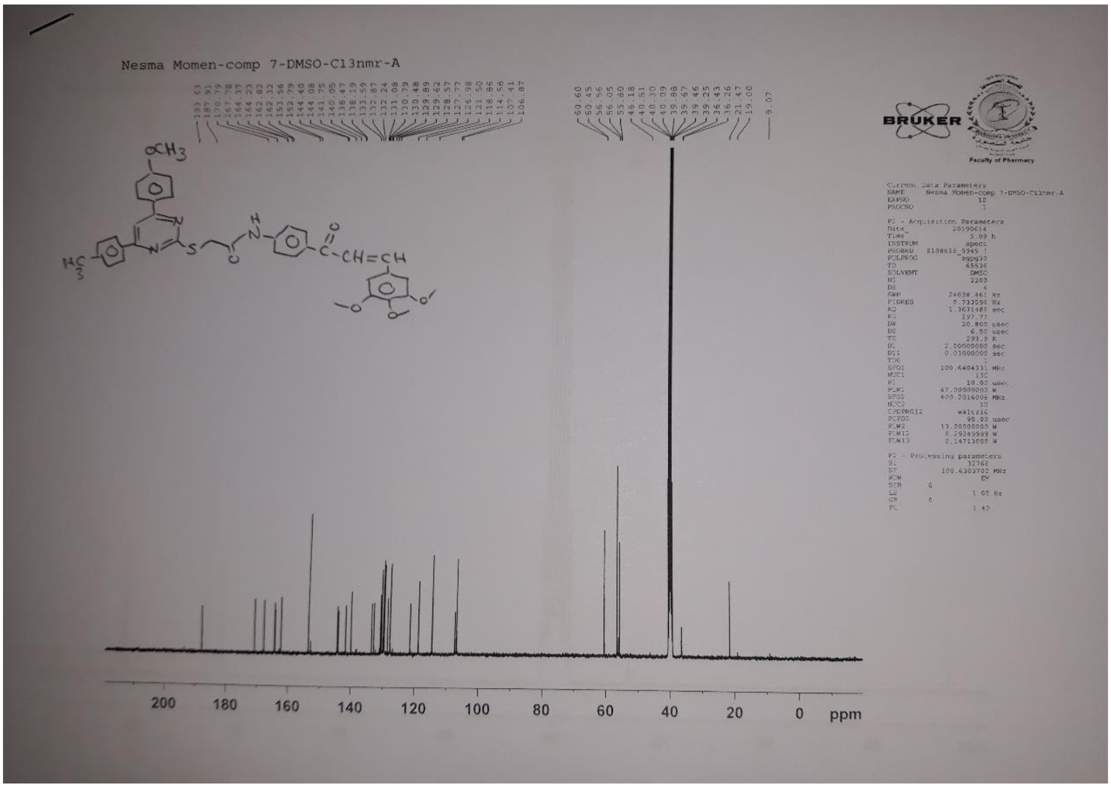
Nesma Momen-comp 7-DMSO-C13nmr-A
BRUKER
Faculty of Pharmacy
200 180 160 140 120 100 80 60 40 20 0 ppm

^{1}H NMR 9d

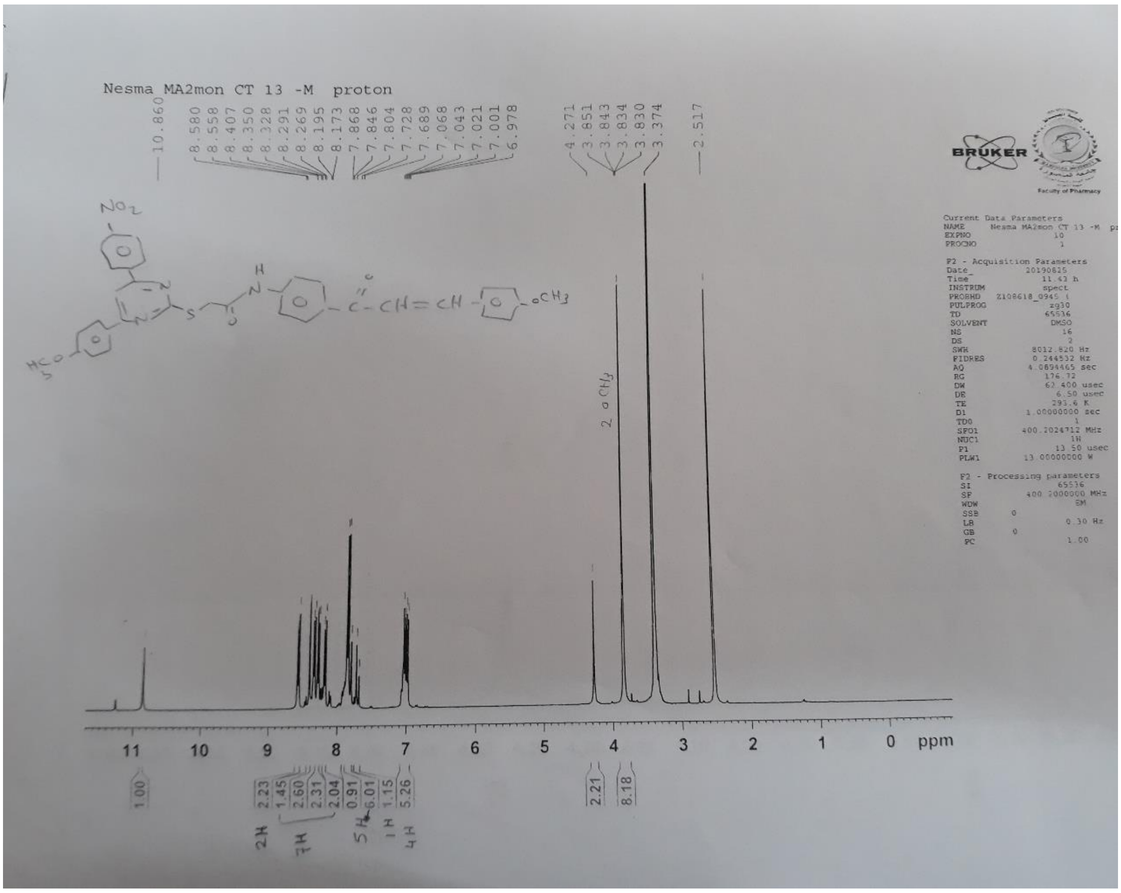

Nesma MA2mon CT 13 -M proton
10.860
8.580 8.558 8.407 8.350 8.328 8.291 8.269 8.195 8.173 7.868 7.846 7.804 7.728 7.689 7.068 7.043 7.021 7.001 6.978
4.271 3.851 3.843 3.834 3.830 3.374
2.517
BRUKER
Faculty of Pharmacy
Current Data Parameters
NAME Nesma MA2mon CT 13 -M
EXPNO 10
PROCNO 1
F2 - Acquisition Parameters
Date_ 20190825
Time 11.43 h
INSTRUM spect
PULPROG zg30
TD 65536
SOLVENT DMSO
NS 16
DS 2
SWH 8012.820 Hz
FIDRES 0.244532 Hz
AQ 4.0894465 sec
RG 176.72
DW 62.400 usec
DE 6.50 usec
TE 293.6 K
D1 1.00000000 sec
TD0 1
SFO1 400.2024712 MHz
NUC1 1H
P1 13.50 usec
PLW1 13.00000000 W
F2 - Processing parameters
SI 65536
SF 400.2000000 MHz
WDW EM
SSB 0
LB 0.30 Hz
GB 0
PC 1.00
11 10 9 8 7 6 5 4 3 2 1 0 ppm
1.00 2.23 1.45 2.60 2.31 2.04 0.91 6.01 1.15 5.26 2.21 8.18

^{13}C NMR 9d

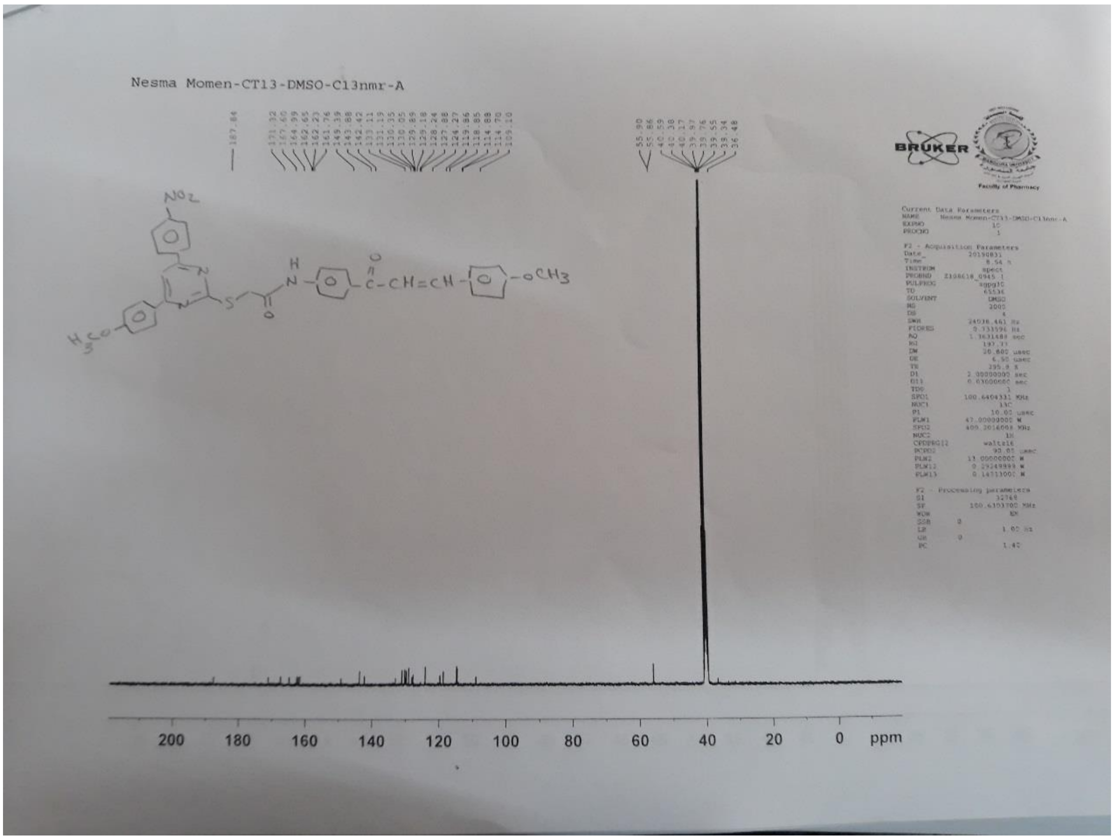

Nesma Momen-CT13-DMSO-C13nmr-A
200 180 160 140 120 100 80 60 40 20 0 ppm

^{1}H NMR 9e

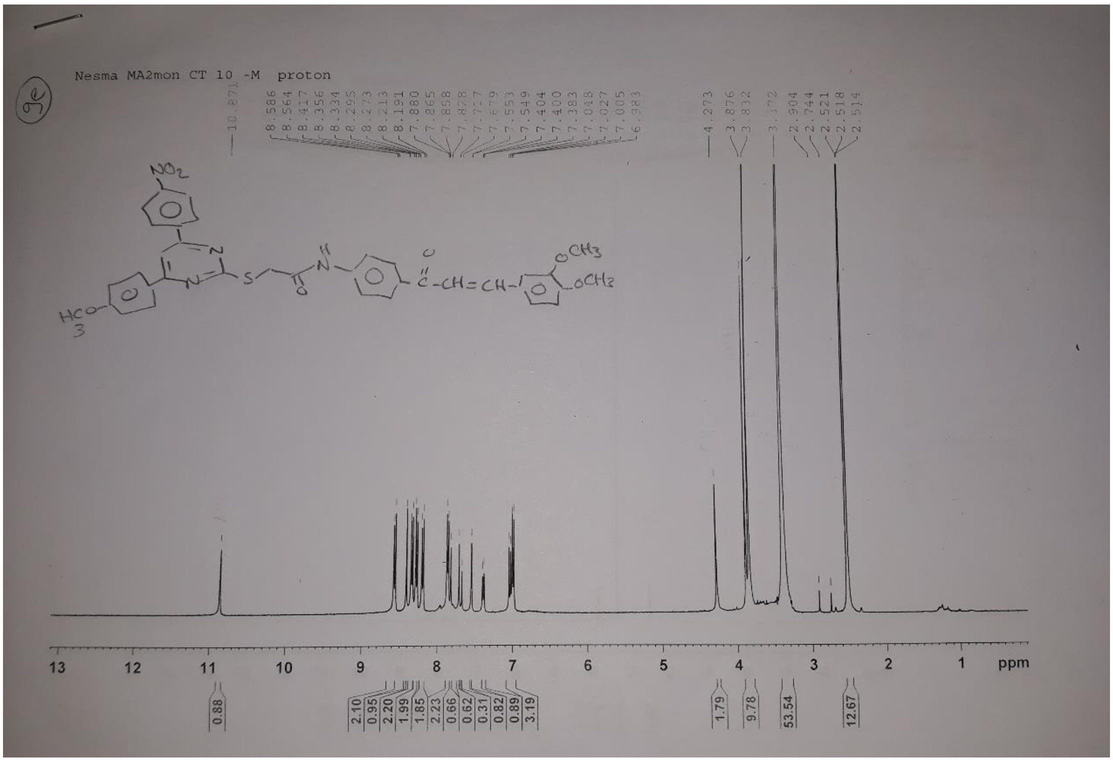

^{13}C NMR 9e

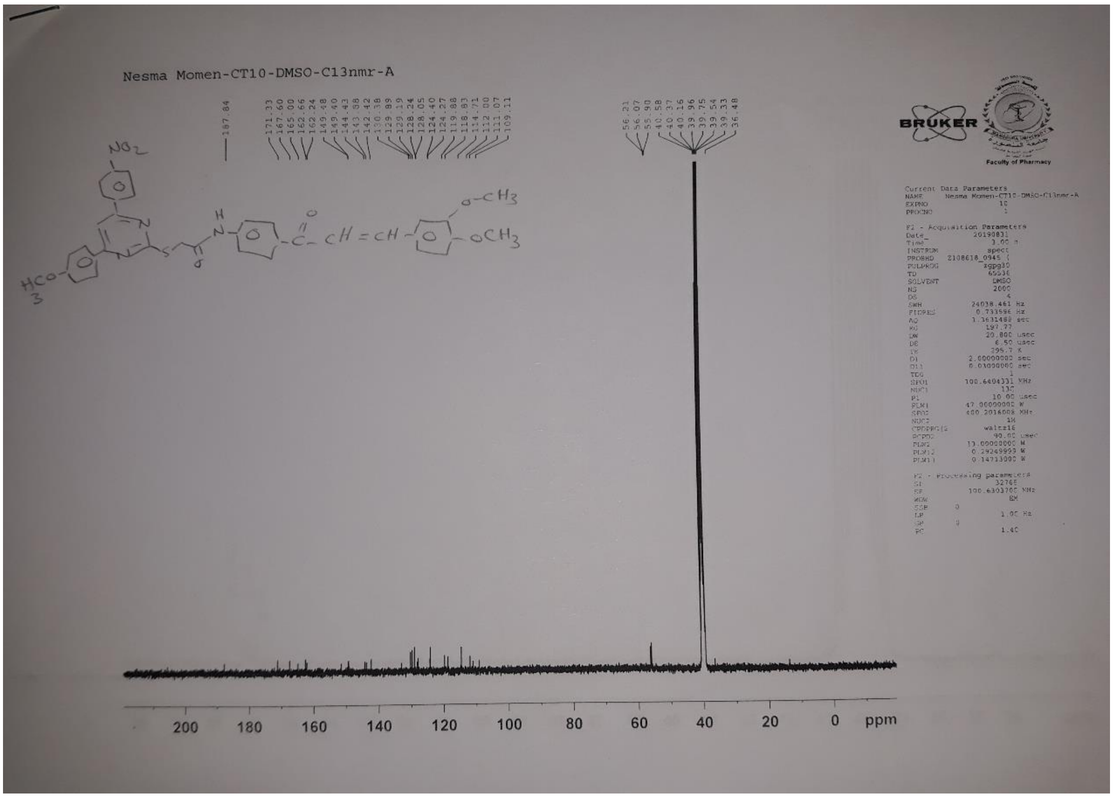

Nesma Momen-CT10-DMSO-C13nmr-A
BRUKER
Faculty of Pharmacy
ppm

^{1}H NMR 9f

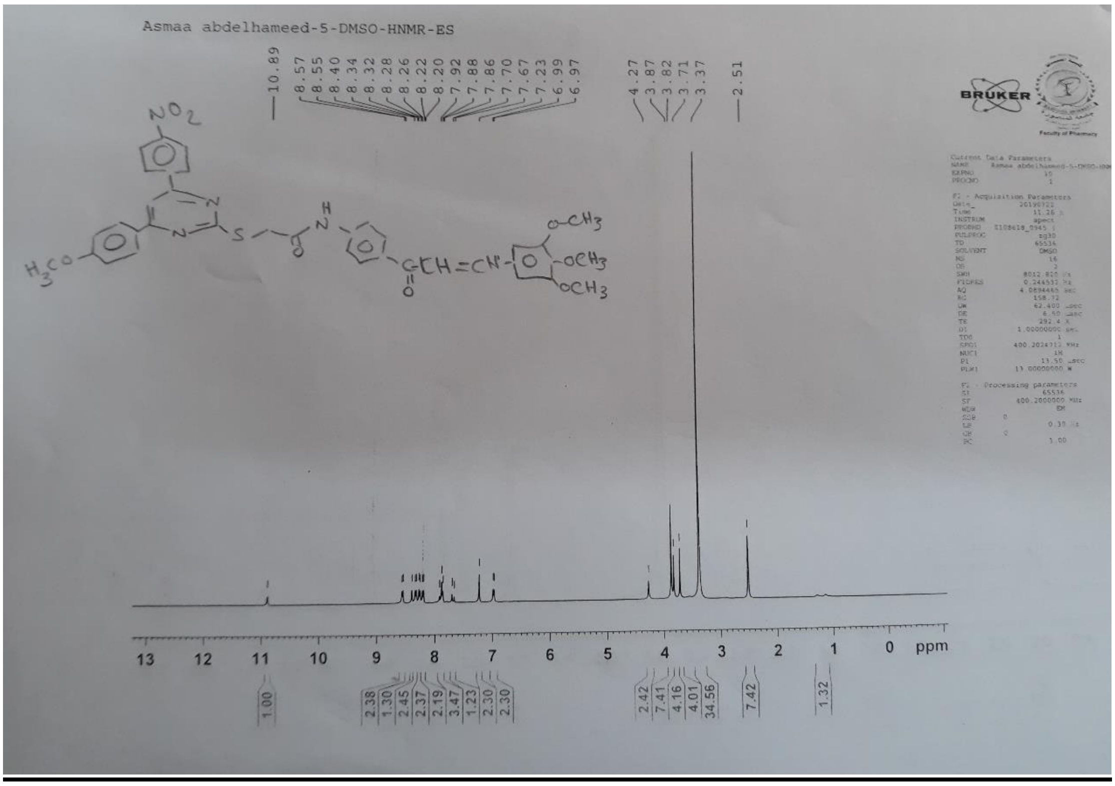

Asmaa abdelhameed-5-DMSO-HNMR-ES
10.89
8.57
8.55
8.40
8.34
8.32
8.28
8.26
8.22
8.20
7.92
7.88
7.86
7.70
7.67
7.23
6.99
6.97
4.27
3.87
3.82
3.71
3.37
2.51
BRUKER
ppm

^{13}C NMR 9f

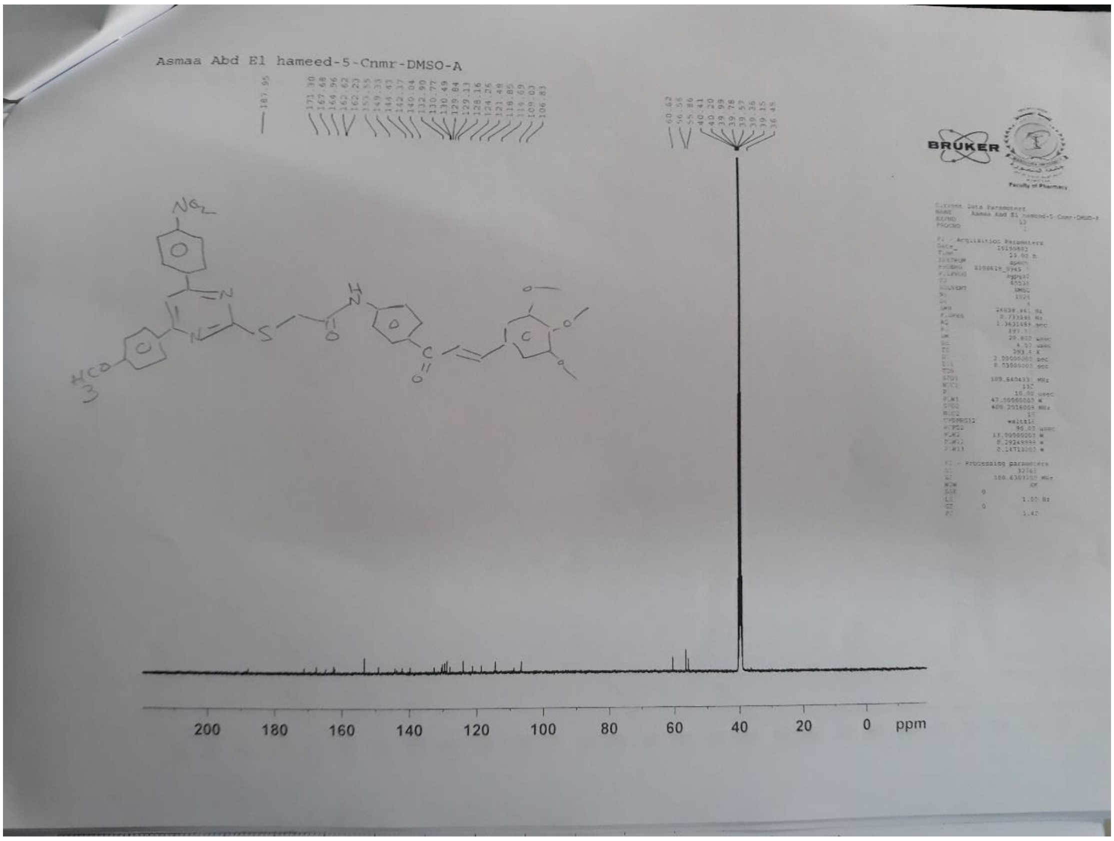

^{1}H NMR 9g

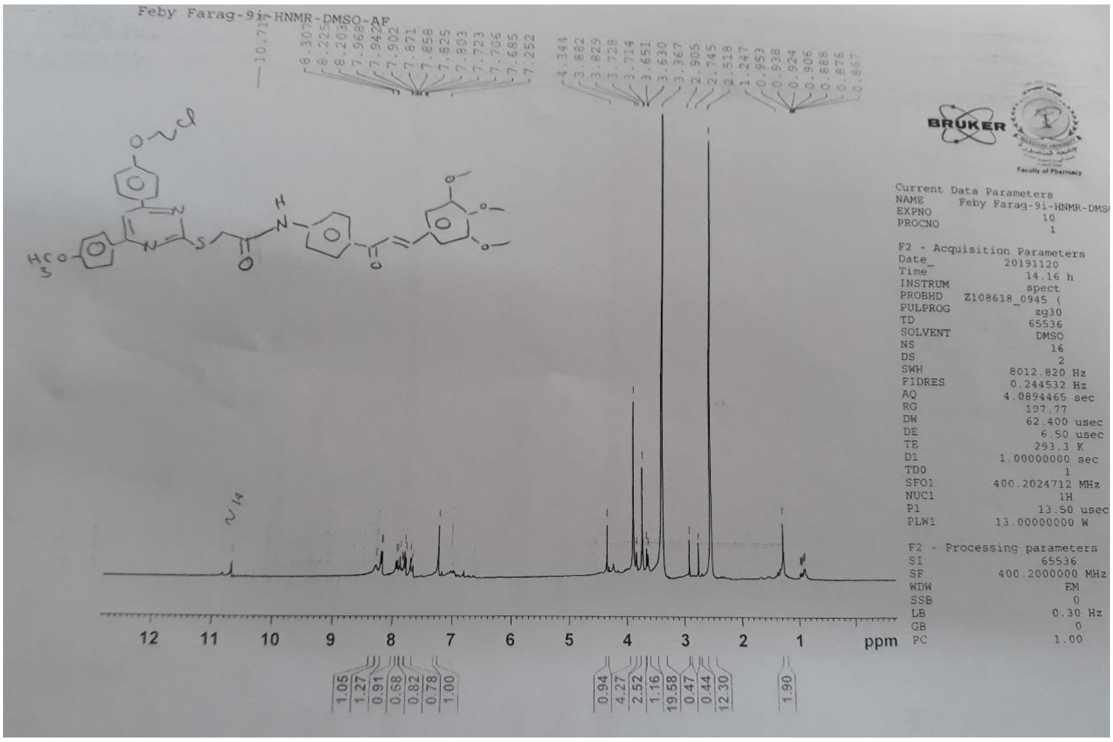

Feby Farag-9i-HNMR-DMSO-AF
Current Data Parameters
NAME Feby Farag-9i-HNMR-DMSO
EXPNO 10
PROCNO 1
F2 - Acquisition Parameters
Date_ 20191120
Time 14.16 h
INSTRUM spect
PROBHD Z108618_0945 (
PULPROG zg30
TD 65536
SOLVENT DMSO
NS 16
DS 2
SWH 8012.820 Hz
FIDRES 0.244532 Hz
AQ 4.0894465 sec
RG 197.77
DW 62.400 usec
DE 6.50 usec
TE 293.3 K
D1 1.00000000 sec
TD0 1
SFO1 400.2024712 MHz
NUC1 1H
P1 13.50 usec
PLW1 13.00000000 W
F2 - Processing parameters
SI 65536
SF 400.2000000 MHz
WDW EM
SSB 0
LB 0.30 Hz
GB 0
PC 1.00
ppm

^{13}C NMR 9g

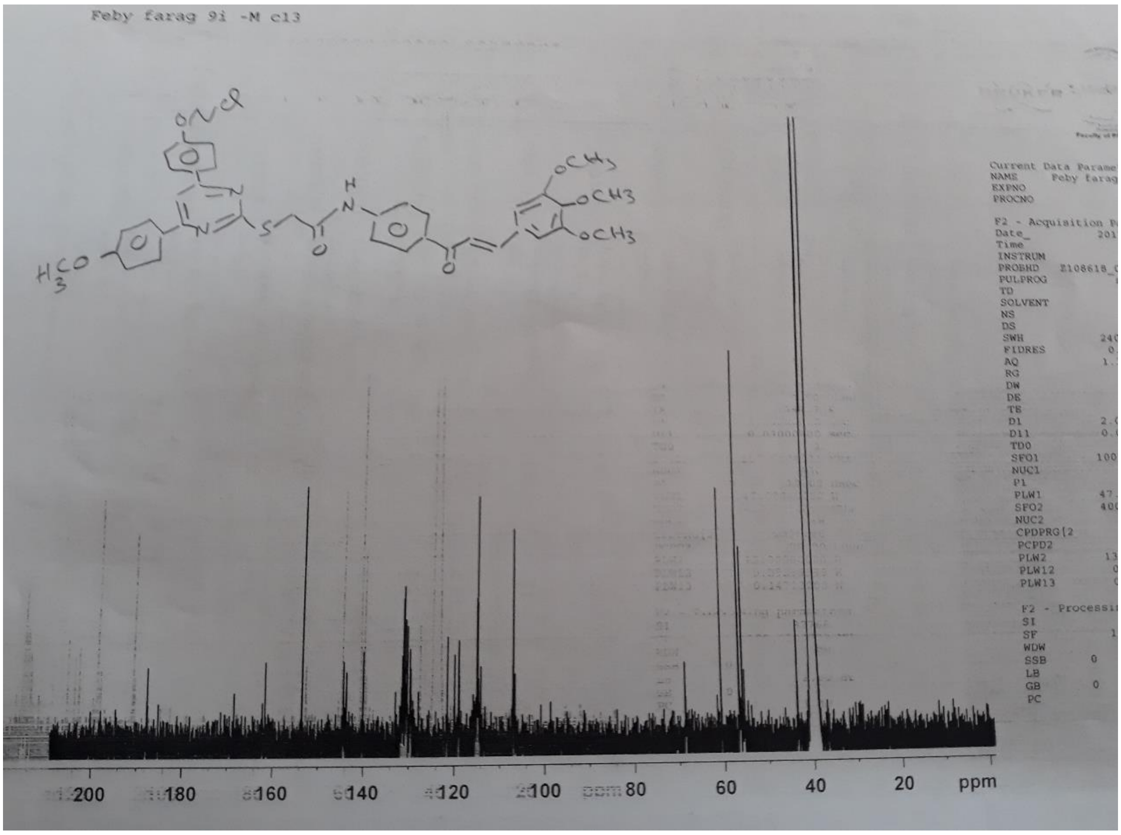
Feby farag 9i -M c13
Current Data Parame
NAME Feby farag
EXPNO
PROCNO
F2 - Acquisition P
Date_
Time
INSTRUM
PROBHD
PULPROG
TD
SOLVENT
NS
DS
SWH
FIDRES
AQ
RG
DW
DE
TE
D1
D11
TD0
SFO1
NUC1
P1
PLW1
SFO2
NUC2
CPDPRG[2
PCPD2
PLW2
PLW12
PLW13
F2 - Processi
SI
SF
WDW
SSB 0
LB
GB 0
PC
200
180
160
140
120
100
80
60
40
20
ppm

^{1}H NMR 9h

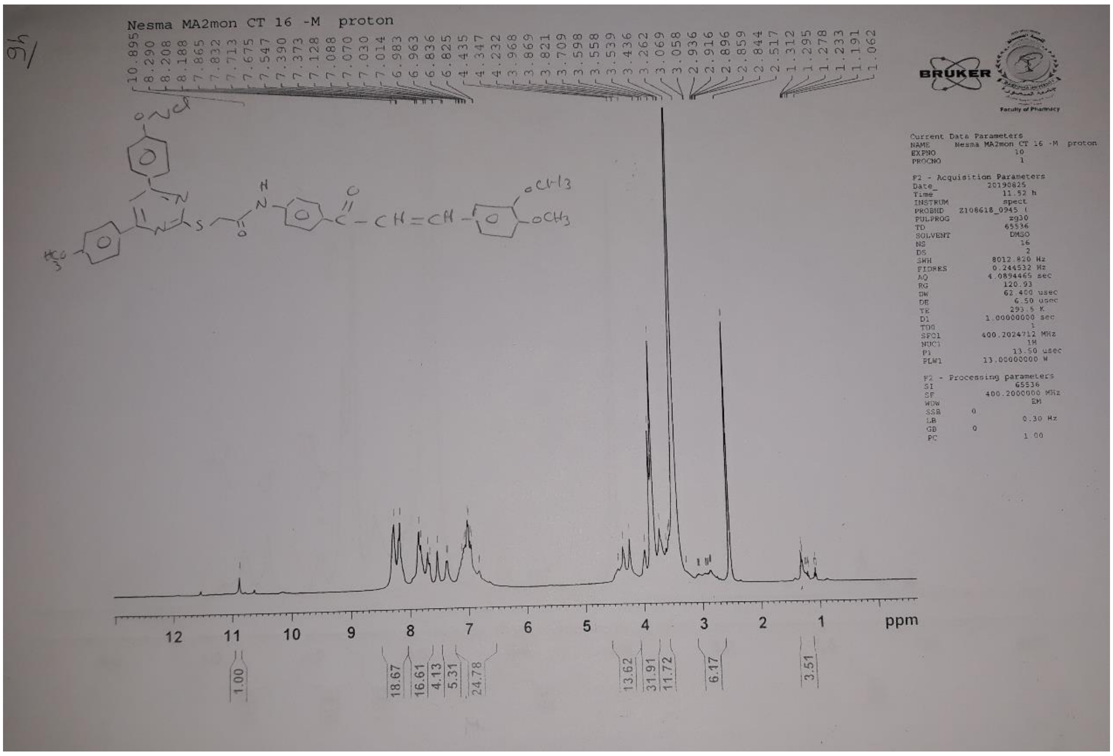

^{13}C NMR 9h

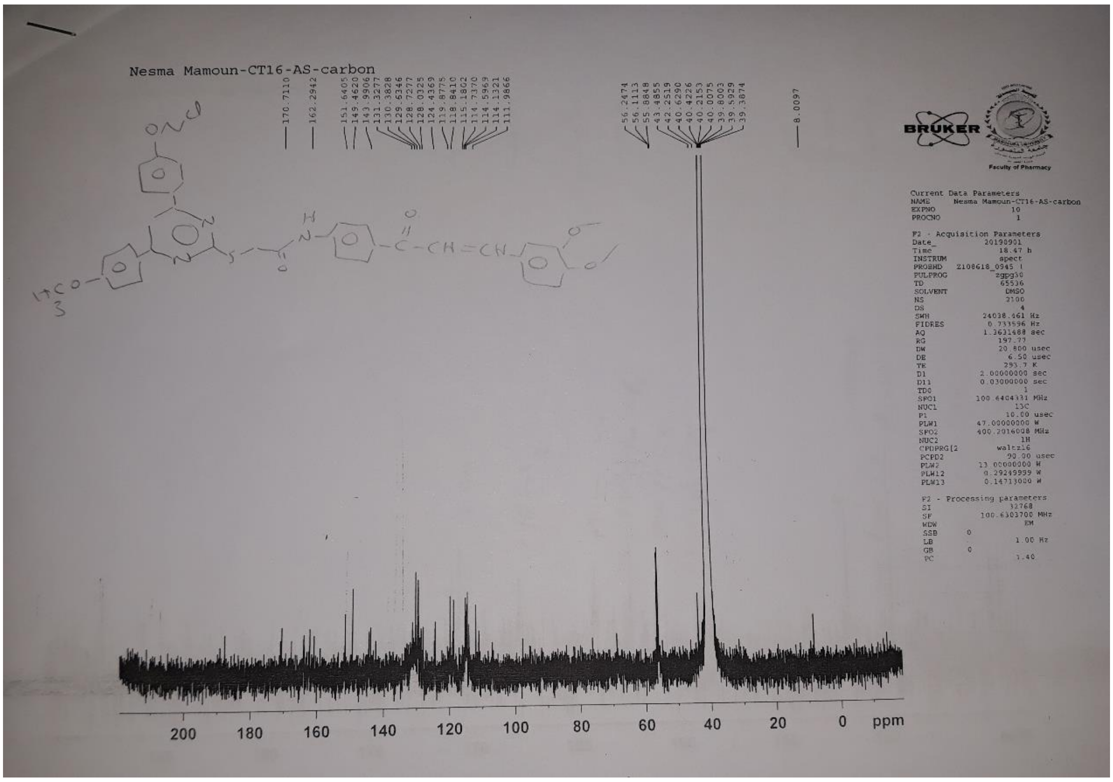

^{1}H NMR 9i

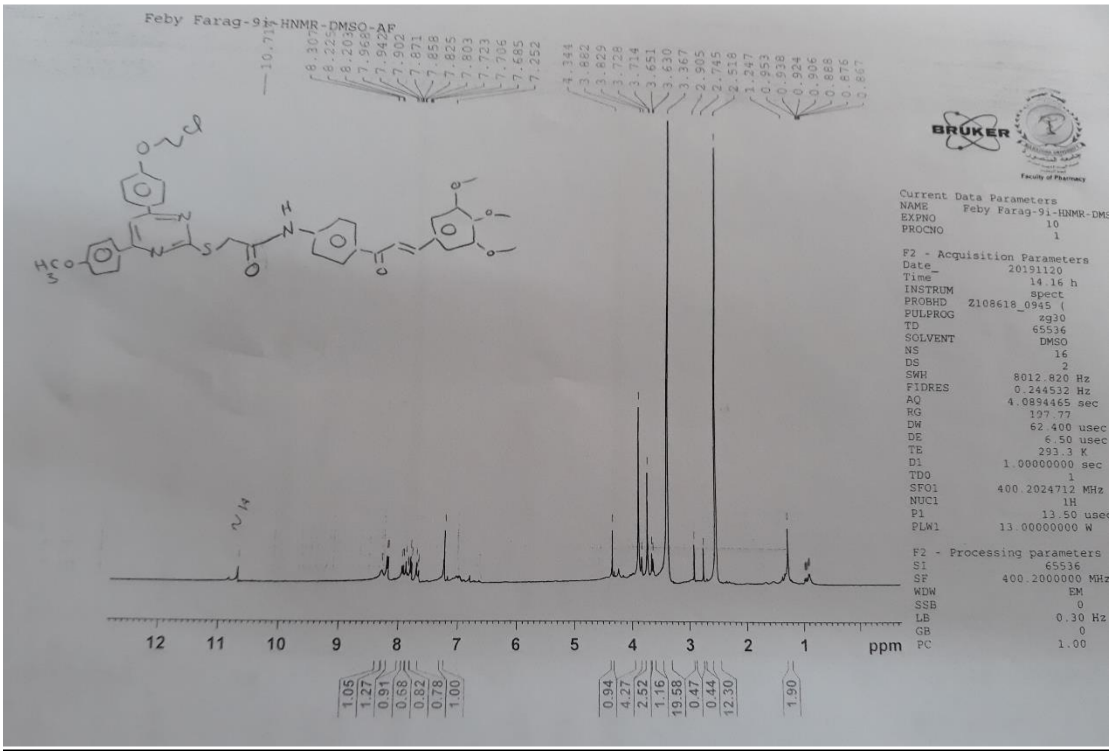

Feby Farag-9i-HNMR-DMSO-AF
Current Data Parameters
NAME Feby Farag-9i-HNMR-DMS
EXPNO 10
PROCNO 1
F2 - Acquisition Parameters
Date_ 20191120
Time 14.16 h
INSTRUM spect
PROBHD Z108618_0945 (
PULPROG zg30
TD 65536
SOLVENT DMSO
NS 16
DS 2
SWH 8012.820 Hz
FIDRES 0.244532 Hz
AQ 4.0894465 sec
RG 197.77
DW 62.400 usec
DE 6.50 usec
TE 293.3 K
D1 1.00000000 sec
TD0 1
SFO1 400.2024712 MHz
NUC1 1H
P1 13.50 usec
PLW1 13.00000000 W
F2 - Processing parameters
SI 65536
SF 400.2000000 MHz
WDW EM
SSB 0
LB 0.30 Hz
GB 0
PC 1.00
ppm

^{13}C NMR 9i

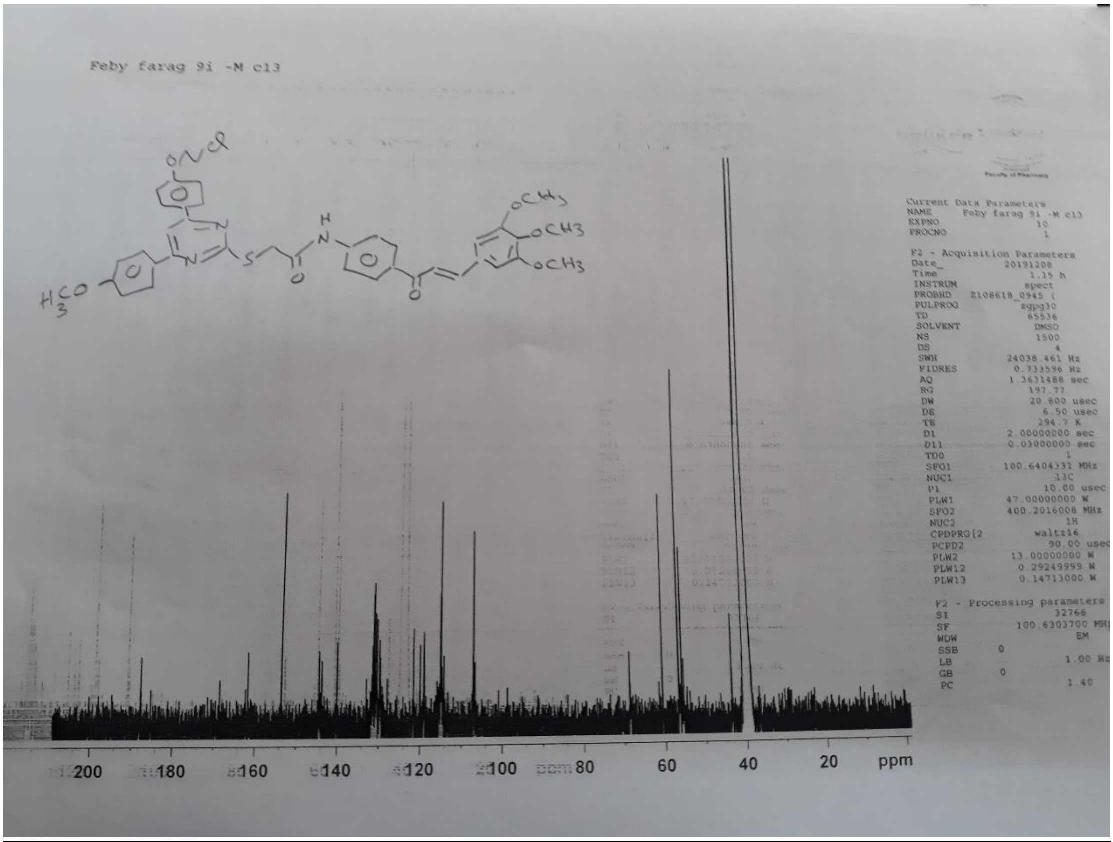
Feby farag 9i -M c13
Current Data Parameters
NAME Feby farag 9i -M c13
EXPNO 10
PROCNO 1
F2 - Acquisition Parameters
Date_ 20191208
Time 1.15 h
INSTRUM spect
PULPROG zgpg30
TD 65536
SOLVENT DMSO
NS 1500
DS 4
SWH 24038.461 Hz
FIDRES 0.733596 Hz
AQ 1.3631488 sec
RG 197.77
DW 20.800 usec
DE 6.50 usec
TE 294.7 K
D1 2.00000000 sec
D11 0.03000000 sec
TD0 1
SFO1 100.6404331 MHz
NUC1 13C
P1 10.00 usec
PLW1 47.00000000 W
SFO2 400.2016008 MHz
NUC2 1H
CPDPRG[2 waltz16
PCPD2 90.00 usec
PLW2 13.00000000 W
PLW12 0.29249999 W
PLW13 0.14713000 W
F2 - Processing parameters
SI 32768
SF 100.6303700 MHz
WDW EM
SSB 0
LB 1.00 Hz
GB 0
PC 1.40
200
180
160
140
120
100
80
60
40
20
ppm

^{1}H NMR 9j

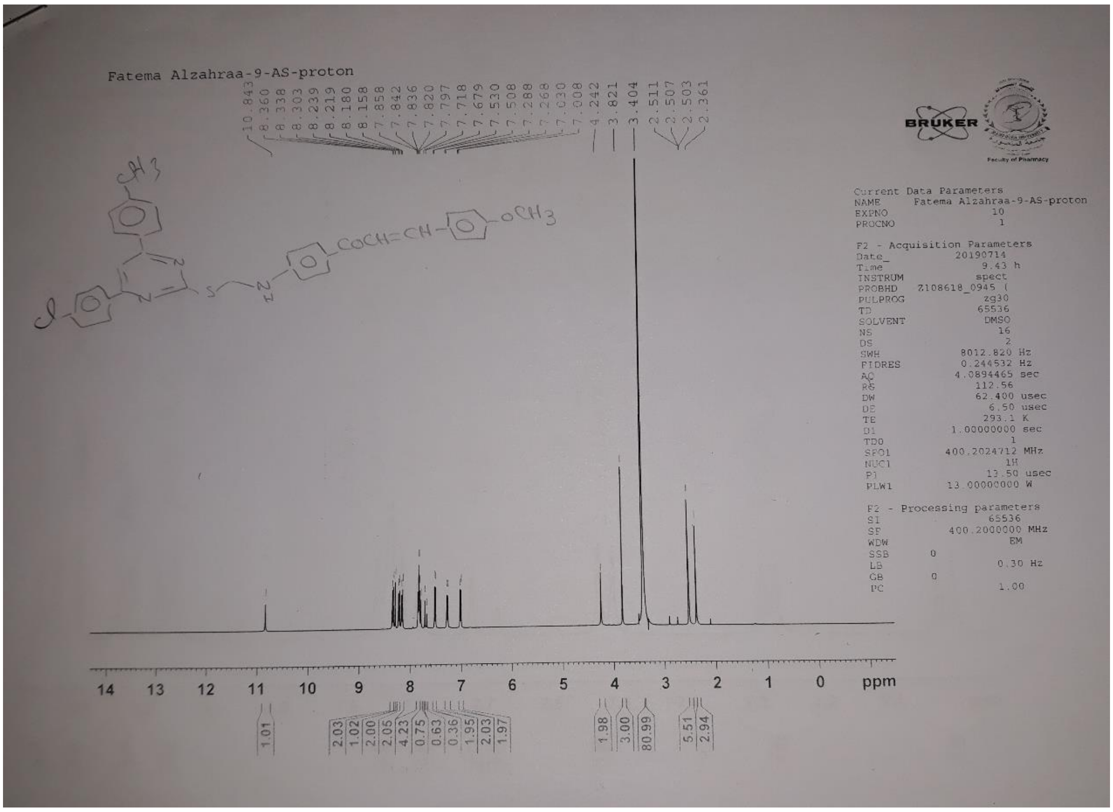
Fatema Alzahraa-9-AS-proton
10.843
8.360
8.338
8.303
8.239
8.219
8.180
8.158
7.858
7.842
7.836
7.820
7.797
7.718
7.679
7.530
7.508
7.288
7.268
7.030
7.008
4.242
3.821
3.404
2.511
2.507
2.503
2.361
BRUKER
Current Data Parameters
NAME Fatema Alzahraa-9-AS-proton
EXPNO 10
PROCNO 1
F2 - Acquisition Parameters
Date_ 20190714
Time 9.43 h
INSTRUM spect
PULPROG zg30
TD 65536
SOLVENT DMSO
NS 16
DS 2
SWH 8012.820 Hz
FIDRES 0.244532 Hz
AQ 4.0894465 sec
RG 112.56
DW 62.400 usec
DE 6.50 usec
TE 293.1 K
D1 1.00000000 sec
TD0 1
SFO1 400.2024712 MHz
NUC1 1H
P1 13.50 usec
PLW1 13.00000000 W
F2 - Processing parameters
SI 65536
SF 400.2000000 MHz
WDW EM
SSB 0
LB 0.30 Hz
GB 0
PC 1.00
14 13 12 11 10 9 8 7 6 5 4 3 2 1 0 ppm
1.01
2.03
1.02
2.00
2.05
4.23
0.75
0.63
0.36
1.95
2.03
1.97
1.98
3.00
80.99
5.51
2.94

^{13}C NMR 9j

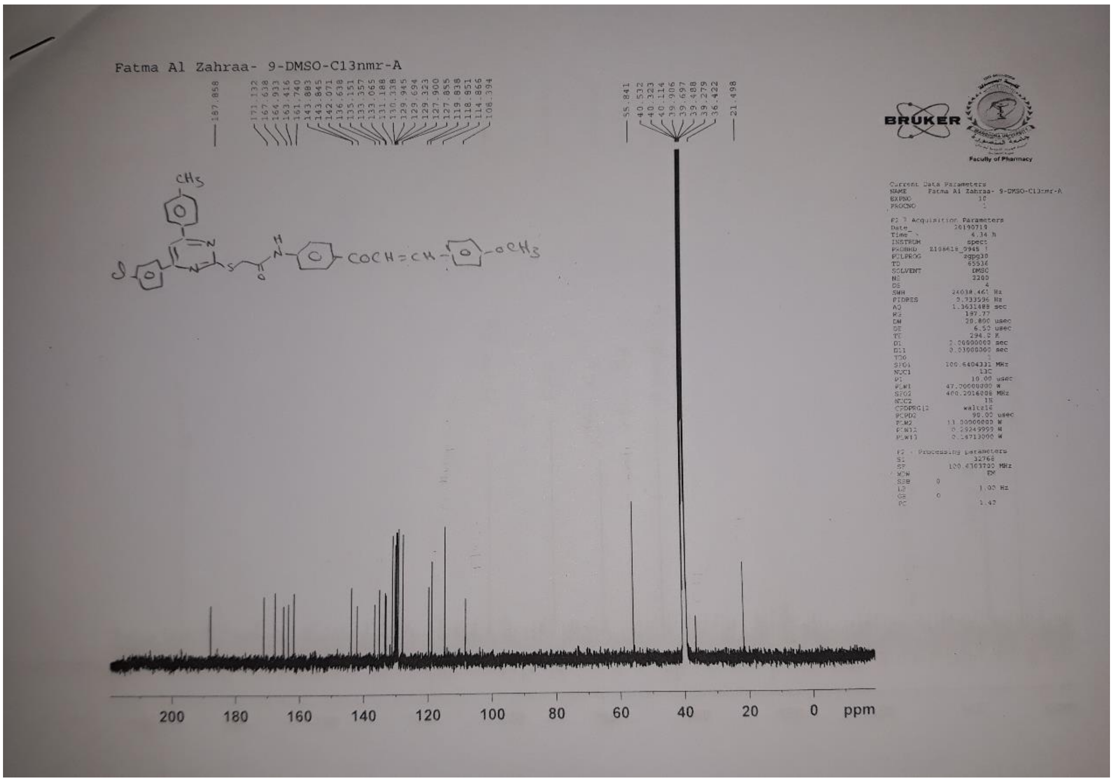

^{1}H NMR 9k

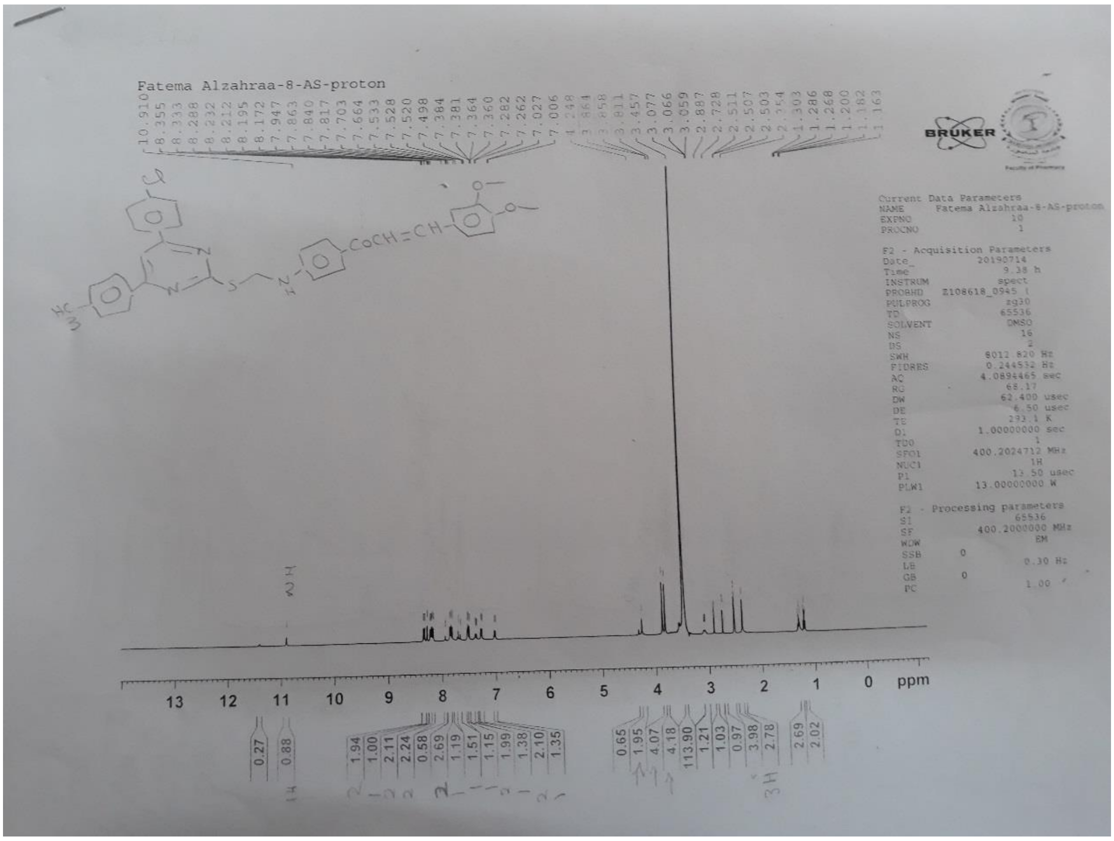

^{13}C NMR 9k

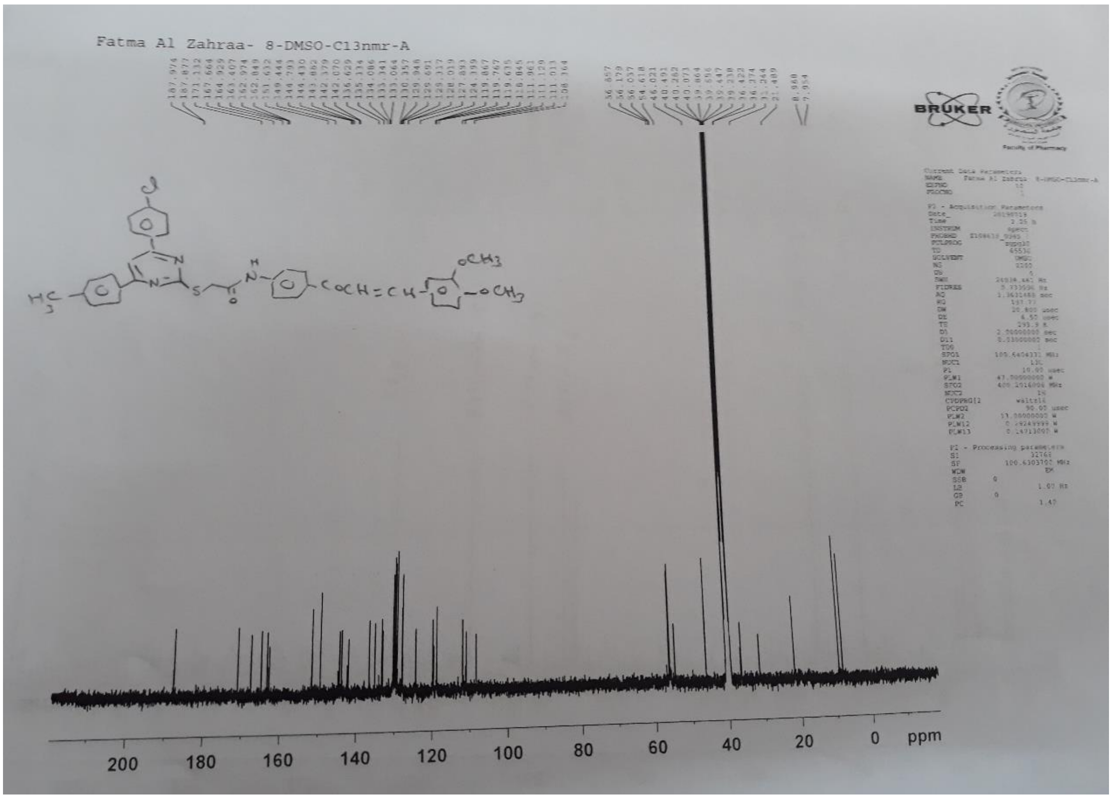

Fatma Al Zahraa- 8-DMSO-C13nmr-A
BRUKER
ppm
200
180
160
140
120
100
80
60
40
20
0

^{1}H NMR 9l

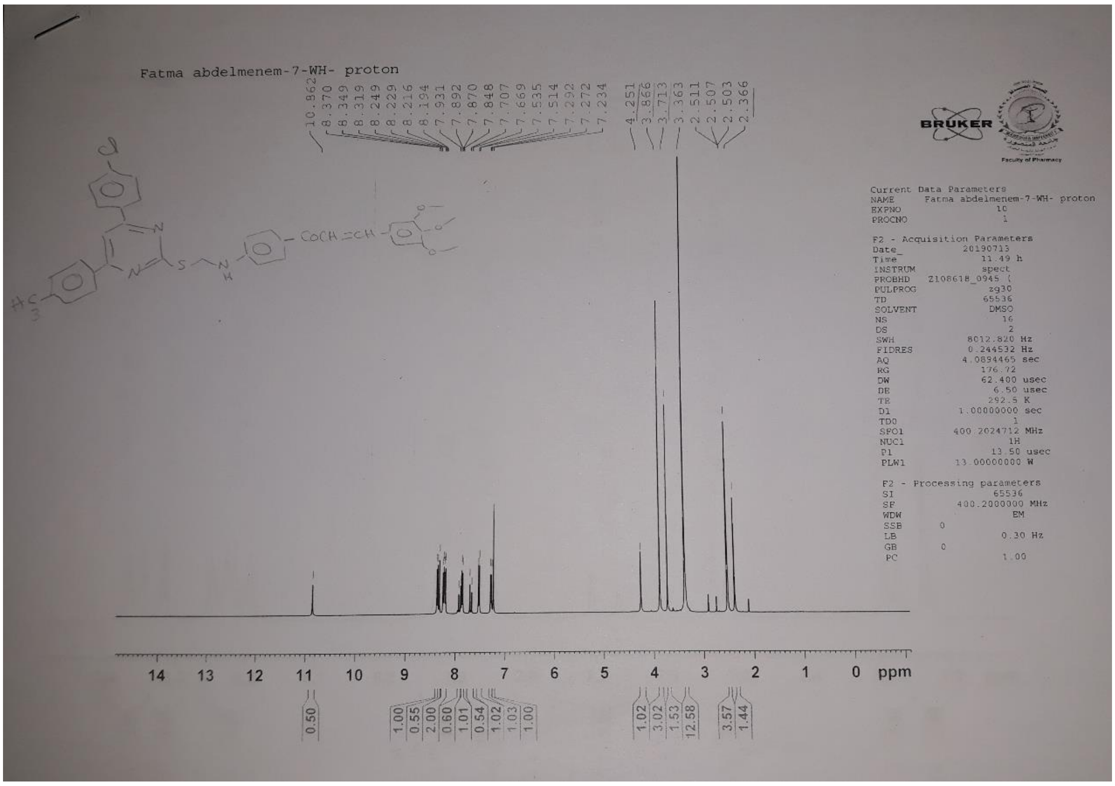

Fatma abdelmenem-7-WH- proton
10.862
8.370
8.349
8.319
8.249
8.229
8.216
8.194
7.931
7.892
7.870
7.848
7.707
7.669
7.535
7.514
7.292
7.272
7.234
4.251
3.866
3.713
3.363
2.511
2.507
2.503
2.366
BRUKER
Current Data Parameters
NAME Fatma abdelmenem-7-WH- proton
EXPNO 10
PROCNO 1
F2 - Acquisition Parameters
Date_ 20190715
Time 11.49 h
INSTRUM spect
PULPROG zg30
TD 65536
SOLVENT DMSO
NS 16
DS 2
SWH 8012.820 Hz
FIDRES 0.244532 Hz
AQ 4.0894465 sec
RG 176.72
DW 62.400 usec
DE 6.50 usec
TE 292.8 K
D1 1.00000000 sec
TD0 1
SFO1 400.2024712 MHz
NUC1 1H
P1 13.50 usec
PLW1 13.00000000 W
F2 - Processing parameters
SI 65536
SF 400.2000000 MHz
WDW EM
SSB 0
LB 0.30 Hz
GB 0
PC 1.00
14 13 12 11 10 9 8 7 6 5 4 3 2 1 0 ppm
0.50
1.00
0.55
2.00
0.60
1.01
0.54
1.02
1.03
1.00
1.02
3.02
1.53
12.58
3.57
1.44

^{13}C NMR 9l

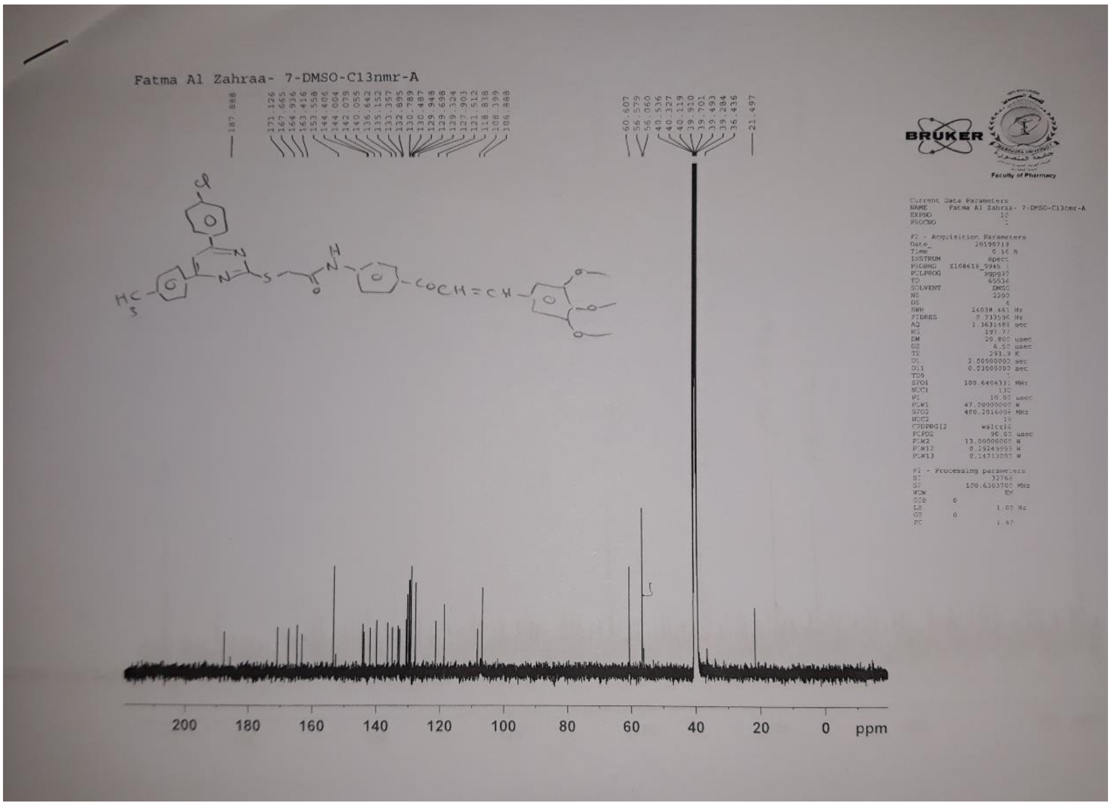

Fatma Al Zahraa- 7-DMSO-C13nmr-A
BRUKER
Faculty of Pharmacy
200
180
160
140
120
100
80
60
40
20
0
ppm

^{1}H NMR 9m

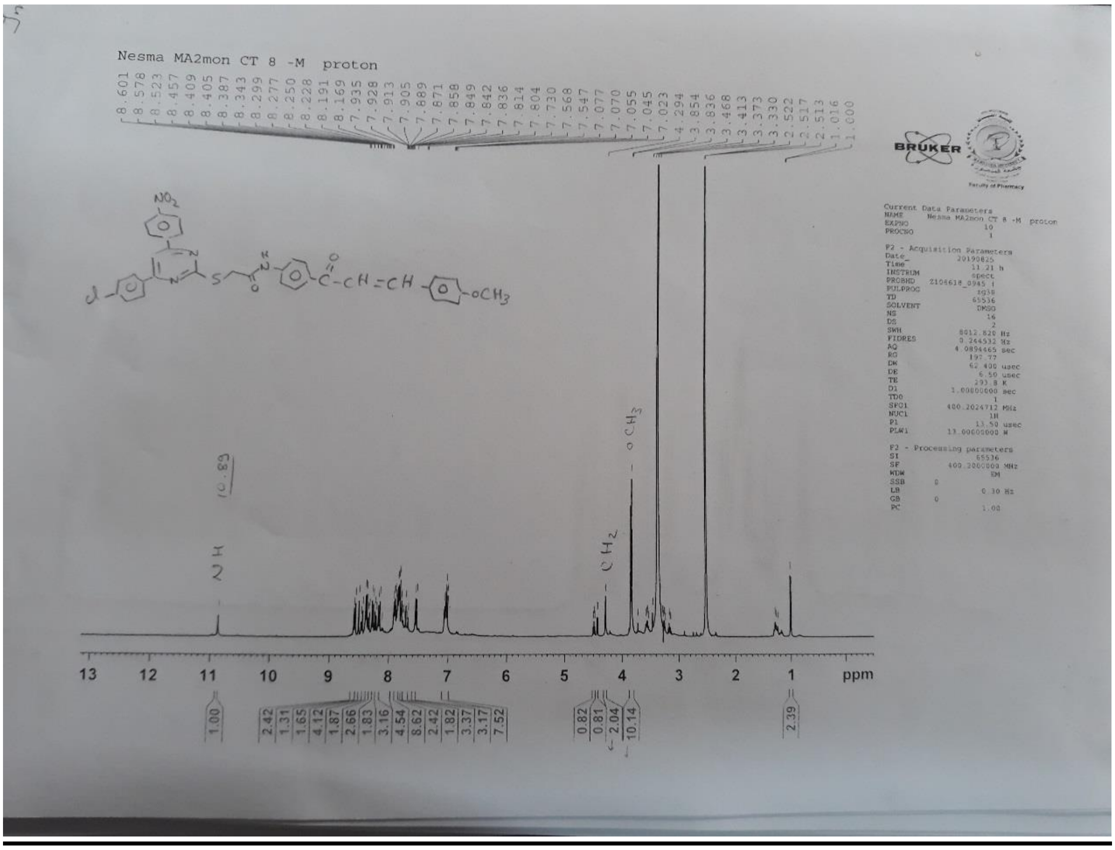

^{13}C NMR 9m

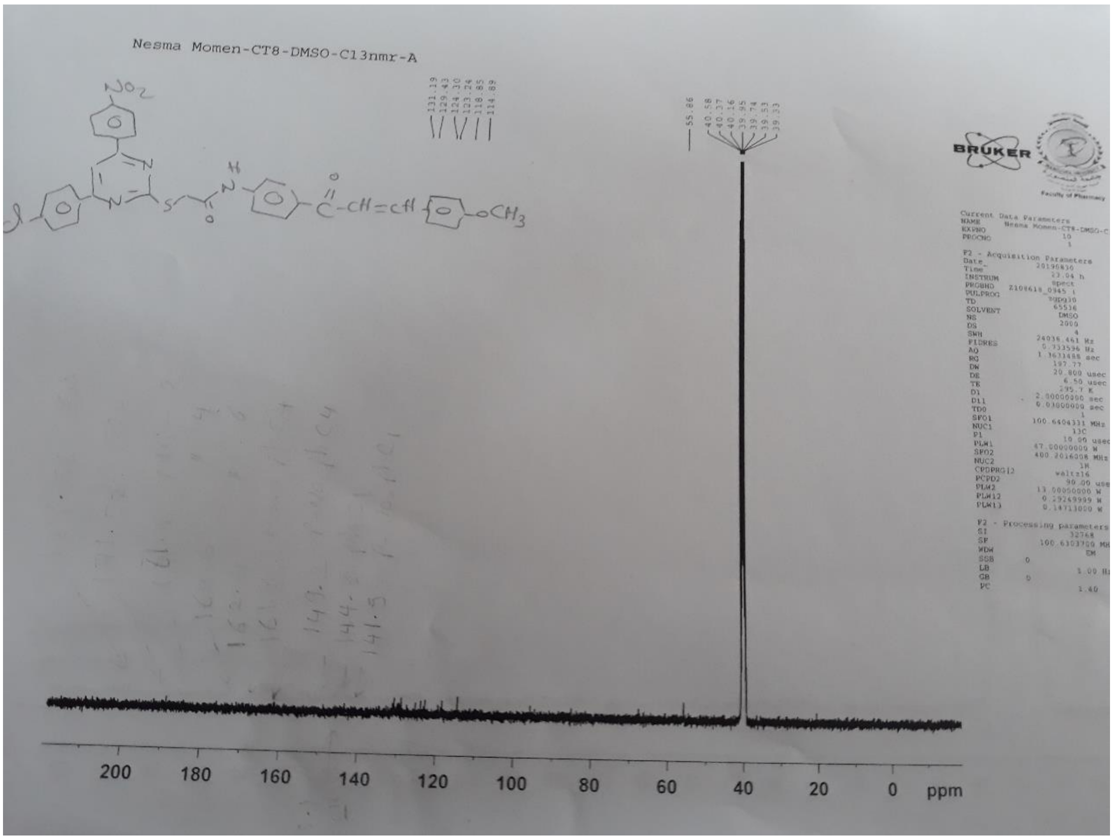
Nesma Momen-CT8-DMSO-C13nmr-A
200
180
160
140
120
100
80
60
40
20
0
ppm

^{1}H NMR 9n

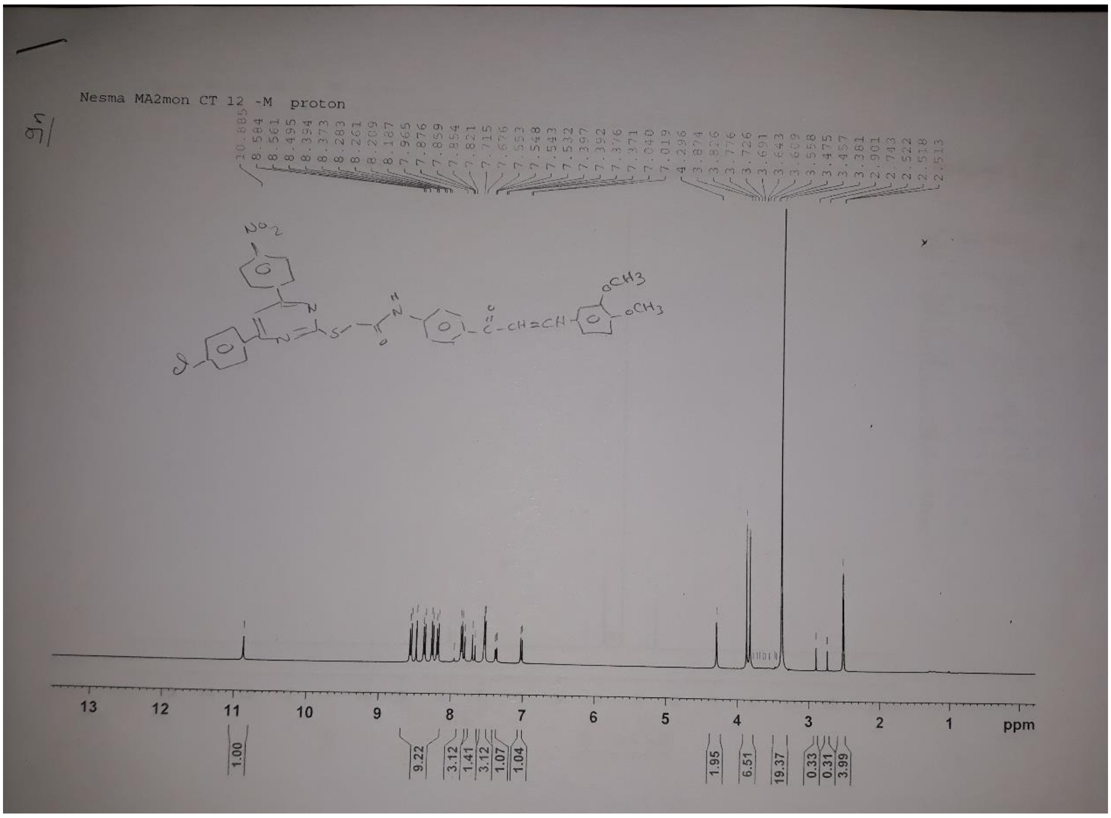

Nesma MA2mon CT 12 -M proton
13
12
11
10
9
8
7
6
5
4
3
2
1
ppm
1.00
9.22
3.12
1.41
3.12
1.07
1.04
1.95
6.51
19.37
0.33
0.31
3.99

^{13}C NMR 9n

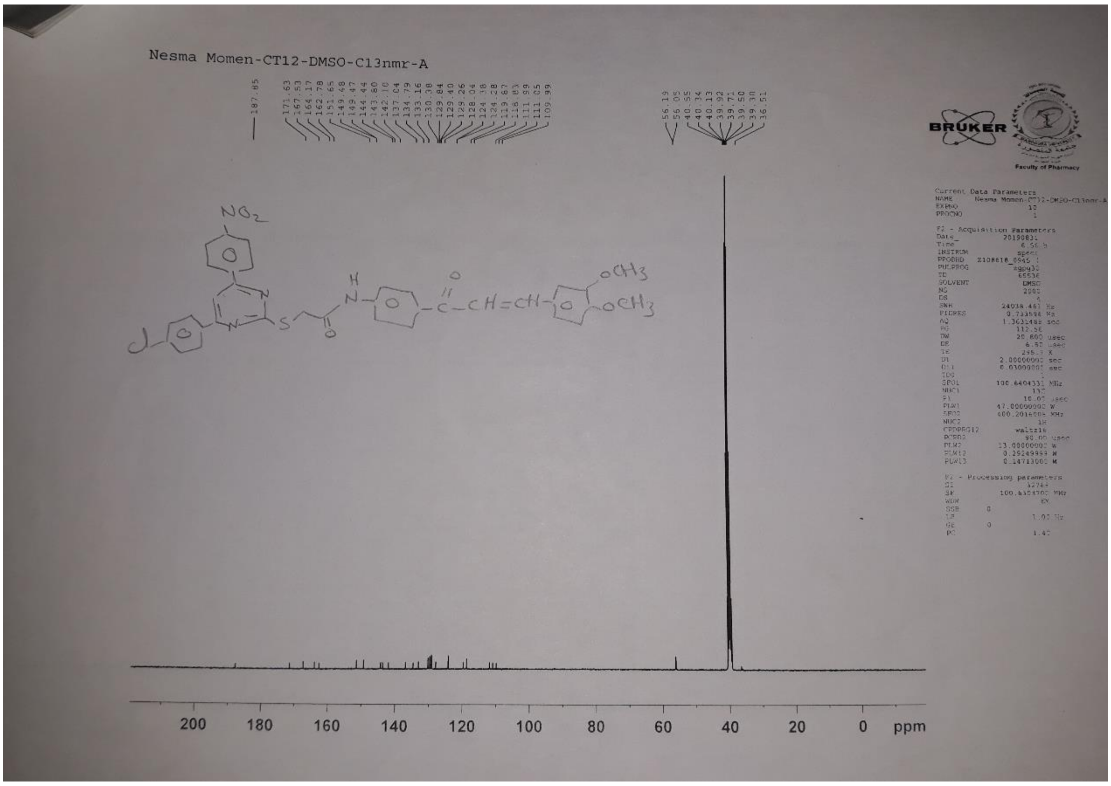

Nesma Momen-CT12-DMSO-C13nmr-A
BRUKER
Faculty of Pharmacy
200 180 160 140 120 100 80 60 40 20 0 ppm

^{1}H NMR 9o

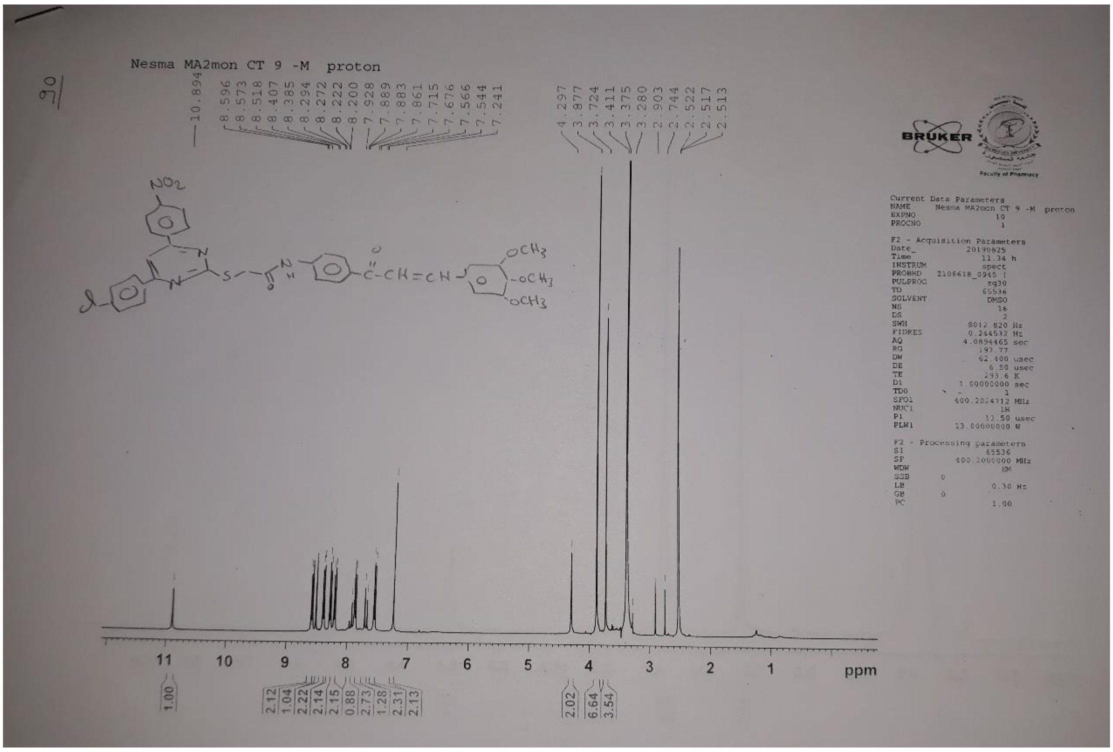

Nesma MA2mon CT 9 -M proton
10.894
8.596
8.573
8.518
8.407
8.385
8.294
8.272
8.222
8.200
7.928
7.889
7.883
7.861
7.715
7.676
7.566
7.544
7.241
4.297
3.877
3.724
3.411
3.375
3.280
2.903
2.744
2.522
2.517
2.513
BRUKER
Current Data Parameters
NAME Nesma MA2mon CT 9 -M proton
EXPNO 10
PROCNO 1
F2 - Acquisition Parameters
SOLVENT DMSO
F2 - Processing parameters
ppm

^{13}C NMR 9o

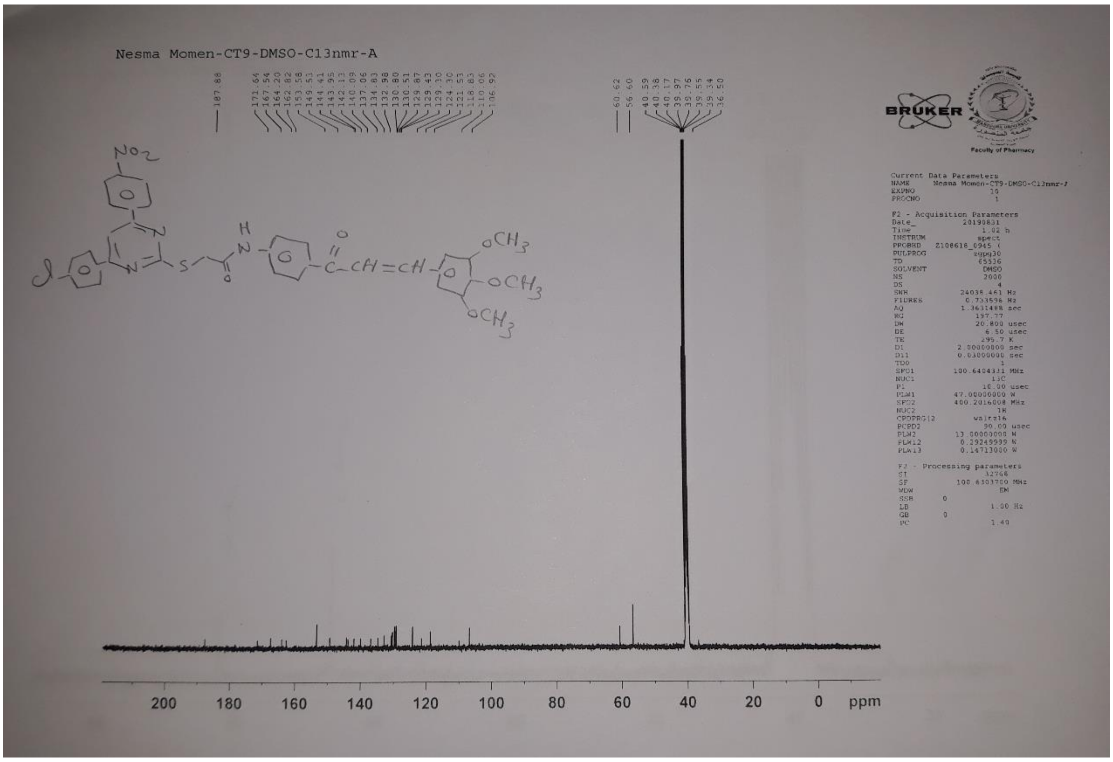
Nesma Momen-CT9-DMSO-C13nmr-A
BRUKER
200 180 160 140 120 100 80 60 40 20 0 ppm

^{1}H NMR 9p

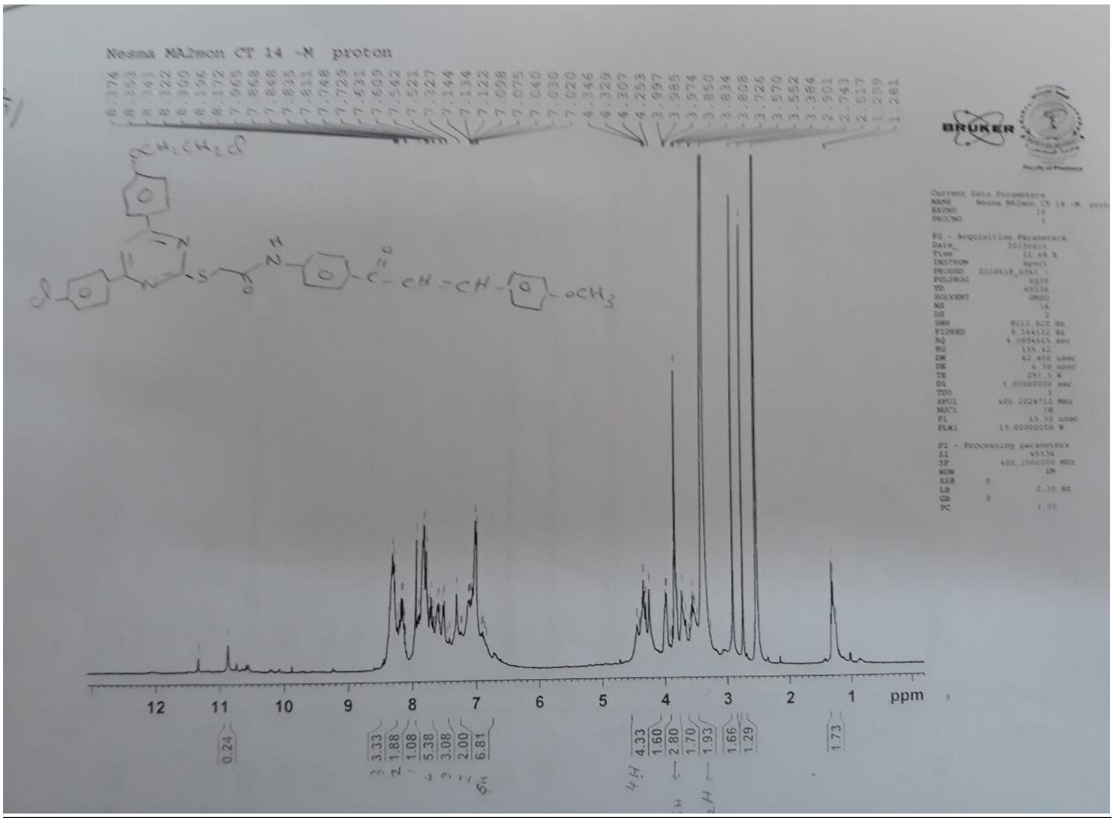

Nesma MA2mon CT 14 -M proton
BRUKER
12
11
10
9
8
7
6
5
4
3
2
1
ppm
0.24
3.33
1.88
1.08
5.38
3.08
2.00
6.81
4.33
1.60
2.80
1.70
1.93
1.66
1.29
1.73

^{13}C NMR 9p

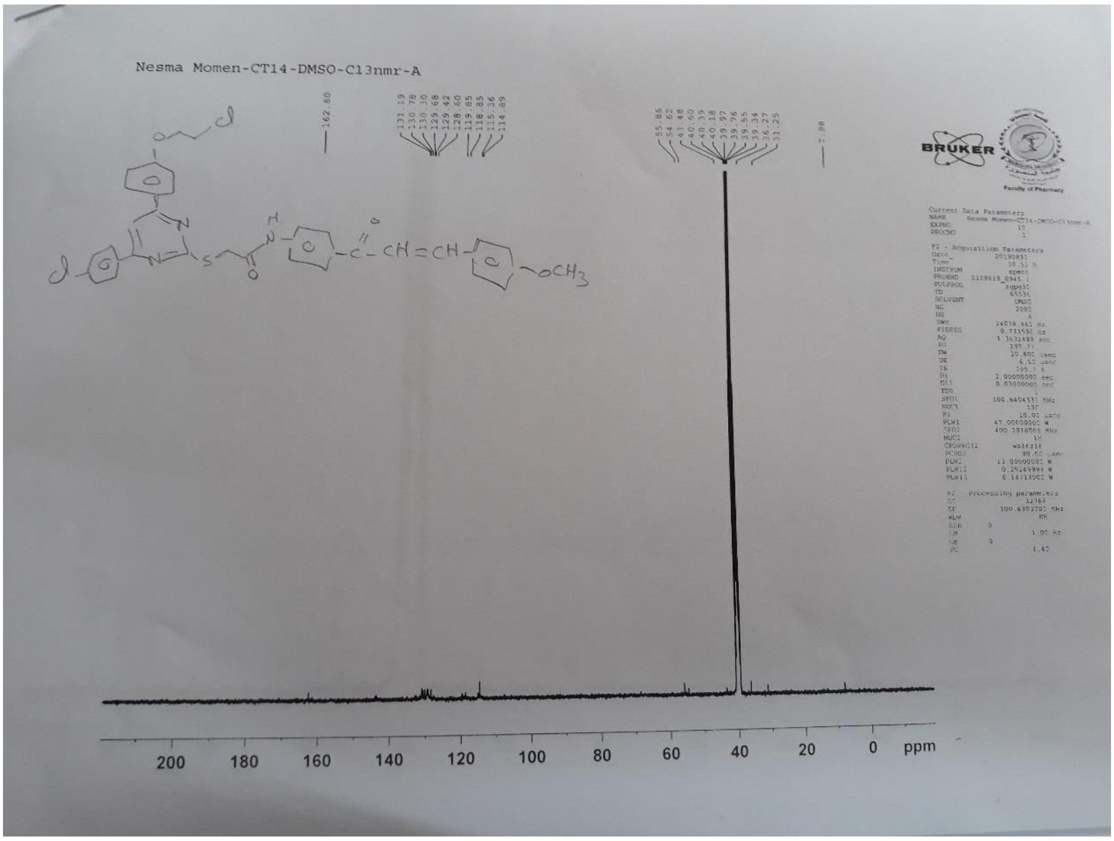
Nesma Momen-CT14-DMSO-C13nmr-A
162.80
131.19
130.78
130.30
129.68
129.42
128.60
119.85
118.85
115.36
114.89
55.86
54.62
43.48
40.60
40.39
40.18
39.97
39.76
39.55
39.34
36.27
31.25
7.98
BRUKER
200
180
160
140
120
100
80
60
40
20
0
ppm

^{1}H NMR 9q

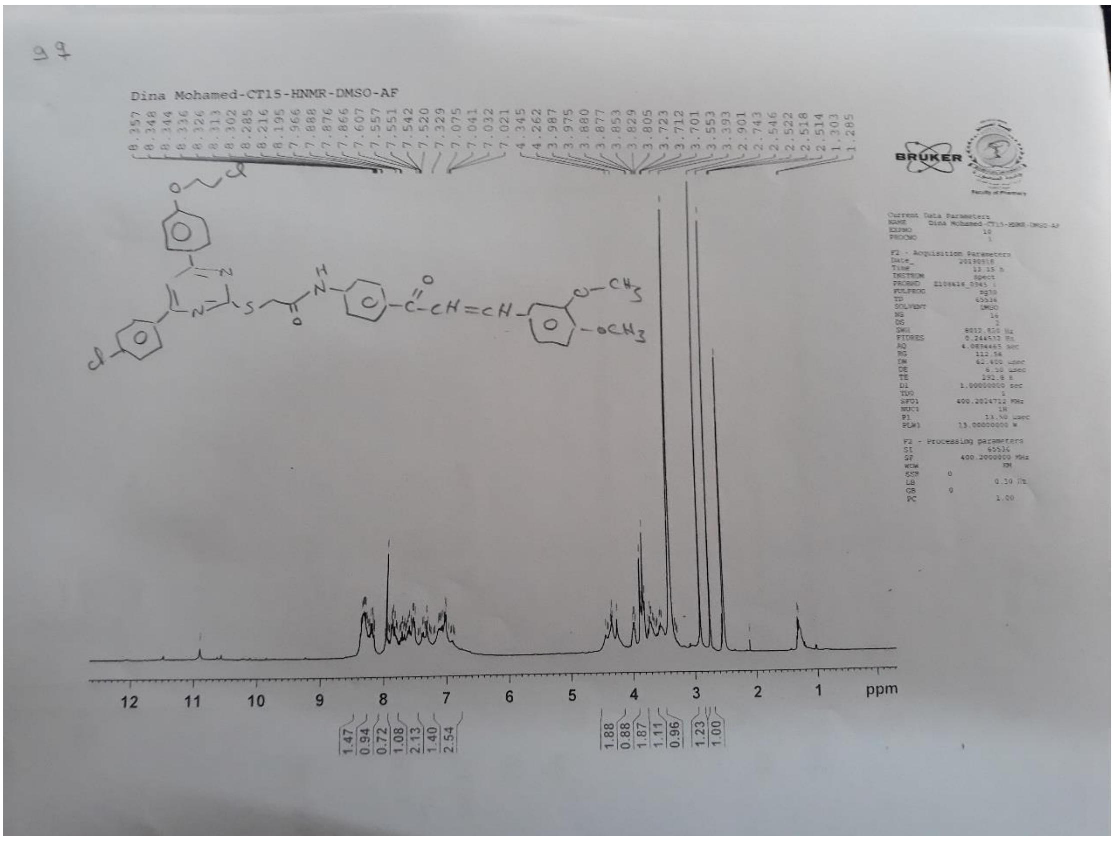

^{13}C NMR 9q

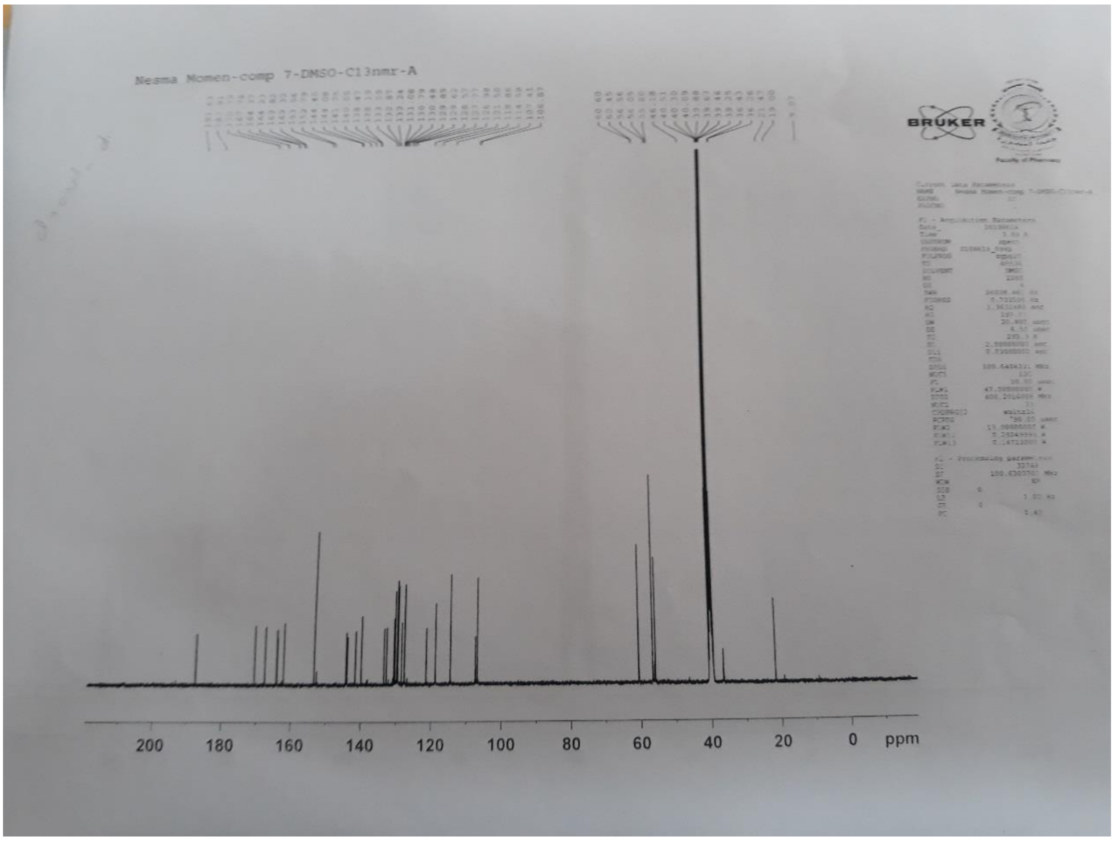

^{1}H NMR 9r

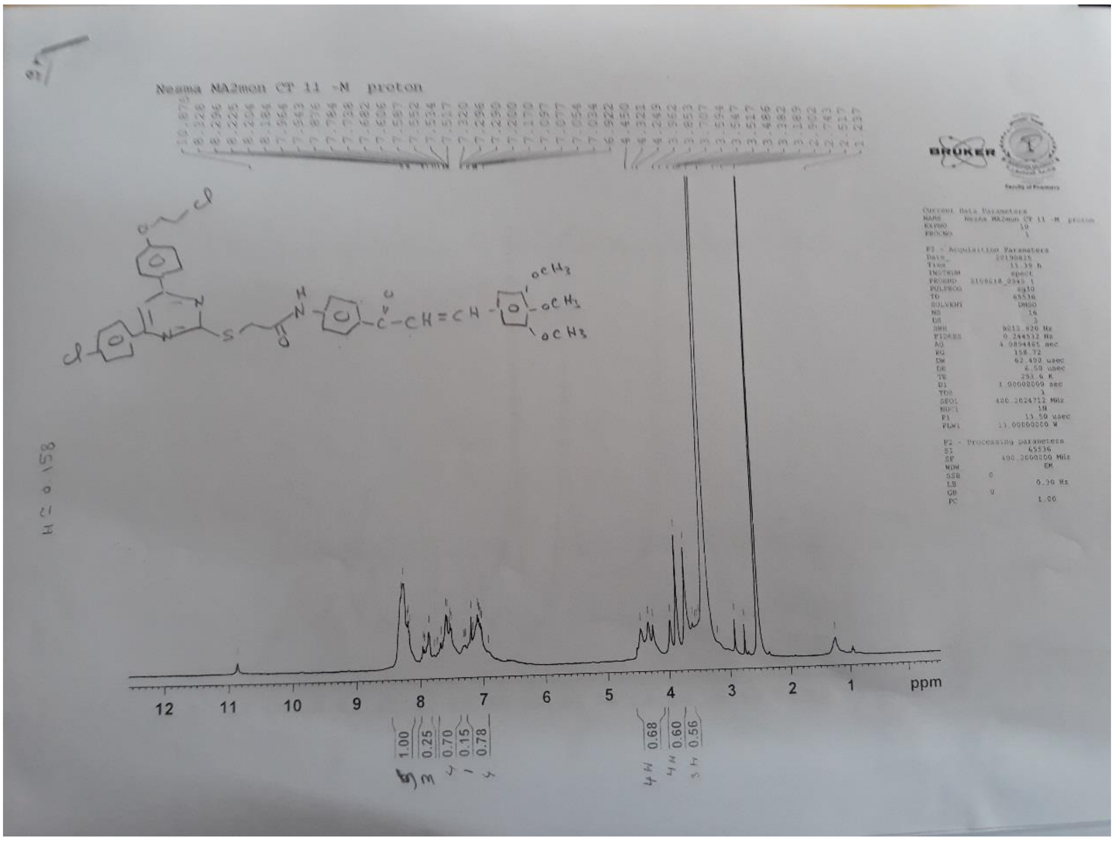

Nesma NA2mon CT 11 -N proton
BRUKER
12
11
10
9
8
7
6
5
4
3
2
1
ppm
1.00
0.25
0.70
0.15
0.78
0.68
0.60
0.56

^{13}C NMR 9r

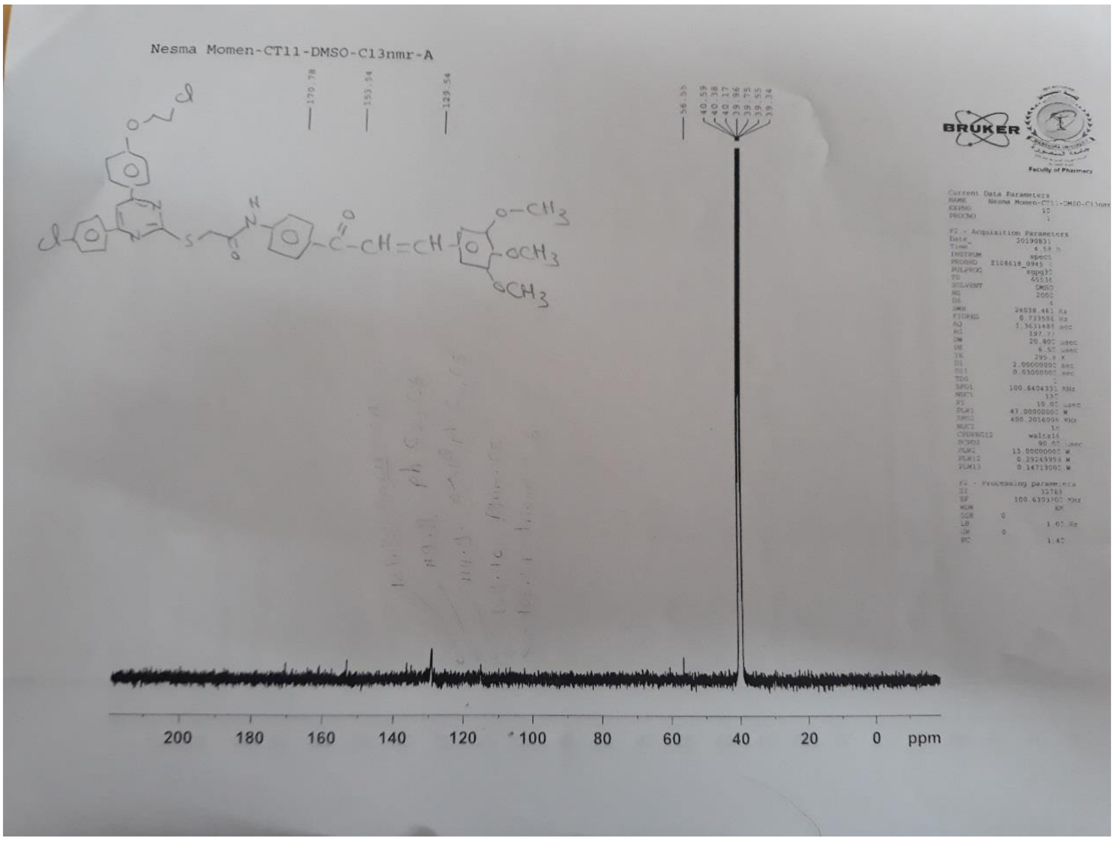
Nesma Momen-CT11-DMSO-C13nmr-A
170.78
153.54
129.54
56.55
40.59
40.38
40.17
39.96
39.75
39.55
39.34
BRUKER
Faculty of Pharmacy
Current Data Parameters
200
180
160
140
120
100
80
60
40
20
0
ppm

IR – 9a

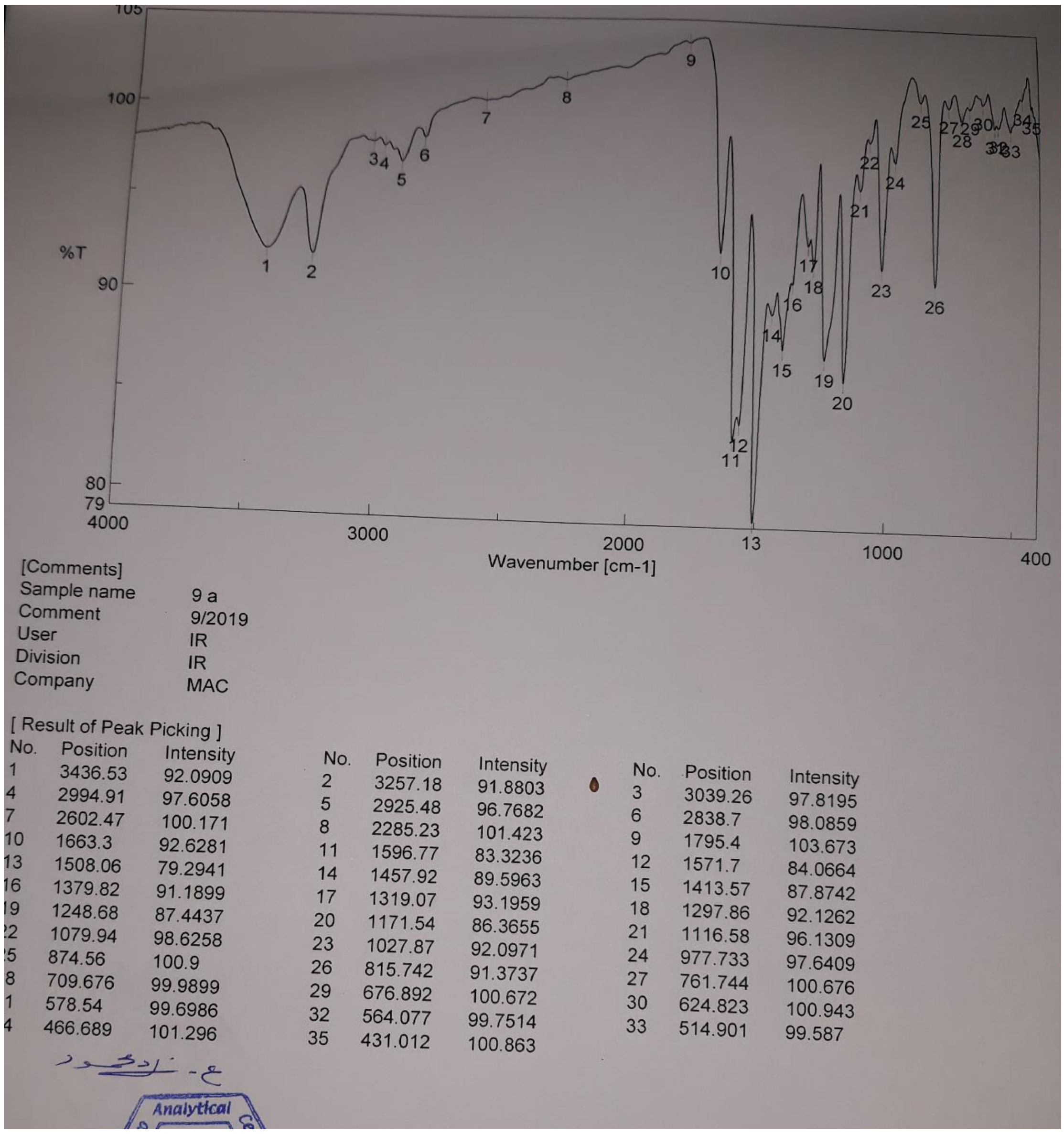

[Comments]

Sample name 9 a
Comment 9/2019
User IR
Division IR
Company MAC

[Result of Peak Picking]

No.	Position	Intensity	No.	Position	Intensity	No.	Position	Intensity
1	3436.53	92.0909	2	3257.18	91.8803	3	3039.26	97.8195
4	2994.91	97.6058	5	2925.48	96.7682	6	2838.7	98.0859
7	2602.47	100.171	8	2285.23	101.423	9	1795.4	103.673
10	1663.3	92.6281	11	1596.77	83.3236	12	1571.7	84.0664
13	1508.06	79.2941	14	1457.92	89.5963	15	1413.57	87.8742
16	1379.82	91.1899	17	1319.07	93.1959	18	1297.86	92.1262
19	1248.68	87.4437	20	1171.54	86.3655	21	1116.58	96.1309
2	1079.94	98.6258	23	1027.87	92.0971	24	977.733	97.6409
5	874.56	100.9	26	815.742	91.3737	27	761.744	100.676
8	709.676	99.9899	29	676.892	100.672	30	624.823	100.943
1	578.54	99.6986	32	564.077	99.7514	33	514.901	99.587
4	466.689	101.296	35	431.012	100.863			

IR – 9d

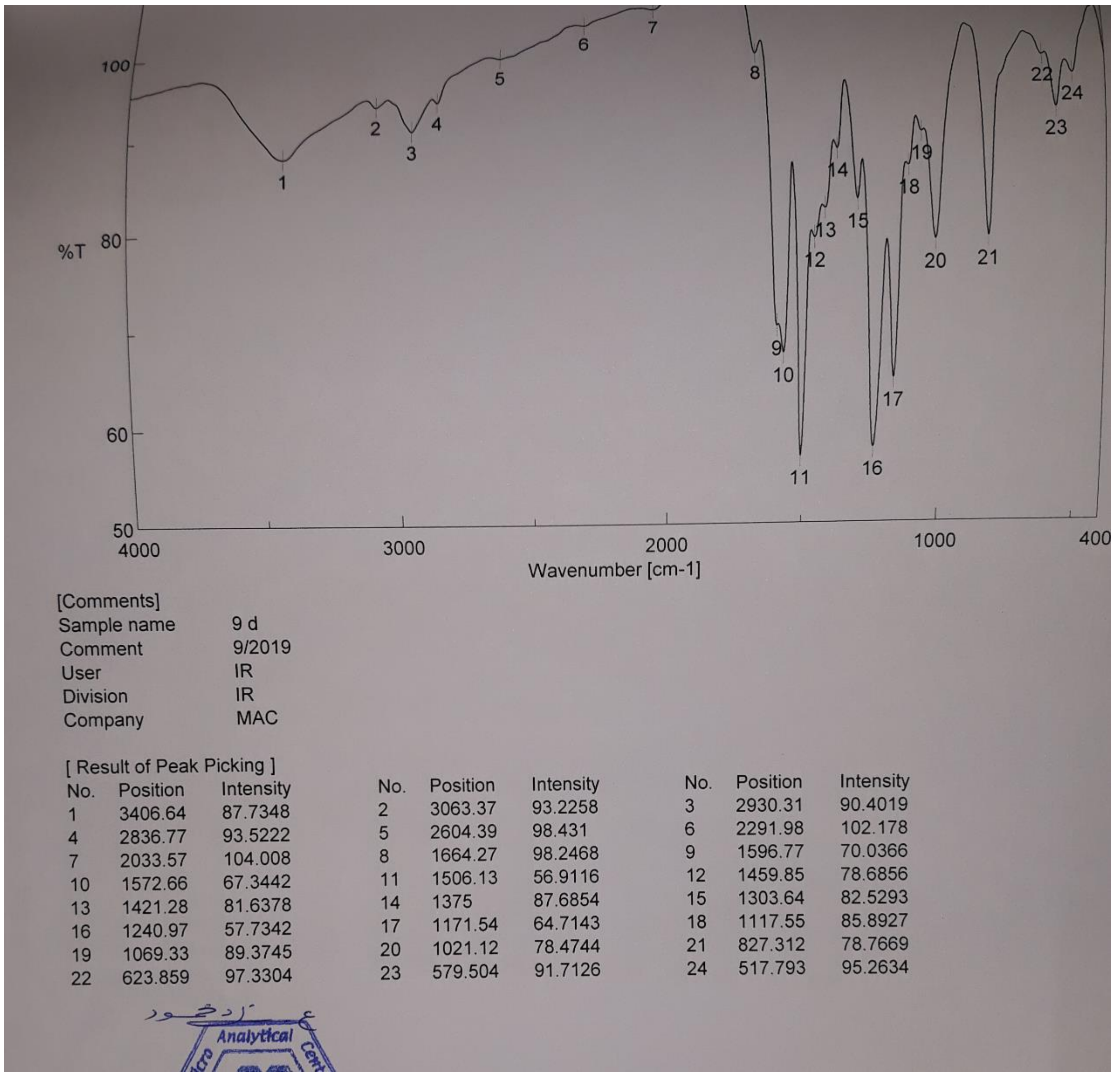

[Comments]
Sample name 9 d
Comment 9/2019
User IR
Division IR
Company MAC

[Result of Peak Picking]

No.	Position	Intensity	No.	Position	Intensity	No.	Position	Intensity
1	3406.64	87.7348	2	3063.37	93.2258	3	2930.31	90.4019
4	2836.77	93.5222	5	2604.39	98.431	6	2291.98	102.178
7	2033.57	104.008	8	1664.27	98.2468	9	1596.77	70.0366
10	1572.66	67.3442	11	1506.13	56.9116	12	1459.85	78.6856
13	1421.28	81.6378	14	1375	87.6854	15	1303.64	82.5293
16	1240.97	57.7342	17	1171.54	64.7143	18	1117.55	85.8927
19	1069.33	89.3745	20	1021.12	78.4744	21	827.312	78.7669
22	623.859	97.3304	23	579.504	91.7126	24	517.793	95.2634

Analytical

IR – 9h

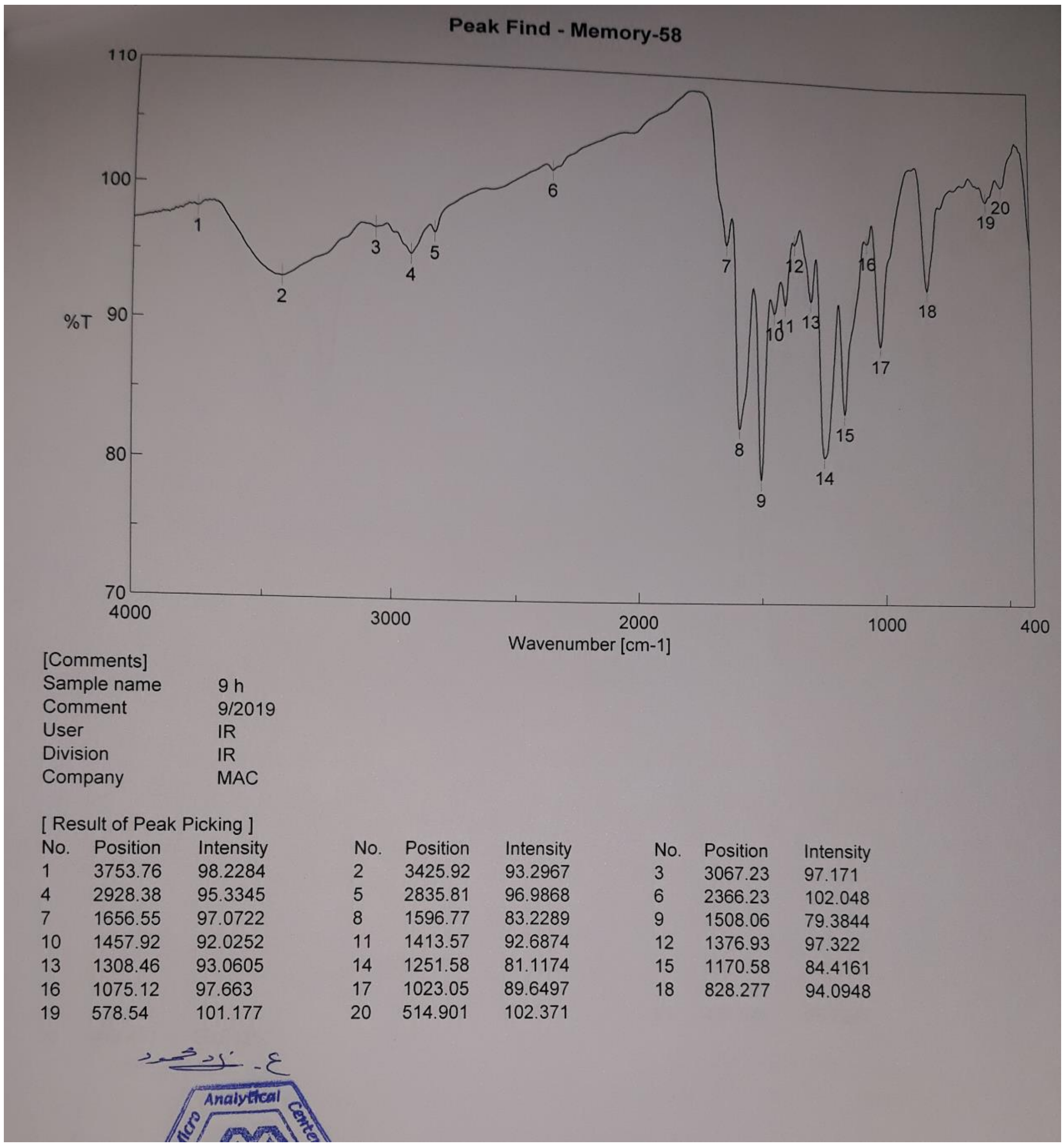

[Comments]

Sample name	9 h
Comment	9/2019
User	IR
Division	IR
Company	MAC

[Result of Peak Picking]

No.	Position	Intensity	No.	Position	Intensity	No.	Position	Intensity
1	3753.76	98.2284	2	3425.92	93.2967	3	3067.23	97.171
4	2928.38	95.3345	5	2835.81	96.9868	6	2366.23	102.048
7	1656.55	97.0722	8	1596.77	83.2289	9	1508.06	79.3844
10	1457.92	92.0252	11	1413.57	92.6874	12	1376.93	97.322
13	1308.46	93.0605	14	1251.58	81.1174	15	1170.58	84.4161
16	1075.12	97.663	17	1023.05	89.6497	18	828.277	94.0948
19	578.54	101.177	20	514.901	102.371			

IR – 9j

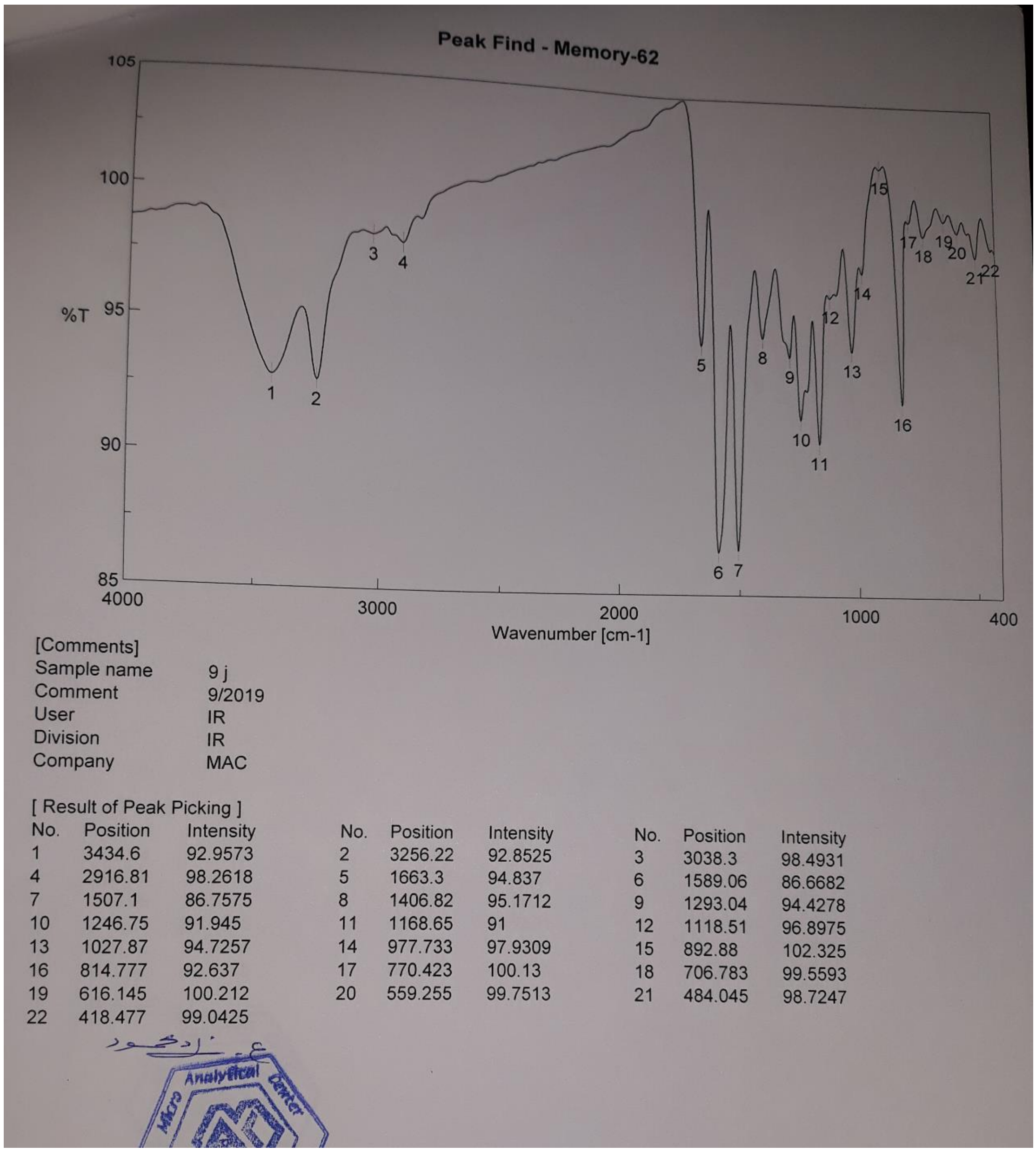

[Comments]

Sample name	9 j
Comment	9/2019
User	IR
Division	IR
Company	MAC

[Result of Peak Picking]

No.	Position	Intensity	No.	Position	Intensity	No.	Position	Intensity
1	3434.6	92.9573	2	3256.22	92.8525	3	3038.3	98.4931
4	2916.81	98.2618	5	1663.3	94.837	6	1589.06	86.6682
7	1507.1	86.7575	8	1406.82	95.1712	9	1293.04	94.4278
10	1246.75	91.945	11	1168.65	91	12	1118.51	96.8975
13	1027.87	94.7257	14	977.733	97.9309	15	892.88	102.325
16	814.777	92.637	17	770.423	100.13	18	706.783	99.5593
19	616.145	100.212	20	559.255	99.7513	21	484.045	98.7247
22	418.477	99.0425						

Micro Analytical Center

IR – 9m

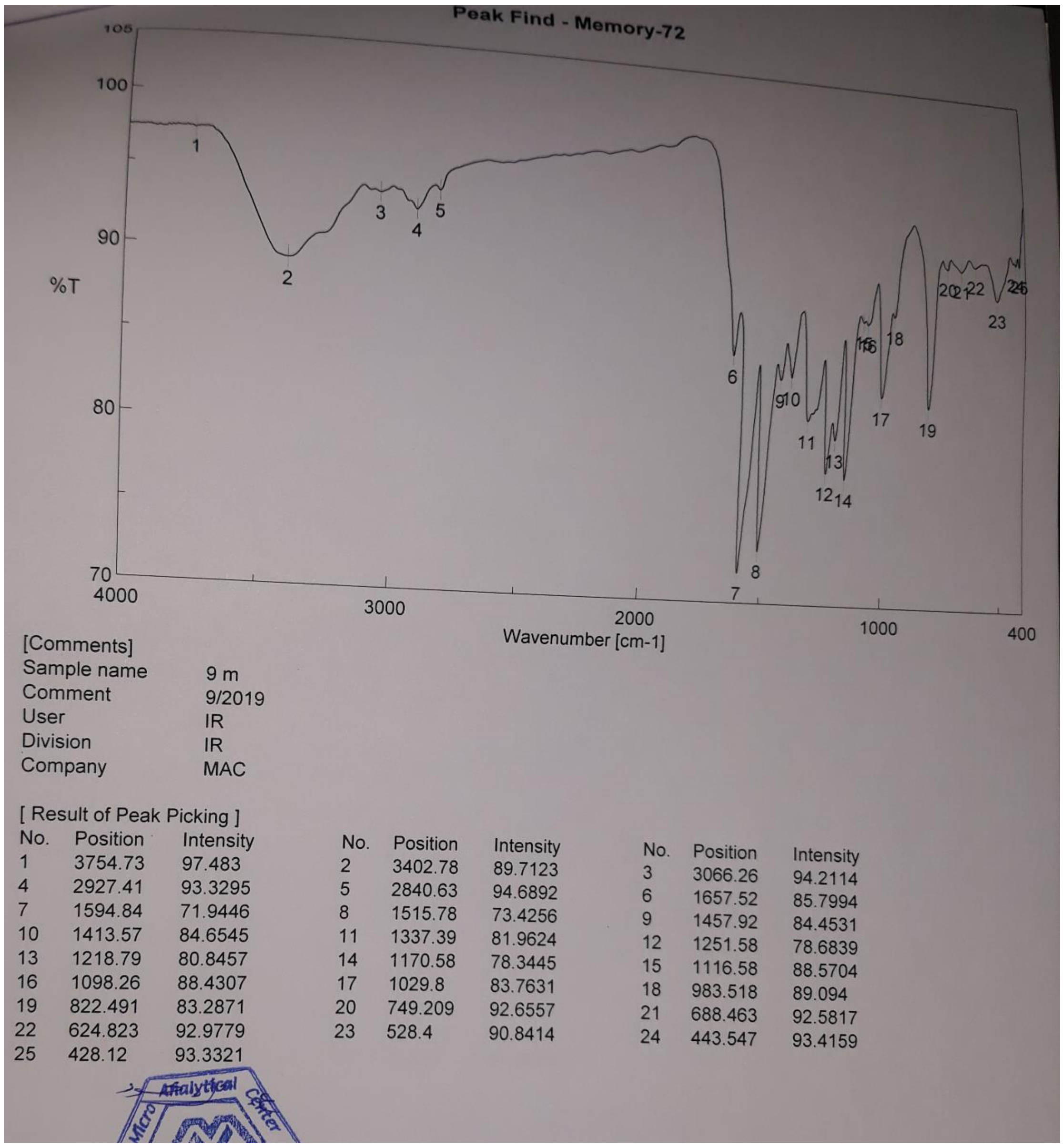

[Comments]

Sample name	9 m
Comment	9/2019
User	IR
Division	IR
Company	MAC

[Result of Peak Picking]

No.	Position	Intensity	No.	Position	Intensity	No.	Position	Intensity
1	3754.73	97.483	2	3402.78	89.7123	3	3066.26	94.2114
4	2927.41	93.3295	5	2840.63	94.6892	6	1657.52	85.7994
7	1594.84	71.9446	8	1515.78	73.4256	9	1457.92	84.4531
10	1413.57	84.6545	11	1337.39	81.9624	12	1251.58	78.6839
13	1218.79	80.8457	14	1170.58	78.3445	15	1116.58	88.5704
16	1098.26	88.4307	17	1029.8	83.7631	18	983.518	89.094
19	822.491	83.2871	20	749.209	92.6557	21	688.463	92.5817
22	624.823	92.9779	23	528.4	90.8414	24	443.547	93.4159
25	428.12	93.3321						

IR – 9q

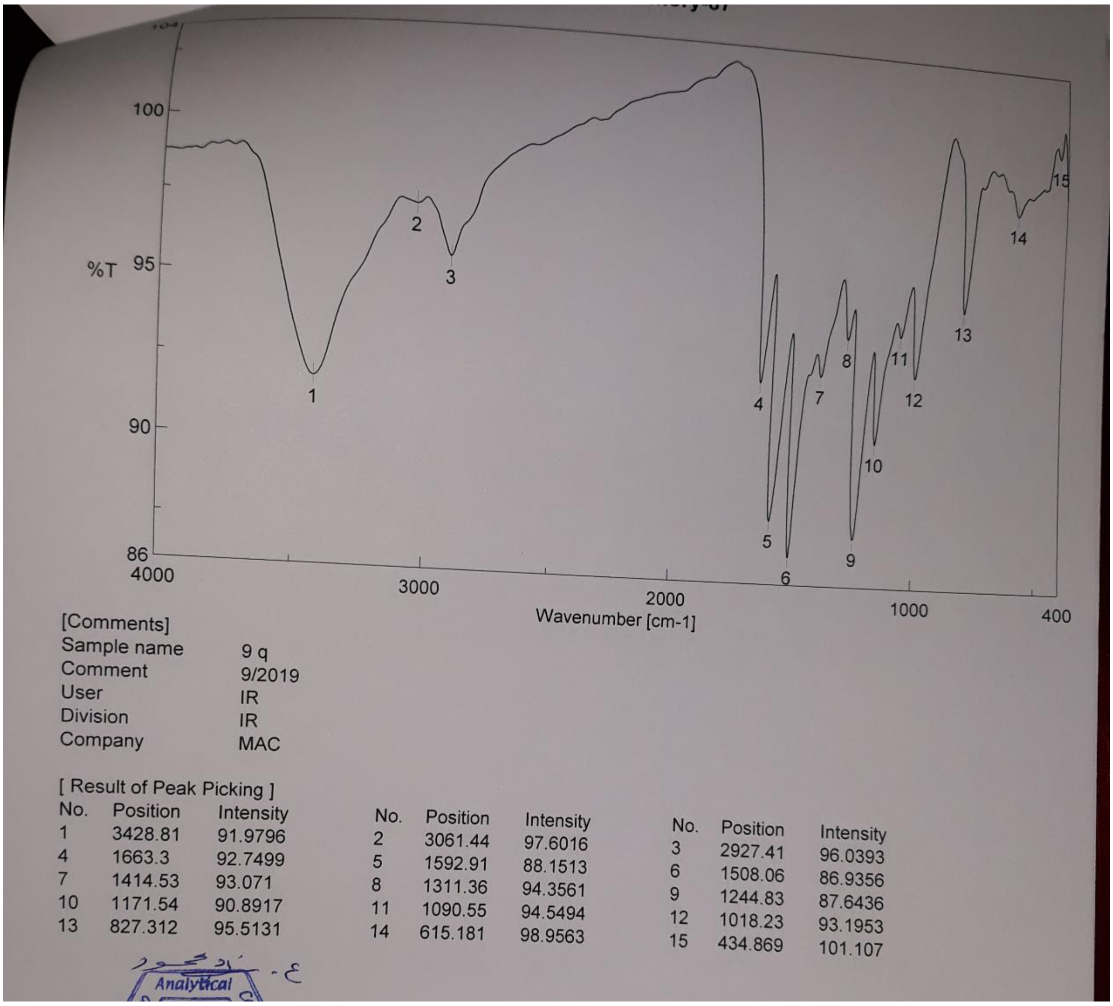

[Comments]

Sample name	9 q
Comment	9/2019
User	IR
Division	IR
Company	MAC

[Result of Peak Picking]

No.	Position	Intensity	No.	Position	Intensity	No.	Position	Intensity
1	3428.81	91.9796	2	3061.44	97.6016	3	2927.41	96.0393
4	1663.3	92.7499	5	1592.91	88.1513	6	1508.06	86.9356
7	1414.53	93.071	8	1311.36	94.3561	9	1244.83	87.6436
10	1171.54	90.8917	11	1090.55	94.5494	12	1018.23	93.1953
13	827.312	95.5131	14	615.181	98.9563	15	434.869	101.107

Mass- 9c

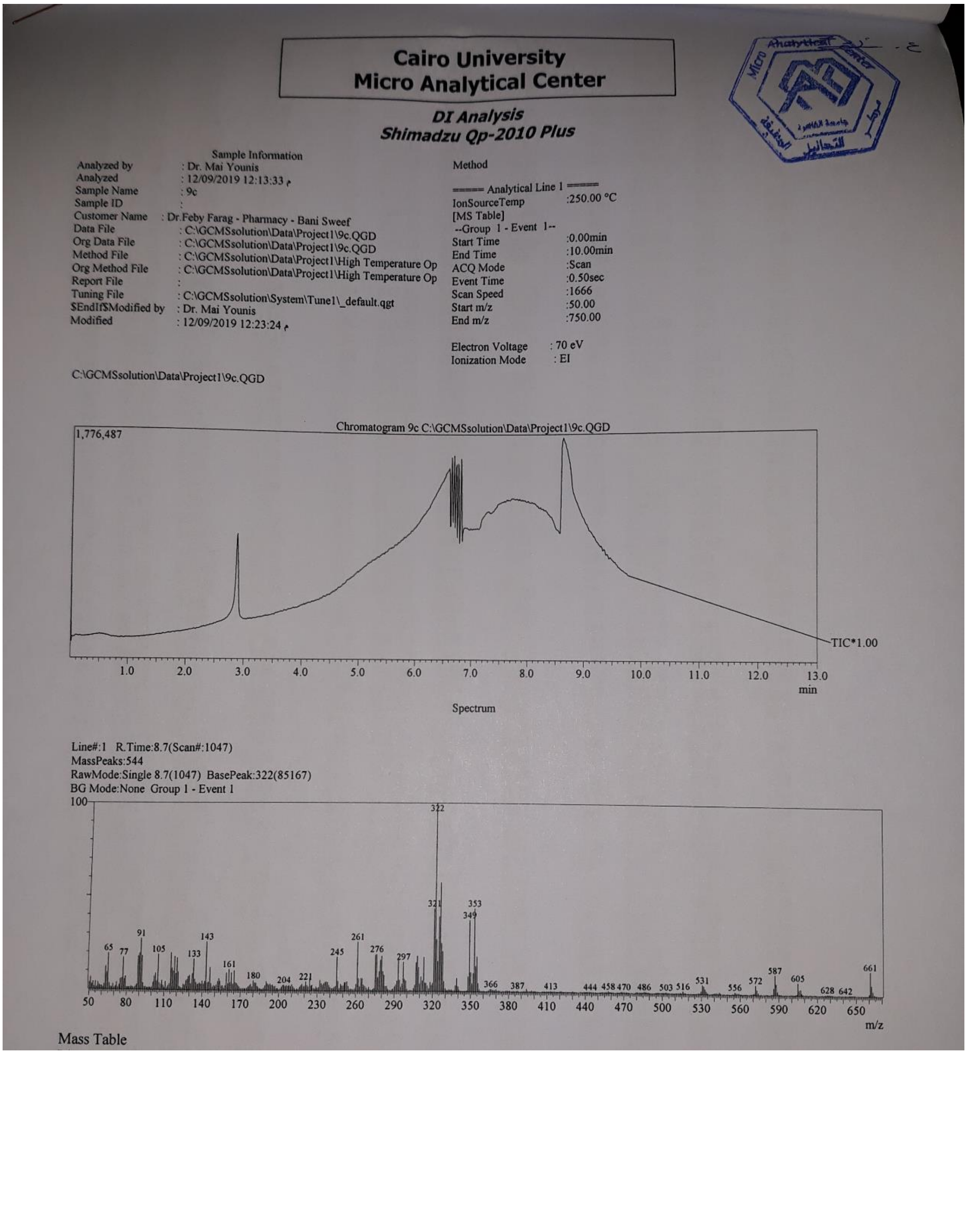
Cairo University
Micro Analytical Center
DI Analysis
Shimadzu Qp-2010 Plus
Sample Information
Analyzed by : Dr. Mai Younis
Analyzed : 12/09/2019 12:13:33 م
Sample Name : 9c
Sample ID :
Customer Name : Dr.Feby Farag - Pharmacy - Bani Sweef
Data File : C:\GCMSsolution\Data\Project1\9c.QGD
Org Data File : C:\GCMSsolution\Data\Project1\9c.QGD
Method File : C:\GCMSsolution\Data\Project1\High Temperature Op
Org Method File : C:\GCMSsolution\Data\Project1\High Temperature Op
Report File :
Tuning File : C:\GCMSsolution\System\Tune1_default.qgt
$EndIf$Modified by : Dr. Mai Younis
Modified : 12/09/2019 12:23:24 م
Method
===== Analytical Line 1 =====
IonSourceTemp :250.00 °C
[MS Table]
--Group 1 - Event 1--
Start Time :0.00min
End Time :10.00min
ACQ Mode :Scan
Event Time :0.50sec
Scan Speed :1666
Start m/z :50.00
End m/z :750.00
Electron Voltage : 70 eV
Ionization Mode : EI
C:\GCMSsolution\Data\Project1\9c.QGD
Chromatogram 9c C:\GCMSsolution\Data\Project1\9c.QGD
1,776,487
TIC*1.00
1.0 2.0 3.0 4.0 5.0 6.0 7.0 8.0 9.0 10.0 11.0 12.0 13.0
min
Spectrum
Line#:1 R.Time:8.7(Scan#:1047)
MassPeaks:544
RawMode:Single 8.7(1047) BasePeak:322(85167)
BG Mode:None Group 1 - Event 1
100
322
321
353
349
91
143
261
65
77
105
133
245
276
297
161
180
204
221
366
387
413
444 458 470 486 503 516
531
556
572
587
605
628 642
661
50 80 110 140 170 200 230 260 290 320 350 380 410 440 470 500 530 560 590 620 650
m/z
Mass Table

Mass- 9f

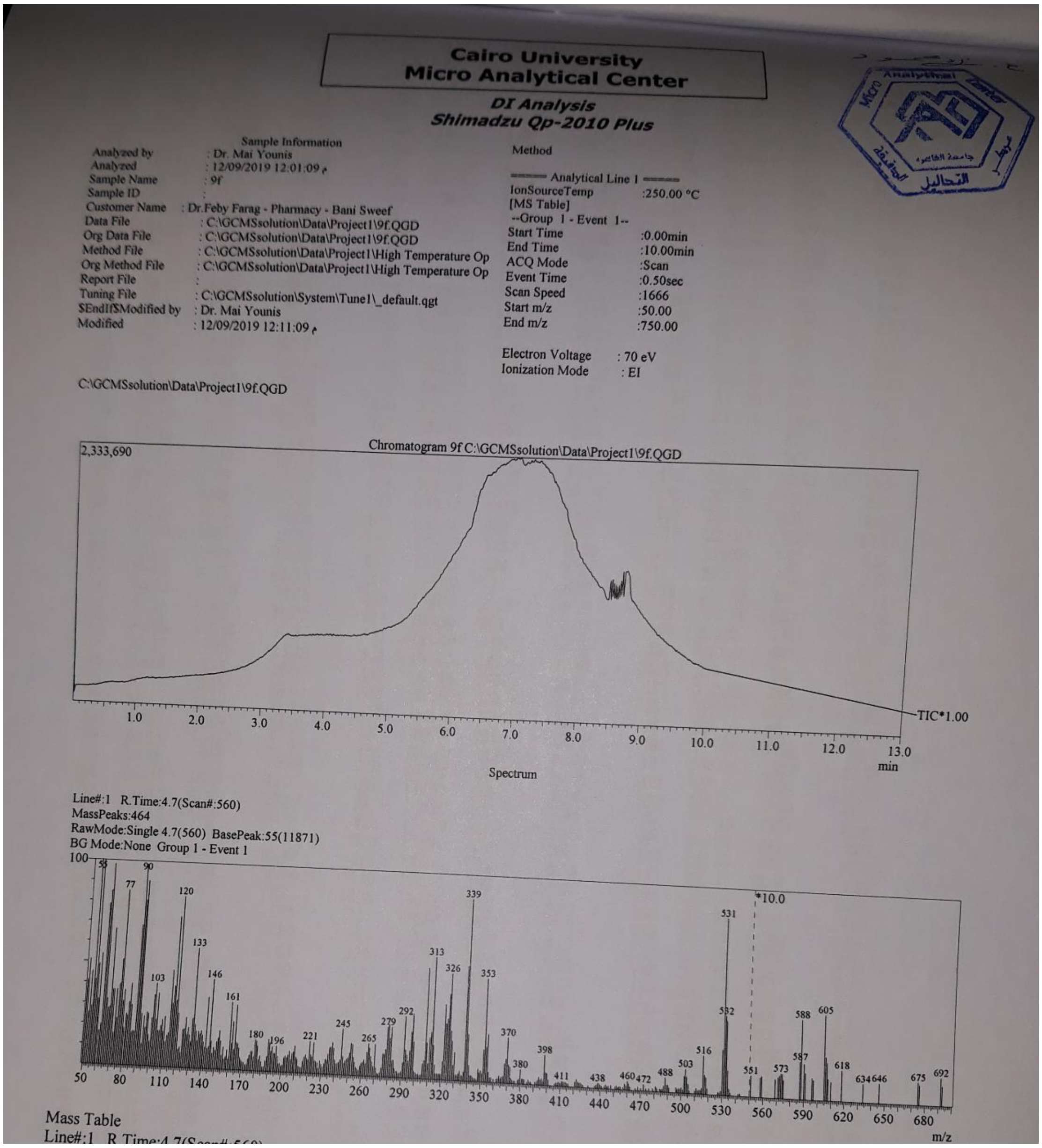

Cairo University
Micro Analytical Center

DI Analysis
Shimadzu Qp-2010 Plus

Sample Information

Analyzed by	: Dr. Mai Younis
Analyzed	: 12/09/2019 12:01:09 م
Sample Name	: 9f
Sample ID	:
Customer Name	: Dr.Feby Farag - Pharmacy - Bani Sweef
Data File	: C:\GCMSsolution\Data\Project1\9f.QGD
Org Data File	: C:\GCMSsolution\Data\Project1\9f.QGD
Method File	: C:\GCMSsolution\Data\Project1\High Temperature Op
Org Method File	: C:\GCMSsolution\Data\Project1\High Temperature Op
Report File	:
Tuning File	: C:\GCMSsolution\System\Tune1_default.qgt
$EndIf$Modified by	: Dr. Mai Younis
Modified	: 12/09/2019 12:11:09 م

Method

===== Analytical Line 1 =====

IonSourceTemp	:250.00 °C
[MS Table]	
--Group 1 - Event 1--	
Start Time	:0.00min
End Time	:10.00min
ACQ Mode	:Scan
Event Time	:0.50sec
Scan Speed	:1666
Start m/z	:50.00
End m/z	:750.00
Electron Voltage	: 70 eV
Ionization Mode	: EI

C:\GCMSsolution\Data\Project1\9f.QGD

Mass Table
Line#:1 R.Time:4.7(Scan#:560)

Mass- 9j

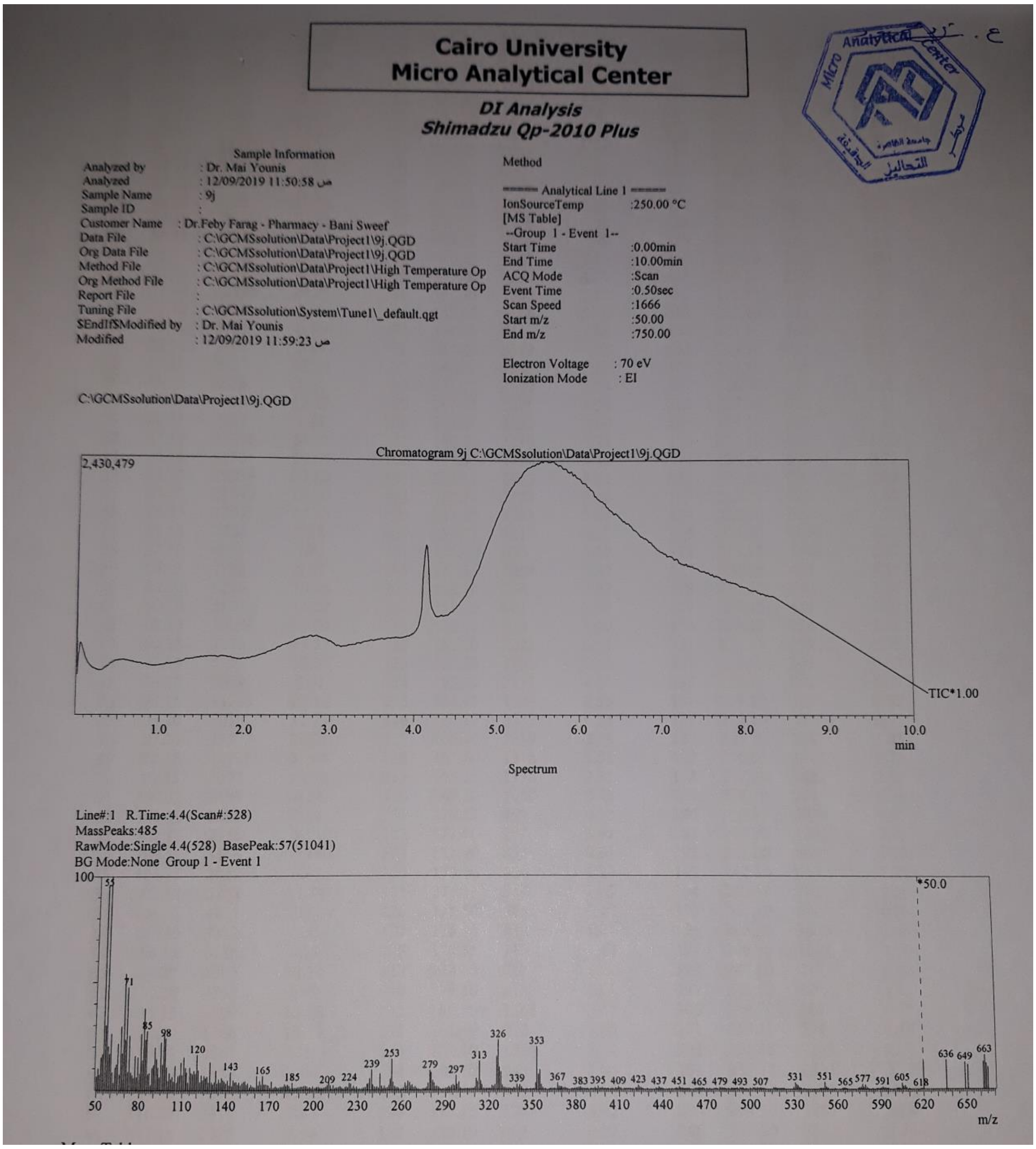

Cairo University
Micro Analytical Center

DI Analysis
Shimadzu Qp-2010 Plus

Sample Information

Analyzed by	: Dr. Mai Younis
Analyzed	: 12/09/2019 11:50:58 ص
Sample Name	: 9j
Sample ID	:
Customer Name	: Dr.Feby Farag - Pharmacy - Bani Sweef
Data File	: C:\GCMSsolution\Data\Project1\9j.QGD
Org Data File	: C:\GCMSsolution\Data\Project1\9j.QGD
Method File	: C:\GCMSsolution\Data\Project1\High Temperature Op
Org Method File	: C:\GCMSsolution\Data\Project1\High Temperature Op
Report File	:
Tuning File	: C:\GCMSsolution\System\Tune1_default.qgt
$EndIf$Modified by	: Dr. Mai Younis
Modified	: 12/09/2019 11:59:23 ص

Method

====== Analytical Line 1 ======

IonSourceTemp	:250.00 °C
[MS Table]	
--Group 1 - Event 1--	
Start Time	:0.00min
End Time	:10.00min
ACQ Mode	:Scan
Event Time	:0.50sec
Scan Speed	:1666
Start m/z	:50.00
End m/z	:750.00
Electron Voltage	: 70 eV
Ionization Mode	: EI

C:\GCMSsolution\Data\Project1\9j.QGD

Spectrum

Line#:1 R.Time:4.4(Scan#:528)
MassPeaks:485
RawMode:Single 4.4(528) BasePeak:57(51041)
BG Mode:None Group 1 - Event 1

Mass- 9r

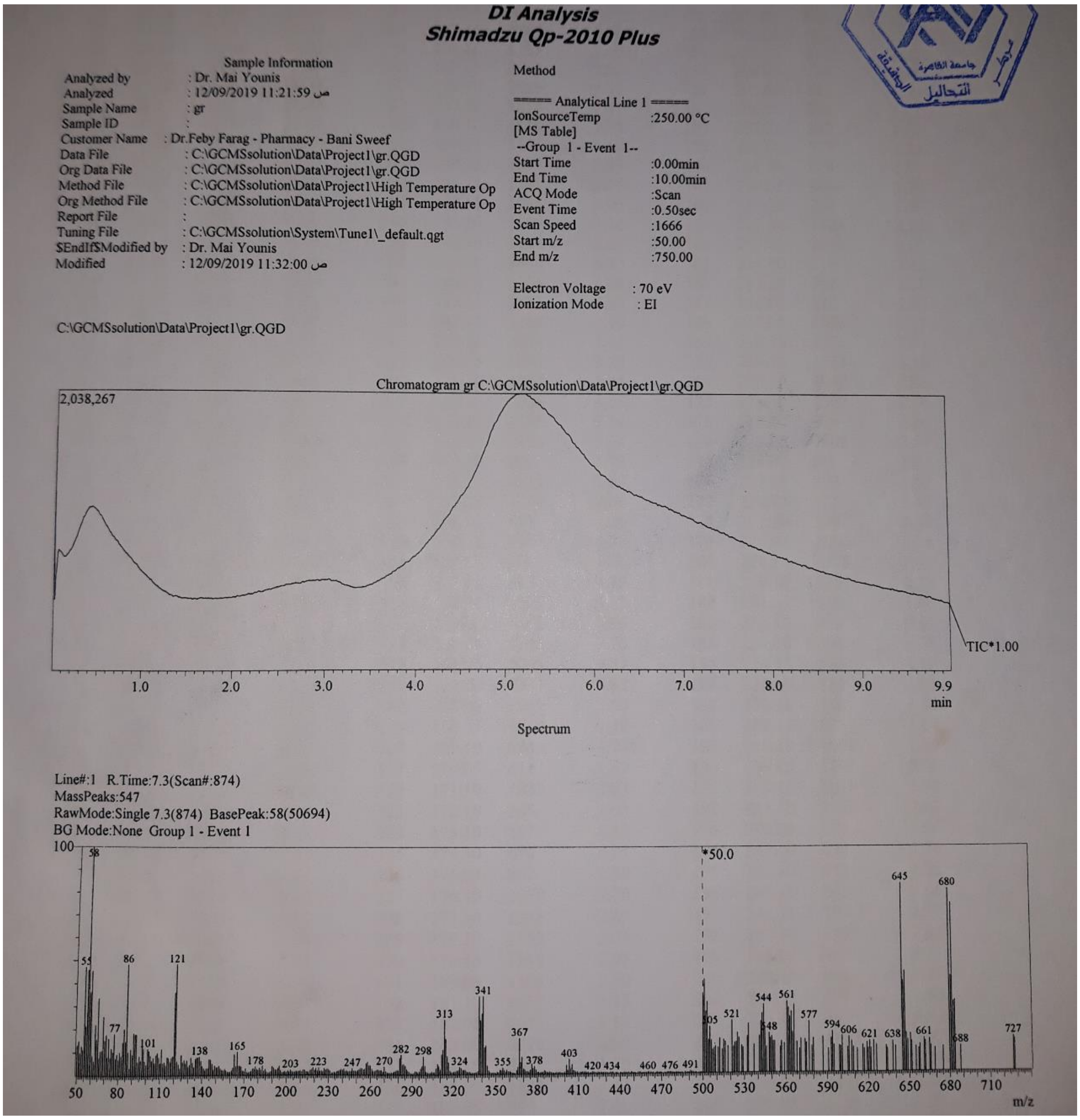

DI Analysis
Shimadzu Qp-2010 Plus

Sample Information

Analyzed by	: Dr. Mai Younis
Analyzed	: 12/09/2019 11:21:59 ص
Sample Name	: gr
Sample ID	:
Customer Name	: Dr.Feby Farag - Pharmacy - Bani Sweef
Data File	: C:\GCMSsolution\Data\Project1\gr.QGD
Org Data File	: C:\GCMSsolution\Data\Project1\gr.QGD
Method File	: C:\GCMSsolution\Data\Project1\High Temperature Op
Org Method File	: C:\GCMSsolution\Data\Project1\High Temperature Op
Report File	:
Tuning File	: C:\GCMSsolution\System\Tune1_default.qgt
$EndIf$Modified by	: Dr. Mai Younis
Modified	: 12/09/2019 11:32:00 ص

Method

===== Analytical Line 1 =====

IonSourceTemp	:250.00 °C
[MS Table]	
--Group 1 - Event 1--	
Start Time	:0.00min
End Time	:10.00min
ACQ Mode	:Scan
Event Time	:0.50sec
Scan Speed	:1666
Start m/z	:50.00
End m/z	:750.00
Electron Voltage	: 70 eV
Ionization Mode	: EI

C:\GCMSsolution\Data\Project1\gr.QGD

Spectrum

Line#:1 R.Time:7.3(Scan#:874)
MassPeaks:547
RawMode:Single 7.3(874) BasePeak:58(50694)
BG Mode:None Group 1 - Event 1
